# Supplementary material for: Combined Analysis of Second- and Third-Generation Transcriptome Sequencing for Gene Characteristics and Identification of Key Splicing Variants in Wound Healing of Ganxi Goat Skin
Source: Animals (Basel). 2024 Oct 26;14(21):3085. doi: 10.3390/ani14213085 (PMC11544938; doi:10.3390/ani14213085)
Supplement: Supplementary file 1 [file animals-14-03085-s001.zip › animals-3219534-supplementary.pdf]

Supplementary Materials

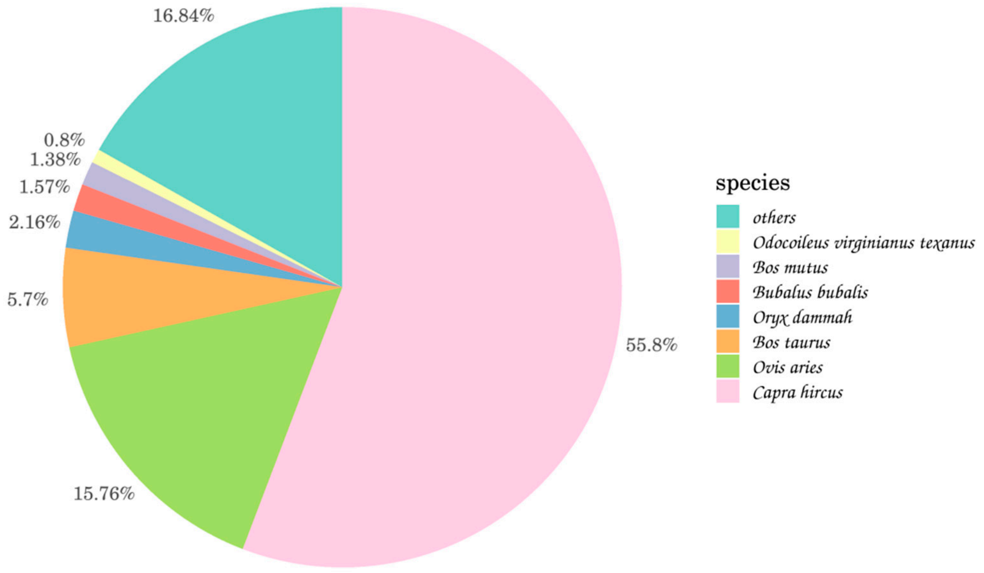

Figure S1: NR Annotation Statistics.

Table S1: ROI classification statistics.

**Category**	Number
Number of ROI Sequences	1,010,225
Total Non-Full-Length Sequences	82,968
Total Full-Length Chimeric Sequences	590
Total Full-Length Non-Chimeric Sequences	926,667
Full-Length Non-Chimeric Sequences without polyA Tail	1223
Full-Length Non-Chimeric Sequences with polyA Tail	925,444

Table S2. HQ isoform statistics.

**Sample**	**Total Number of Sequences Used for Alignment (HQ Isoforms)**	**Number of Sequences Aligned to Reference Genome**	**Total Alignment Rate (%)**	**Number of Sequences Aligned to Regions Less Than 99%**	**Percentage of Sequences Aligned to Regions Less Than 99% of Total Aligned Sequences (%)**
Skin	56,981	52,791	92.65	1302	2.47

Table S3: Annotated genetic statistics.

**Classification**	Number
Annotated Genes	10,907
Novel Genes	2834
Optimized Gene Loci	3794

Table S4: Information of newly identified novel genes

isoform	chrom	strand	length	exons	structural_ category	associated_gene	start	end
PB.10.1	1	-	2443	1	intergenic	novelGene_1	585030	587472
PB.104.1	1	-	1515	1	intergenic	novelGene_26	46021423	46022937
PB.105.4	1	+	2422	1	intergenic	novelGene_27	49852099	49854520
PB.105.5	1	+	2923	1	intergenic	novelGene_28	49866235	49869157
PB.106.1	1	+	2383	1	intergenic	novelGene_30	50318081	50320463
PB.107.2	1	-	1567	1	intergenic	novelGene_29	50292524	50294090
PB.108.1	1	+	1747	2	antisense	novelGene_ENSCHIG00000005821_AS	51688528	51694241
PB.108.2	1	+	1390	2	antisense	novelGene_ENSCHIG00000005821_AS	51688551	51693907
PB.11.1	1	-	2467	1	intergenic	novelGene_2	601443	603909
PB.117.1	1	-	1337	5	intergenic	novelGene_31	56854399	56867360
PB.127.1	1	-	1631	1	intergenic	novelGene_32	58109346	58110976
PB.133.1	1	-	1372	1	intergenic	novelGene_33	58736851	58738222
PB.135.1	1	-	2420	1	intergenic	novelGene_34	59056837	59059256
PB.136.1	1	+	1388	1	intergenic	novelGene_35	59110135	59111522
PB.137.1	1	-	1370	1	intergenic	novelGene_36	59487375	59488744
PB.140.2	1	+	1578	1	intergenic	novelGene_37	63899503	63901080
PB.143.1	1	-	3110	1	intergenic	novelGene_38	63980798	63983907
PB.147.4	1	-	1808	1	intergenic	novelGene_39	64374712	64376519
PB.15.1	1	+	1866	2	intergenic	novelGene_3	1203393	1224082
PB.15.2	1	+	1517	2	intergenic	novelGene_4	1222452	1224062
PB.16.1	1	-	1270	1	antisense	novelGene_ENSCHIG00000021245_AS	1271629	1272898
PB.167.1	1	+	4456	1	intergenic	novelGene_40	66875701	66880156
PB.175.1	1	-	2516	1	intergenic	novelGene_41	69184271	69186786
PB.176.1	1	+	1379	1	intergenic	novelGene_42	69495363	69496741
PB.18.1	1	+	1360	1	intergenic	novelGene_5	1375202	1376561
PB.180.1	1	-	505	6	antisense	novelGene_ENSCHIG00000010981_AS	69937574	69941614
PB.180.3	1	-	428	5	antisense	novelGene_ENSCHIG00000010981_AS	69937576	69941614
PB.185.3	1	-	3549	1	intergenic	novelGene_43	70433476	70437024
PB.186.2	1	-	2706	1	intergenic	novelGene_44	70580071	70582776

PB.188.1	1	-	2603	1	intergenic	novelGene_45	70761707	70764309
PB.194.2	1	+	2887	1	intergenic	novelGene_46	70990531	70993417
PB.198.10	1	-	5569	1	intergenic	novelGene_47	71169129	71174697
PB.205.2	1	-	710	1	intergenic	novelGene_48	72600216	72600925
PB.207.1	1	+	1249	1	intergenic	novelGene_49	72865361	72866609
PB.212.1	1	-	1918	1	antisense	novelGene_ENSCHIG00000020817_AS	74365385	74367302
PB.213.1	1	-	1946	1	intergenic	novelGene_50	75696858	75698803
PB.215.1	1	+	3254	1	intergenic	novelGene_52	76497781	76501034
PB.216.5	1	-	1560	1	intergenic	novelGene_51	76480559	76482118
PB.218.2	1	+	3555	1	intergenic	novelGene_53	76964306	76967860
PB.218.3	1	+	2211	1	intergenic	novelGene_54	77035575	77037785
PB.219.1	1	-	2433	2	intergenic	novelGene_55	77095678	77101142
PB.220.12	1	-	2830	1	intergenic	novelGene_56	77265561	77268390
PB.220.13	1	-	1741	1	intergenic	novelGene_57	77270149	77271889
PB.220.14	1	-	1543	1	intergenic	novelGene_58	77270344	77271886
PB.222.1	1	-	3632	1	intergenic	novelGene_59	78149435	78153066
PB.222.2	1	-	1814	1	intergenic	novelGene_60	78149437	78151250
PB.223.1	1	-	1645	1	intergenic	novelGene_61	78153140	78154784
PB.224.1	1	-	1796	1	intergenic	novelGene_62	78163240	78165035
PB.225.1	1	+	1666	1	intergenic	novelGene_63	78566829	78568494
PB.226.10	1	-	1661	1	intergenic	novelGene_64	78794533	78796193
PB.227.1	1	-	2461	1	intergenic	novelGene_65	79291625	79294085
PB.228.1	1	+	2182	1	intergenic	novelGene_66	79294510	79296691
PB.228.2	1	+	1855	1	intergenic	novelGene_67	79294520	79296374
PB.229.1	1	+	1648	1	intergenic	novelGene_68	79300460	79302107
PB.23.1	1	+	2483	3	antisense	novelGene_ENSCHIG00000014504_AS	2015853	2020577
PB.26.6	1	+	1675	1	intergenic	novelGene_6	2775783	2777457
PB.262.7	1	-	2709	1	intergenic	novelGene_69	83166141	83168849
PB.262.8	1	-	2467	1	intergenic	novelGene_70	83166373	83168839
PB.262.9	1	-	1978	1	intergenic	novelGene_71	83166872	83168849
PB.264.1	1	-	1230	6	intergenic	novelGene_72	83319440	83327039
PB.265.1	1	+	1386	1	antisense	novelGene_ENSCHIG00000005980_AS	83517105	83518490
PB.270.1	1	+	1983	1	intergenic	novelGene_73	83913522	83915504
PB.273.1	1	+	1282	1	intergenic	novelGene_74	85761514	85762795
PB.275.1	1	+	2389	1	antisense	novelGene_ENSCHIG00000013993_AS	86090846	86093234
PB.286.1	1	-	2431	1	intergenic	novelGene_76	92058023	92060453

PB.286.2	1	-	1291	1	intergenic	novelGene_75	92058023	92059313
PB.287.1	1	-	2057	2	intergenic	novelGene_77	92425237	92518036
PB.289.1	1	+	2088	1	intergenic	novelGene_79	94918122	94920209
PB.29.1	1	+	548	1	intergenic	novelGene_7	3215328	3215875
PB.290.1	1	+	2103	1	intergenic	novelGene_81	95036189	95038291
PB.291.3	1	-	2002	1	intergenic	novelGene_78	94702324	94704325
PB.291.9	1	-	1916	1	intergenic	novelGene_80	94990001	94991916
PB.292.1	1	-	1412	4	intergenic	novelGene_82	95068639	95099997
PB.293.1	1	+	1246	4	intergenic	novelGene_83	96121337	96125705
PB.293.2	1	+	577	4	intergenic	novelGene_84	96121338	96125037
PB.293.3	1	+	512	4	intergenic	novelGene_85	96121338	96125039
PB.298.1	1	+	1381	1	intergenic	novelGene_86	96813386	96814766
PB.298.2	1	+	2255	1	intergenic	novelGene_87	96813866	96816120
PB.304.1	1	-	1420	1	intergenic	novelGene_88	99278069	99279488
PB.307.1	1	-	1232	1	intergenic	novelGene_89	105608101	105609332
PB.309.1	1	-	734	1	intergenic	novelGene_90	106186983	106187716
PB.31.1	1	+	384	1	intergenic	novelGene_8	3675609	3675992
PB.311.1	1	+	1555	1	intergenic	novelGene_91	106511119	106512673
PB.312.1	1	-	1210	1	intergenic	novelGene_92	106511582	106512791
PB.315.1	1	+	1746	1	intergenic	novelGene_93	106612859	106614604
PB.326.1	1	-	2426	1	antisense	novelGene_ENSCHIG00000015405_AS	110006573	110008998
PB.327.1	1	-	2393	1	intergenic	novelGene_94	110106681	110109073
PB.336.1	1	-	1918	1	intergenic	novelGene_95	114974327	114976244
PB.336.2	1	-	946	1	intergenic	novelGene_96	114975906	114976851
PB.337.11	1	-	3285	1	intergenic	novelGene_101	115162101	115165385
PB.337.3	1	-	1686	1	intergenic	novelGene_97	115087963	115089648
PB.337.4	1	-	2120	1	intergenic	novelGene_99	115110723	115112842
PB.337.5	1	-	991	1	intergenic	novelGene_98	115110723	115111713
PB.337.6	1	-	2945	1	intergenic	novelGene_100	115119979	115122923
PB.339.1	1	-	1646	2	intergenic	novelGene_102	116180524	116185818
PB.339.2	1	-	1060	1	intergenic	novelGene_103	116184759	116185818
PB.340.1	1	+	1760	2	antisense	novelGene_ENSCHIG00000014066_AS	116351774	116355182
PB.345.1	1	+	2124	1	antisense	novelGene_ENSCHIG00000023786_AS	117165342	117167465
PB.348.3	1	-	1508	1	intergenic	novelGene_104	117260153	117261660
PB.348.4	1	-	1973	1	intergenic	novelGene_105	117281818	117283790
PB.35.1	1	+	885	1	intergenic	novelGene_9	3995154	3996038
PB.352.6	1	+	1632	1	intergenic	novelGene_106	118119583	118121214
PB.364.1	1	+	2004	3	intergenic	novelGene_107	124465224	124479035

PB.364.2	1	+	2036	3	intergenic	novelGene_108	124465245	124479042
PB.366.1	1	-	3606	1	intergenic	novelGene_109	124632872	124636477
PB.367.1	1	-	1440	1	intergenic	novelGene_110	124955529	124956968
PB.373.1	1	+	1546	1	intergenic	novelGene_111	126226423	126227968
PB.375.4	1	+	2793	1	intergenic	novelGene_112	126457040	126459832
PB.395.5	1	-	2647	1	intergenic	novelGene_113	131861114	131863760
PB.396.5	1	+	972	1	intergenic	novelGene_115	132068152	132069123
PB.396.6	1	+	1821	1	intergenic	novelGene_116	132072901	132074721
PB.397.1	1	-	2032	1	intergenic	novelGene_114	132052179	132054210
PB.398.1	1	-	1517	1	intergenic	novelGene_117	132080692	132082208
PB.401.1	1	-	1970	1	intergenic	novelGene_118	132675127	132677096
PB.411.1	1	+	1205	1	antisense	novelGene_ENSCHIG00000027264_AS	135412262	135413466
PB.415.2	1	-	3907	1	intergenic	novelGene_119	136835106	136839012
PB.417.1	1	-	2444	1	intergenic	novelGene_120	136951304	136953747
PB.420.1	1	+	1498	1	intergenic	novelGene_121	138516860	138518357
PB.44.1	1	-	1896	1	intergenic	novelGene_10	7822904	7824799
PB.45.1	1	+	1542	1	intergenic	novelGene_11	7874323	7875864
PB.45.2	1	+	1212	1	intergenic	novelGene_12	7874332	7875543
PB.454.1	1	-	2198	1	intergenic	novelGene_122	143652009	143654206
PB.463.1	1	+	1094	1	intergenic	novelGene_123	144479940	144481033
PB.466.1	1	-	668	1	intergenic	novelGene_124	144519888	144520555
PB.471.7	1	-	2521	1	intergenic	novelGene_125	144653201	144655721
PB.482.1	1	-	1807	1	intergenic	novelGene_126	145964394	145966200
PB.484.1	1	+	2168	12	intergenic	novelGene_127	146051829	146072323
PB.489.1	1	+	1699	1	intergenic	novelGene_128	146885353	146887051
PB.49.1	1	-	2013	1	intergenic	novelGene_13	8900583	8902595
PB.490.1	1	-	2041	1	intergenic	novelGene_129	146927078	146929118
PB.491.1	1	-	1990	1	intergenic	novelGene_130	146939242	146941231
PB.492.1	1	-	1538	1	intergenic	novelGene_131	146968242	146969779
PB.492.2	1	-	888	1	intergenic	novelGene_132	146968883	146969770
PB.493.1	1	+	2483	1	intergenic	novelGene_133	147053134	147055616
PB.494.1	1	-	1848	2	intergenic	novelGene_134	147140258	147142259
PB.498.2	1	+	2269	1	intergenic	novelGene_135	148396196	148398464
PB.498.3	1	+	2172	2	intergenic	novelGene_136	148396212	148398464
PB.507.1	1	+	2695	1	intergenic	novelGene_137	149635953	149638647
PB.508.4	1	-	3706	1	intergenic	novelGene_138	150690086	150693791
PB.510.1	1	+	2386	1	intergenic	novelGene_139	151251787	151254172
PB.511.1	1	+	2571	3	intergenic	novelGene_140	151324430	151338947
PB.512.3	1	+	1655	1	intergenic	novelGene_141	151443710	151445364
PB.521.1	1	+	1632	1	intergenic	novelGene_142	153063614	153065245
PB.526.1	1	+	1553	1	intergenic	novelGene_143	154279926	154281478
PB.56.4	1	+	1395	1	intergenic	novelGene_14	18029067	18030461

PB.60.1	1	-	1108	1	intergenic	novelGene_15	20743336	20744443
PB.62.6	1	+	2250	1	intergenic	novelGene_16	21264300	21266549
PB.64.1	1	+	283	1	intergenic	novelGene_17	23867306	23867588
PB.69.1	1	+	1934	1	intergenic	novelGene_18	34199413	34201346
PB.70.6	1	-	1559	1	intergenic	novelGene_19	34863084	34864642
PB.73.1	1	+	3957	1	intergenic	novelGene_20	34973060	34977016
PB.78.4	1	+	1309	1	intergenic	novelGene_21	41288697	41290005
PB.86.1	1	+	1509	1	intergenic	novelGene_22	43906467	43907975
PB.87.5	1	-	3168	1	intergenic	novelGene_23	43927445	43930612
PB.94.1	1	+	1295	1	intergenic	novelGene_24	45039348	45040642
PB.96.1	1	-	1875	1	intergenic	novelGene_25	45724936	45726810
PB.99.1	1	+	2140	1	antisense	novelGene_ENSCHIG00000025095_AS	45793844	45795983
PB.99.2	1	+	2541	1	antisense	novelGene_ENSCHIG00000025095_AS	45793856	45796396
PB.4621.1	10	+	2437	1	intergenic	novelGene_977	148999	151435
PB.4626.1	10	+	1005	1	intergenic	novelGene_978	408864	409868
PB.4629.1	10	-	1844	1	intergenic	novelGene_979	1038504	1040347
PB.4630.2	10	-	1834	1	intergenic	novelGene_980	1206939	1208772
PB.4635.1	10	-	1622	1	intergenic	novelGene_981	2955445	2957066
PB.4636.1	10	+	1613	1	intergenic	novelGene_982	3026574	3028186
PB.4640.1	10	+	1956	1	intergenic	novelGene_986	5988231	5990186
PB.4641.3	10	-	2992	1	intergenic	novelGene_983	5890100	5893091
PB.4641.6	10	-	3292	1	intergenic	novelGene_985	5986030	5989321
PB.4641.7	10	-	2087	1	intergenic	novelGene_984	5986030	5988116
PB.4653.1	10	-	2769	1	intergenic	novelGene_987	14533122	14535890
PB.4660.1	10	-	1203	1	intergenic	novelGene_988	15556930	15558132
PB.4661.1	10	+	1868	1	intergenic	novelGene_989	15616653	15618520
PB.4702.1	10	+	1658	1	intergenic	novelGene_990	20131588	20133245
PB.4703.1	10	-	2074	1	intergenic	novelGene_991	20218581	20220654
PB.4711.2	10	-	2241	1	intergenic	novelGene_992	21665751	21667991
PB.4717.1	10	-	4326	1	intergenic	novelGene_993	22516549	22520874
PB.4727.7	10	-	2187	1	intergenic	novelGene_994	23902081	23904267
PB.4729.1	10	+	1760	1	intergenic	novelGene_995	23924186	23925945
PB.4745.8	10	-	2398	2	intergenic	novelGene_996	26992677	27046721
PB.4747.10	10	+	1235	1	intergenic	novelGene_997	27338307	27339541
PB.4748.1	10	-	1380	1	intergenic	novelGene_998	27368750	27370129
PB.4752.5	10	-	1254	1	intergenic	novelGene_999	29739397	29740650
PB.4761.10	10	-	1716	1	intergenic	novelGene_1000	31694418	31696133
PB.4765.1	10	+	1262	3	intergenic	novelGene_1001	32637095	32648021
PB.4765.2	10	+	1459	5	intergenic	novelGene_1002	32637103	32648022
PB.4765.3	10	+	1265	6	intergenic	novelGene_1003	32637103	32650924

PB.4765.4	10	+	1207	5	intergenic	novelGene_1004	32637103	32650924
PB.4781.5	10	-	2261	1	intergenic	novelGene_1005	36054757	36057017
PB.4781.6	10	-	1839	3	intergenic	novelGene_1006	36060785	36135995
PB.4795.1	10	-	1818	1	intergenic	novelGene_1007	38161325	38163142
PB.4804.1	10	-	2074	1	intergenic	novelGene_1008	41925800	41927873
PB.4815.1	10	+	2470	1	intergenic	novelGene_1009	43757687	43760156
PB.4826.1	10	-	406	1	intergenic	novelGene_1010	45489700	45490105
PB.4829.1	10	-	1545	2	intergenic	novelGene_1011	48186899	48189654
PB.4832.1	10	-	1920	1	intergenic	novelGene_1012	48960855	48962774
PB.4835.5	10	+	2856	1	intergenic	novelGene_1013	49841715	49844570
PB.4836.1	10	-	1957	3	intergenic	novelGene_1014	50165468	50180370
PB.4836.2	10	-	1814	3	intergenic	novelGene_1015	50165468	50180370
PB.4841.2	10	-	1540	1	intergenic	novelGene_1016	51485840	51487379
PB.4848.2	10	-	1621	1	intergenic	novelGene_1017	52538656	52540276
PB.4850.1	10	+	1461	1	intergenic	novelGene_1020	53325842	53327302
PB.4851.15	10	-	1970	1	intergenic	novelGene_1018	53307036	53309005
PB.4851.17	10	-	1553	1	intergenic	novelGene_1019	53316803	53318355
PB.4853.1	10	+	2022	1	intergenic	novelGene_1021	53468600	53470621
PB.4853.2	10	+	1665	1	intergenic	novelGene_1022	53468605	53470269
PB.4854.10	10	-	1795	1	intergenic	novelGene_1025	53528820	53530614
PB.4854.8	10	-	2501	1	intergenic	novelGene_1023	53512445	53514945
PB.4854.9	10	-	3127	1	intergenic	novelGene_1024	53520344	53523470
PB.4855.1	10	+	1772	1	intergenic	novelGene_1026	53833572	53835343
PB.4856.1	10	-	2473	1	intergenic	novelGene_1027	53871063	53873535
PB.4857.1	10	-	1380	1	intergenic	novelGene_1028	54030573	54031952
PB.4858.1	10	-	1826	1	intergenic	novelGene_1029	54060175	54062000
PB.4859.1	10	-	2408	2	intergenic	novelGene_1030	54127684	54130142
PB.4860.1	10	-	1876	1	intergenic	novelGene_1031	54131844	54133719
PB.4861.1	10	+	1756	1	intergenic	novelGene_1032	54229084	54230839
PB.4869.12	10	-	1866	1	intergenic	novelGene_1033	56406586	56408451
PB.4869.13	10	-	2070	1	intergenic	novelGene_1034	56459652	56461721
PB.4870.7	10	+	1279	1	intergenic	novelGene_1035	56635049	56636327
PB.4870.8	10	+	1303	1	intergenic	novelGene_1036	56676658	56677960
PB.4873.2	10	-	2544	1	intergenic	novelGene_1037	56915387	56917930
PB.4877.1	10	-	1199	1	intergenic	novelGene_1038	57198296	57199494
PB.4879.1	10	-	2548	1	intergenic	novelGene_1039	57245278	57247825
PB.4882.2	10	+	2264	1	intergenic	novelGene_1040	57569548	57571811
PB.4883.7	10	-	4091	1	intergenic	novelGene_1041	57679085	57683175
PB.4890.1	10	+	2618	1	intergenic	novelGene_1044	58571391	58574008
PB.4891.5	10	-	1596	1	intergenic	novelGene_1042	58567144	58568739
PB.4891.6	10	-	1796	1	intergenic	novelGene_1043	58570982	58572777
PB.4892.1	10	-	2770	4	intergenic	novelGene_1045	58596039	58752314
PB.4894.1	10	-	3091	3	intergenic	novelGene_1046	59243327	59263454
PB.4901.1	10	-	1723	2	intergenic	novelGene_1047	60120781	60123524

PB.4901.2	10	-	1622	1	intergenic	novelGene_1048	60121882	60123503
PB.4902.1	10	+	1681	1	intergenic	novelGene_1050	60179837	60181517
PB.4903.3	10	-	1297	1	intergenic	novelGene_1049	60176245	60177541
PB.4911.1	10	+	1827	1	intergenic	novelGene_1051	60400656	60402482
PB.4911.2	10	+	291	3	intergenic	novelGene_1052	60400697	60402482
PB.4915.1	10	+	1383	1	intergenic	novelGene_1053	64937991	64939373
PB.4916.1	10	-	2589	1	intergenic	novelGene_1054	65077200	65079788
PB.4938.1	10	-	4057	3	intergenic	novelGene_1055	66298175	66308709
PB.4938.3	10	-	1606	3	intergenic	novelGene_1056	66300628	66308711
PB.4938.4	10	-	1427	3	intergenic	novelGene_1057	66300807	66308711
PB.4938.5	10	-	2258	2	intergenic	novelGene_1058	66304955	66308700
PB.4944.1	10	+	1013	2	intergenic	novelGene_1059	66693812	66695699
PB.4959.2	10	-	1841	1	intergenic	novelGene_1060	67128483	67130323
PB.4967.1	10	+	1178	1	intergenic	novelGene_1061	68948689	68949866
PB.4968.2	10	-	2203	1	intergenic	novelGene_1062	69196839	69199041
PB.4981.1	10	-	2509	2	intergenic	novelGene_1063	74743606	74746684
PB.4981.2	10	-	1523	2	intergenic	novelGene_1064	74744592	74746684
PB.5003.3	10	-	1119	1	intergenic	novelGene_1065	77205772	77206890
PB.5040.2	10	-	971	6	intergenic	novelGene_1066	80481353	80484860
PB.5055.2	10	-	1271	1	intergenic	novelGene_1067	81483011	81484281
PB.5058.1	10	-	2176	1	intergenic	novelGene_1068	81934735	81936910
PB.5059.1	10	-	2148	1	intergenic	novelGene_1069	81939614	81941761
PB.5060.1	10	+	2936	1	antisense	novelGene_ENSCHIG00000003128_AS	81943163	81946098
PB.5062.1	10	+	3142	1	intergenic	novelGene_1070	82027462	82030603
PB.5062.2	10	+	993	1	intergenic	novelGene_1071	82029611	82030603
PB.5071.1	10	+	2153	2	intergenic	novelGene_1072	84618292	84629164
PB.5074.2	10	-	1702	1	intergenic	novelGene_1073	85033503	85035204
PB.5078.1	10	-	2098	2	antisense	novelGene_ENSCHIG00000011806_AS	85636949	85641488
PB.5081.1	10	-	1622	1	intergenic	novelGene_1074	86168505	86170126
PB.5082.3	10	-	2556	1	intergenic	novelGene_1075	86180731	86183286
PB.5084.1	10	+	1529	1	intergenic	novelGene_1076	86294299	86295827
PB.5088.2	10	-	2196	1	intergenic	novelGene_1077	87246226	87248421
PB.5092.1	10	+	1903	1	intergenic	novelGene_1078	87982487	87984389
PB.5094.1	10	+	1485	1	intergenic	novelGene_1079	88019167	88020651
PB.5096.1	10	-	4158	1	intergenic	novelGene_1080	88490588	88494745
PB.5120.2	10	+	1262	1	intergenic	novelGene_1081	92343378	92344639
PB.5132.4	10	+	1485	1	intergenic	novelGene_1082	95601206	95602690
PB.5143.1	10	-	2642	1	intergenic	novelGene_1083	96836975	96839616
PB.5153.1	10	-	225	1	intergenic	novelGene_1084	100259086	100259310
PB.5154.4	10	-	1980	1	intergenic	novelGene_1085	100582364	100584343

PB.5154.5	10	-	1460	1	intergenic	novelGene_1086	100587836	100589295
PB.5160.1	11	+	2181	4	intergenic	novelGene_1087	718475	858774
PB.5172.1	11	+	1546	1	antisense	novelGene_ENSCHIG00000022917_AS	2276152	2277697
PB.5173.6	11	-	1753	3	antisense	novelGene_ENSCHIG00000022318_AS	2292222	2304903
PB.5188.1	11	+	1900	1	intergenic	novelGene_1088	3710231	3712130
PB.5206.1	11	-	1451	1	intergenic	novelGene_1089	6429095	6430545
PB.5207.1	11	-	2139	1	intergenic	novelGene_1090	6499415	6501553
PB.5208.1	11	-	2639	1	antisense	novelGene_ENSCHIG00000018604_AS	6516874	6519512
PB.5209.1	11	+	2456	1	intergenic	novelGene_1091	6550005	6552460
PB.5211.4	11	+	2013	2	intergenic	novelGene_1092	6783747	6786492
PB.5211.5	11	+	2740	1	intergenic	novelGene_1093	6783753	6786492
PB.5214.1	11	+	1339	1	intergenic	novelGene_1094	7066983	7068321
PB.5219.1	11	-	1535	1	intergenic	novelGene_1096	9122658	9124192
PB.5219.2	11	-	1130	2	intergenic	novelGene_1095	9122658	9124184
PB.5224.1	11	+	1710	1	antisense	novelGene_ENSCHIG00000012393_AS	9667809	9669518
PB.5231.1	11	-	2516	1	antisense	novelGene_ENSCHIG00000007562_AS	10000788	10003303
PB.5250.3	11	+	2150	1	intergenic	novelGene_1097	11626967	11629116
PB.5250.4	11	+	3511	1	intergenic	novelGene_1099	12095743	12099253
PB.5251.1	11	-	1212	1	intergenic	novelGene_1098	11924255	11925466
PB.5254.7	11	-	2926	1	intergenic	novelGene_1100	13115303	13118228
PB.5255.1	11	+	1586	1	intergenic	novelGene_1101	13218983	13220568
PB.5261.1	11	-	1507	1	intergenic	novelGene_1102	13738805	13740311
PB.5264.4	11	-	2760	1	intergenic	novelGene_1103	14438604	14441363
PB.5271.1	11	-	1943	1	intergenic	novelGene_1104	15078783	15080725
PB.5280.1	11	-	2528	1	intergenic	novelGene_1106	19416836	19419363
PB.5280.2	11	-	1465	1	intergenic	novelGene_1105	19416836	19418300
PB.5284.1	11	-	1479	1	intergenic	novelGene_1107	19530599	19532077
PB.5285.5	11	-	505	2	intergenic	novelGene_1108	19608477	19616217
PB.5287.5	11	-	1175	1	intergenic	novelGene_1109	19971001	19972175
PB.5296.1	11	-	1140	1	intergenic	novelGene_1110	21129985	21131124
PB.5297.1	11	-	1244	1	intergenic	novelGene_1111	21437140	21438383
PB.5298.1	11	-	3141	1	intergenic	novelGene_1112	22540185	22543325
PB.5299.1	11	-	1238	1	intergenic	novelGene_1113	22598097	22599334
PB.5304.1	11	-	2870	1	intergenic	novelGene_1114	24868015	24870884

PB.5305.1	11	-	3015	1	intergenic	novelGene_1115	25022402	25025416
PB.5307.1	11	-	2273	1	intergenic	novelGene_1116	25566676	25568948
PB.5308.1	11	-	1896	1	intergenic	novelGene_1117	25719895	25721790
PB.5312.10	11	+	2564	1	intergenic	novelGene_1119	26225644	26228207
PB.5312.6	11	+	2389	1	intergenic	novelGene_1118	26222559	26224947
PB.5313.1	11	-	1226	1	intergenic	novelGene_1120	26310240	26311465
PB.5314.1	11	+	2094	1	intergenic	novelGene_1121	26318254	26320347
PB.5318.1	11	-	1880	1	antisense	novelGene_ENSCHIG00000016866_AS	28319087	28320966
PB.5318.2	11	-	1949	12	antisense	novelGene_ENSCHIG00000016866_AS	28319087	28406016
PB.5319.1	11	+	2626	1	antisense	novelGene_ENSCHIG00000015763_AS	28618348	28620973
PB.5321.5	11	+	1996	1	intergenic	novelGene_1122	28728696	28730691
PB.5321.6	11	+	1450	1	intergenic	novelGene_1123	28766956	28768405
PB.5323.1	11	-	2792	1	antisense	novelGene_ENSCHIG00000026586_AS	29099672	29102463
PB.5324.1	11	+	2541	1	antisense	novelGene_ENSCHIG00000011230_AS	29162248	29164788
PB.5331.1	11	+	1155	1	intergenic	novelGene_1124	29819017	29820171
PB.5332.2	11	+	2018	1	intergenic	novelGene_1125	30234706	30236723
PB.5338.10	11	+	2022	1	intergenic	novelGene_1127	36962521	36964542
PB.5338.5	11	+	1916	1	intergenic	novelGene_1126	36935319	36937234
PB.5345.4	11	-	1441	1	intergenic	novelGene_1128	37888857	37890297
PB.5352.1	11	-	3246	1	antisense	novelGene_ENSCHIG00000019857_AS	43224326	43227571
PB.5361.1	11	+	2255	1	antisense	novelGene_ENSCHIG00000012686_AS	44272198	44274452
PB.5370.1	11	-	1071	1	intergenic	novelGene_1129	45921633	45922703
PB.5376.1	11	-	1450	1	antisense	novelGene_ENSCHIG00000024406_AS	46325201	46326650
PB.5384.1	11	-	1896	1	intergenic	novelGene_1130	47540702	47542597
PB.5386.2	11	+	3325	1	intergenic	novelGene_1131	47606453	47609777
PB.5410.1	11	+	2502	1	antisense	novelGene_ENSCHIG00000000578_AS	49447620	49450121

PB.5412.1	11	-	462	3	intergenic	novelGene_1132	49607121	49608140
PB.5417.1	11	-	1567	1	intergenic	novelGene_1133	59688678	59690244
PB.5419.1	11	+	2050	1	intergenic	novelGene_1134	59786107	59788156
PB.5419.2	11	+	1812	1	intergenic	novelGene_1135	59786138	59787949
PB.5429.11	11	+	1697	1	intergenic	novelGene_1136	62020475	62022171
PB.5431.10	11	-	3138	1	intergenic	novelGene_1137	62296303	62299440
PB.5433.1	11	-	1653	1	intergenic	novelGene_1138	62652578	62654230
PB.5435.1	11	-	1110	1	intergenic	novelGene_1139	62875518	62876627
PB.5453.1	11	-	1638	1	intergenic	novelGene_1140	67434695	67436332
PB.5458.1	11	-	2361	1	intergenic	novelGene_1141	67968457	67970817
PB.5462.1	11	-	1695	6	intergenic	novelGene_1142	68078918	68139843
PB.5462.2	11	-	1960	1	intergenic	novelGene_1143	68114056	68116015
PB.5466.1	11	+	1954	4	intergenic	novelGene_1144	68279132	68285814
PB.5466.2	11	+	2314	3	intergenic	novelGene_1145	68279389	68283568
PB.5468.1	11	-	3191	3	intergenic	novelGene_1147	68327176	68334637
PB.5468.2	11	-	387	4	intergenic	novelGene_1146	68327176	68334582
PB.5473.5	11	-	1349	1	intergenic	novelGene_1148	69351088	69352436
PB.5478.1	11	-	2254	1	intergenic	novelGene_1149	70710306	70712559
PB.5479.20	11	-	1318	1	intergenic	novelGene_1150	70733233	70734550
PB.5480.1	11	+	3347	1	intergenic	novelGene_1151	70894300	70897646
PB.5480.2	11	+	2100	2	antisense	novelGene_ENSCHIG00000021817_AS	70894381	70906030
PB.5480.3	11	+	3438	1	intergenic	novelGene_1152	70894401	70897838
PB.5509.1	11	+	2587	1	intergenic	novelGene_1153	72323813	72326399
PB.5510.6	11	-	1848	1	intergenic	novelGene_1154	72371658	72373505
PB.5515.1	11	+	1386	1	intergenic	novelGene_1156	73106033	73107418
PB.5516.13	11	-	1986	1	intergenic	novelGene_1155	73083401	73085386
PB.5520.1	11	+	3232	1	intergenic	novelGene_1157	73780964	73784195
PB.5523.1	11	+	1070	1	intergenic	novelGene_1158	74593667	74594736
PB.5533.1	11	-	1405	2	intergenic	novelGene_1159	78106831	78109304
PB.5547.1	11	-	1360	1	intergenic	novelGene_1160	82629714	82631073
PB.5549.5	11	-	2291	1	intergenic	novelGene_1161	84823381	84825671
PB.5550.2	11	-	2113	1	intergenic	novelGene_1162	85672931	85675043
PB.5555.1	11	+	2377	1	intergenic	novelGene_1163	86530907	86533283
PB.5563.1	11	+	1709	1	intergenic	novelGene_1164	87222044	87223752
PB.5564.7	11	-	2499	1	intergenic	novelGene_1165	87234874	87237372
PB.5567.1	11	-	1632	1	intergenic	novelGene_1166	87494688	87496319
PB.5575.1	11	-	1523	1	intergenic	novelGene_1167	88165341	88166863
PB.5576.1	11	+	1696	1	intergenic	novelGene_1168	88169910	88171605
PB.5577.1	11	+	1720	1	intergenic	novelGene_1169	88173088	88174807
PB.5591.1	11	-	1319	1	intergenic	novelGene_1170	93579682	93581000
PB.5592.2	11	-	2258	1	intergenic	novelGene_1171	93602210	93604467
PB.5595.1	11	-	1506	1	intergenic	novelGene_1172	94148530	94150035

PB.5600.1	11	-	1732	1	intergenic	novelGene_1173	95374591	95376322
PB.5603.1	11	-	1946	2	intergenic	novelGene_1174	95548493	95551142
PB.5604.1	11	+	1184	1	intergenic	novelGene_1175	95554109	95555292
PB.5609.1	11	-	1053	1	antisense	novelGene_ENSCHIG00000007353_AS	95693018	95694070
PB.5612.1	11	-	1419	1	intergenic	novelGene_1176	96677636	96679054
PB.5614.1	11	+	1958	1	intergenic	novelGene_1177	97154656	97156613
PB.5615.1	11	-	3509	1	intergenic	novelGene_1178	97173100	97176608
PB.5616.1	11	+	2151	2	intergenic	novelGene_1179	97221788	97237321
PB.5632.1	11	-	1629	6	intergenic	novelGene_1180	98122538	98131674
PB.5656.4	11	-	3024	1	intergenic	novelGene_1181	98864515	98867538
PB.5656.5	11	-	2519	1	intergenic	novelGene_1182	98865007	98867525
PB.5657.2	11	+	4309	2	intergenic	novelGene_1183	98867784	98875316
PB.5667.1	11	+	1352	2	intergenic	novelGene_1184	99472577	99474335
PB.5670.1	11	+	2144	1	intergenic	novelGene_1185	99588181	99590324
PB.5675.2	11	+	2113	1	intergenic	novelGene_1186	99694370	99696482
PB.5676.10	11	-	2182	1	intergenic	novelGene_1187	99865314	99867495
PB.5676.11	11	-	2445	1	intergenic	novelGene_1188	99878024	99880468
PB.5680.2	11	+	2156	1	intergenic	novelGene_1189	100502207	100504362
PB.5680.3	11	+	1455	1	intergenic	novelGene_1190	100560347	100561801
PB.5680.8	11	+	1096	1	intergenic	novelGene_1191	100599091	100600186
PB.5688.1	11	-	2072	1	intergenic	novelGene_1192	101006763	101008834
PB.5689.1	11	+	2069	1	intergenic	novelGene_1193	101083559	101085627
PB.5689.2	11	+	2411	1	intergenic	novelGene_1194	101085143	101087553
PB.5692.1	11	+	2487	1	intergenic	novelGene_1195	101283867	101286353
PB.5700.1	11	+	1346	1	intergenic	novelGene_1196	102205439	102206784
PB.5702.1	11	-	1978	1	intergenic	novelGene_1197	102373529	102375506
PB.5703.3	11	-	3034	1	intergenic	novelGene_1198	102420334	102423367
PB.5706.7	11	-	2334	1	intergenic	novelGene_1199	102596180	102598513
PB.5719.1	11	-	3754	2	intergenic	novelGene_1200	103546838	103550681
PB.5719.2	11	-	1168	4	intergenic	novelGene_1201	103546838	103550750
PB.5719.3	11	-	2310	2	intergenic	novelGene_1202	103546845	103550681
PB.5719.4	11	-	2220	3	intergenic	novelGene_1203	103546845	103550681
PB.5719.5	11	-	3836	1	intergenic	novelGene_1204	103546846	103550681
PB.5721.1	11	+	1560	4	antisense	novelGene_ENSCHIG00000025808_AS	103655001	103660383
PB.5721.2	11	+	1948	3	antisense	novelGene_ENSCHIG00000025808_AS	103655006	103660388
PB.5726.11	11	-	1614	2	intergenic	novelGene_1205	103683579	103685400
PB.5727.2	11	-	1982	8	intergenic	novelGene_1206	103708898	103717793
PB.5733.1	11	+	3270	2	intergenic	novelGene_1207	104172741	104176064

PB.5733.2	11	+	2592	3	intergenic	novelGene_1208	104172742	104176065
PB.5734.1	11	+	1784	2	intergenic	novelGene_1209	104193815	104196226
PB.5734.2	11	+	1590	2	intergenic	novelGene_1210	104193827	104196231
PB.5737.1	11	-	1532	3	intergenic	novelGene_1211	104277504	104280332
PB.5745.1	11	-	2070	2	intergenic	novelGene_1212	105208513	105211411
PB.5746.1	11	-	4019	2	intergenic	novelGene_1213	105285437	105289644
PB.5754.1	11	+	2081	2	intergenic	novelGene_1214	105913592	105919361
PB.5797.1	12	+	1685	1	intergenic	novelGene_1215	10411977	10413661
PB.5803.14	12	-	3040	1	intergenic	novelGene_1216	11894231	11897270
PB.5803.15	12	-	2321	1	intergenic	novelGene_1217	11894950	11897270
PB.5803.16	12	-	1252	1	intergenic	novelGene_1218	11896023	11897274
PB.5808.4	12	+	992	1	genic_intron	novelGene_1219	16471462	16472453
PB.5811.1	12	-	2168	3	antisense	novelGene_ENSCHIG00000019582_AS	16854760	16858654
PB.5816.8	12	-	2664	1	intergenic	novelGene_1220	31457886	31460549
PB.5816.9	12	-	2112	1	intergenic	novelGene_1221	31461284	31463395
PB.5821.1	12	-	1347	1	intergenic	novelGene_1222	33736456	33737802
PB.5826.3	12	+	3501	1	intergenic	novelGene_1223	34026255	34029755
PB.5826.4	12	+	2442	1	intergenic	novelGene_1224	34027317	34029758
PB.5828.1	12	+	2096	1	intergenic	novelGene_1225	35799427	35801522
PB.5830.1	12	+	1889	2	antisense	novelGene_ENSCHIG00000018162_AS	35807808	35809784
PB.5832.1	12	-	2678	1	intergenic	novelGene_1226	38490539	38493216
PB.5838.1	12	-	1866	1	intergenic	novelGene_1227	39942449	39944314
PB.5842.1	12	-	2149	1	antisense	novelGene_ENSCHIG00000020165_AS	50401176	50403324
PB.5843.7	12	-	1591	1	intergenic	novelGene_1228	50457082	50458672
PB.5845.1	12	-	1269	1	intergenic	novelGene_1229	50559642	50560910
PB.5847.1	12	-	1482	2	intergenic	novelGene_1230	50674333	50679407
PB.5857.1	12	+	976	4	antisense	novelGene_ENSCHIG00000019056_AS	51221157	51243016
PB.5878.1	12	-	1189	1	intergenic	novelGene_1231	55713000	55714188
PB.5880.5	12	-	1761	1	intergenic	novelGene_1232	55878696	55880456
PB.5885.1	12	+	2250	1	antisense	novelGene_ENSCHIG00000003518_AS	56920070	56922319
PB.5890.1	12	-	1905	1	intergenic	novelGene_1233	58027267	58029171
PB.5893.7	12	-	1408	1	intergenic	novelGene_1234	58166013	58167420
PB.5895.1	12	-	1805	1	intergenic	novelGene_1235	58347797	58349601

PB.5896.2	12	-	3373	1	intergenic	novelGene_1236	58849276	58852648
PB.5911.1	12	+	2073	1	intergenic	novelGene_1238	64740660	64742732
PB.5912.1	12	+	1884	1	intergenic	novelGene_1239	64770160	64772043
PB.5913.2	12	-	1866	1	intergenic	novelGene_1237	64737629	64739494
PB.5914.2	12	-	1660	1	intergenic	novelGene_1240	64816328	64817987
PB.5922.1	12	-	2361	1	antisense	novelGene_ENSCHIG00000011286_AS	65410384	65412744
PB.5923.4	12	-	2867	1	intergenic	novelGene_1241	65532125	65534991
PB.5925.1	12	-	1515	1	intergenic	novelGene_1242	65708269	65709783
PB.5927.1	12	+	4135	1	intergenic	novelGene_1243	67096222	67100356
PB.5928.1	12	-	1979	1	intergenic	novelGene_1244	67100217	67102195
PB.5935.1	12	-	1748	1	intergenic	novelGene_1245	67554343	67556090
PB.5938.2	12	-	1696	1	intergenic	novelGene_1246	67748737	67750432
PB.5939.1	12	-	1586	1	intergenic	novelGene_1247	67858869	67860454
PB.5943.1	12	-	2042	1	intergenic	novelGene_1248	68378913	68380954
PB.5944.1	12	+	1748	1	intergenic	novelGene_1249	68518974	68520721
PB.5945.1	12	+	3098	1	intergenic	novelGene_1251	68525090	68528187
PB.5945.2	12	+	1035	1	intergenic	novelGene_1252	68527761	68528795
PB.5946.12	12	-	2719	1	intergenic	novelGene_1250	68521287	68524005
PB.5948.1	12	+	885	2	antisense	novelGene_ENSCHIG00000024171_AS	68683431	68684407
PB.5962.1	12	-	1754	1	intergenic	novelGene_1253	71749323	71751076
PB.5963.1	12	+	1738	1	intergenic	novelGene_1254	71769423	71771160
PB.5974.4	12	+	1467	1	intergenic	novelGene_1255	75296031	75297497
PB.5975.5	12	+	2536	1	intergenic	novelGene_1256	75406781	75409316
PB.5977.1	12	-	2442	1	intergenic	novelGene_1257	84753836	84756277
PB.5978.1	13	+	2169	2	intergenic	novelGene_1258	295152	306921
PB.5978.2	13	+	1219	2	intergenic	novelGene_1259	295157	305976
PB.5979.2	13	-	1455	1	intergenic	novelGene_1260	339667	341121
PB.5980.1	13	+	2293	1	intergenic	novelGene_1261	889941	892233
PB.5981.2	13	+	2001	1	intergenic	novelGene_1262	1994153	1996153
PB.5982.1	13	-	1699	1	intergenic	novelGene_1263	3179729	3181427
PB.5984.2	13	-	2385	2	intergenic	novelGene_1264	3505733	3514280
PB.5984.3	13	-	2707	1	intergenic	novelGene_1265	3511688	3514394
PB.5993.3	13	+	1990	1	intergenic	novelGene_1266	10381231	10383220
PB.6002.1	13	+	1613	1	intergenic	novelGene_1267	12174959	12176571
PB.6007.1	13	+	2449	2	intergenic	novelGene_1268	12453109	12456632
PB.6007.2	13	+	1357	2	intergenic	novelGene_1269	12453119	12455553
PB.6008.1	13	+	1360	1	intergenic	novelGene_1270	14933228	14934587
PB.6010.1	13	-	1697	1	intergenic	novelGene_1271	15386038	15387734
PB.6024.25	13	+	3285	1	intergenic	novelGene_1272	17225108	17228392
PB.6030.10	13	-	1407	1	intergenic	novelGene_1273	17555812	17557218

PB.6037.6	13	+	2264	1	intergenic	novelGene_1274	20743962	20746225
PB.6038.1	13	-	1601	1	intergenic	novelGene_1275	20775017	20776617
PB.6039.1	13	-	1546	1	intergenic	novelGene_1276	20826590	20828135
PB.6040.1	13	-	2073	1	intergenic	novelGene_1277	21065792	21067864
PB.6041.1	13	+	1569	1	intergenic	novelGene_1278	21135974	21137542
PB.6049.3	13	+	1696	1	intergenic	novelGene_1279	24469225	24470920
PB.6049.6	13	+	1816	1	intergenic	novelGene_1280	24677537	24679352
PB.6049.7	13	+	2977	1	intergenic	novelGene_1281	24695976	24698952
PB.6056.3	13	-	2738	1	intergenic	novelGene_1282	28585745	28588482
PB.6063.4	13	-	1679	1	intergenic	novelGene_1283	30467369	30469047
PB.6064.1	13	+	3501	2	antisense	novelGene_ENSCHIG00000017788_AS	30975521	30982674
PB.6075.2	13	-	2113	1	intergenic	novelGene_1284	33299894	33302006
PB.6077.1	13	-	1779	1	intergenic	novelGene_1285	34069045	34070823
PB.6081.1	13	+	3343	1	intergenic	novelGene_1286	34686093	34689435
PB.6085.1	13	-	2181	1	intergenic	novelGene_1287	35885593	35887773
PB.6088.2	13	+	2319	1	intergenic	novelGene_1288	37403964	37406282
PB.6101.1	13	-	1811	1	antisense	novelGene_ENSCHIG00000021922_AS	38959918	38961728
PB.6104.4	13	+	1758	1	intergenic	novelGene_1289	39992236	39993993
PB.6105.1	13	+	2970	1	antisense	novelGene_ENSCHIG00000010975_AS	41211737	41214706
PB.6106.6	13	-	1469	1	intergenic	novelGene_1290	41208911	41210379
PB.6134.1	13	+	1924	1	intergenic	novelGene_1291	45979795	45981718
PB.6135.1	13	+	2117	3	intergenic	novelGene_1292	46233807	46236071
PB.6137.1	13	+	2014	1	intergenic	novelGene_1293	46453113	46455126
PB.6137.7	13	+	2803	2	intergenic	novelGene_1294	46453120	46458353
PB.6138.1	13	-	3519	1	intergenic	novelGene_1296	46540037	46543555
PB.6138.2	13	-	1986	1	intergenic	novelGene_1295	46540037	46542022
PB.6143.8	13	-	1384	1	intergenic	novelGene_1297	47280967	47282350
PB.6149.2	13	+	1572	1	intergenic	novelGene_1298	50538991	50540562
PB.6155.1	13	-	1887	1	intergenic	novelGene_1299	50784250	50786136
PB.6157.3	13	-	4029	1	intergenic	novelGene_1300	50812769	50816797
PB.6160.1	13	-	2040	1	antisense	novelGene_ENSCHIG00000024722_AS	50883057	50885096
PB.6161.1	13	-	3111	1	intergenic	novelGene_1301	50894667	50897777
PB.6168.15	13	-	1355	2	intergenic	novelGene_1302	51628607	51652650
PB.6186.1	13	+	2064	1	intergenic	novelGene_1303	53225686	53227749
PB.6216.1	13	+	389	3	intergenic	novelGene_1304	56669838	56673349
PB.6225.1	13	+	1851	1	intergenic	novelGene_1305	57348075	57349925

PB.6227.1	13	+	2532	1	intergenic	novelGene_1306	57833409	57835940
PB.6231.1	13	-	2551	2	intergenic	novelGene_1307	58376019	58395623
PB.6240.1	13	-	1581	1	intergenic	novelGene_1308	59047958	59049538
PB.6241.1	13	-	1886	1	intergenic	novelGene_1309	59059796	59061681
PB.6242.2	13	+	2474	1	intergenic	novelGene_1310	59093324	59095797
PB.6257.5	13	+	2302	1	intergenic	novelGene_1311	60637402	60639703
PB.6266.1	13	+	3059	1	genic_intron	novelGene_1312	61110247	61113305
PB.6267.1	13	-	1380	1	intergenic	novelGene_1313	61165705	61167084
PB.6279.4	13	-	2941	1	intergenic	novelGene_1314	63079382	63082322
PB.6287.1	13	-	1660	1	intergenic	novelGene_1315	63675505	63677164
PB.6294.1	13	-	1616	2	intergenic	novelGene_1316	64115930	64117628
PB.6299.14	13	-	2342	1	intergenic	novelGene_1317	64431550	64433891
PB.6305.1	13	-	5189	1	intergenic	novelGene_1319	64754862	64760050
PB.6305.2	13	-	2570	1	intergenic	novelGene_1318	64754862	64757431
PB.6305.3	13	-	1523	1	intergenic	novelGene_1320	64754863	64756385
PB.6305.4	13	-	1669	1	intergenic	novelGene_1321	64758400	64760068
PB.6305.5	13	-	883	1	intergenic	novelGene_1322	64759168	64760050
PB.6305.6	13	-	824	2	intergenic	novelGene_1323	64759168	64760050
PB.6319.6	13	-	2119	1	intergenic	novelGene_1324	66029265	66031383
PB.6329.1	13	-	3203	3	intergenic	novelGene_1325	66913617	66921956
PB.6329.3	13	-	2354	3	intergenic	novelGene_1326	66914418	66921908
PB.6330.1	13	+	2590	4	antisense	novelGene_ENSCHIG00000002222_AS	66934388	66940575
PB.6331.1	13	+	2163	1	intergenic	novelGene_1327	67045846	67048008
PB.6336.1	13	-	2205	1	intergenic	novelGene_1328	69402829	69405033
PB.6339.1	13	-	2091	1	intergenic	novelGene_1329	71656861	71658951
PB.6341.2	13	+	1190	1	intergenic	novelGene_1330	71770919	71772108
PB.6343.1	13	+	2053	1	intergenic	novelGene_1331	72216412	72218464
PB.6350.8	13	+	1486	1	intergenic	novelGene_1332	72822832	72824317
PB.6353.1	13	+	1793	1	intergenic	novelGene_1333	72990994	72992786
PB.6354.1	13	+	3088	3	intergenic	novelGene_1334	73014954	73022843
PB.6361.2	13	+	1253	13	intergenic	novelGene_1335	74069016	74093940
PB.6371.1	13	-	1052	1	intergenic	novelGene_1336	74194770	74195821
PB.6378.1	13	+	2338	1	antisense	novelGene_ENSCHIG00000026217_AS	74888065	74890402
PB.6378.2	13	+	2132	1	antisense	novelGene_ENSCHIG00000026217_AS	74888077	74890208
PB.6383.3	13	-	2998	1	intergenic	novelGene_1337	76434668	76437665
PB.6383.4	13	-	2298	1	intergenic	novelGene_1338	76526621	76528918
PB.6385.1	13	+	1904	1	intergenic	novelGene_1339	76710879	76712782

PB.6391.4	13	-	1685	1	intergenic	novelGene_1340	77177903	77179587
PB.6392.1	13	-	2370	2	intergenic	novelGene_1341	77222269	77226630
PB.6409.1	13	+	2168	1	intergenic	novelGene_1342	80134814	80136981
PB.6415.1	14	-	1532	1	intergenic	novelGene_1343	342215	343746
PB.6417.1	14	+	1885	2	intergenic	novelGene_1344	479401	486389
PB.6422.9	14	+	1500	1	intergenic	novelGene_1346	1123868	1125367
PB.6423.1	14	-	1213	4	intergenic	novelGene_1345	1112158	1114801
PB.6424.1	14	+	1702	1	intergenic	novelGene_1347	1242712	1244413
PB.6427.2	14	+	1680	1	intergenic	novelGene_1348	1360126	1361805
PB.6428.1	14	-	2592	2	intergenic	novelGene_1349	1411426	1414962
PB.6435.1	14	-	1939	3	intergenic	novelGene_1350	5716023	5723063
PB.6444.1	14	-	2045	2	intergenic	novelGene_1351	8885527	8887681
PB.6444.2	14	-	1874	2	intergenic	novelGene_1352	8885696	8887679
PB.6446.1	14	+	3119	1	intergenic	novelGene_1353	9904529	9907647
PB.6449.3	14	-	3616	1	intergenic	novelGene_1354	11670976	11674591
PB.6449.4	14	-	2795	1	intergenic	novelGene_1355	11671812	11674606
PB.6454.1	14	-	1183	1	intergenic	novelGene_1356	12580247	12581429
PB.6470.1	14	+	1439	1	intergenic	novelGene_1357	15717433	15718871
PB.6476.1	14	+	2033	1	intergenic	novelGene_1358	16223789	16225821
PB.6480.2	14	+	3156	21	intergenic	novelGene_1359	16964384	17287535
PB.6481.1	14	+	2684	7	intergenic	novelGene_1360	17728388	17750235
PB.6484.1	14	+	937	4	intergenic	novelGene_1361	17980626	17983336
PB.6484.2	14	+	1027	4	intergenic	novelGene_1362	17980633	17983315
PB.6485.5	14	-	1140	1	intergenic	novelGene_1363	18097465	18098604
PB.6493.3	14	-	1577	1	intergenic	novelGene_1364	20190240	20191816
PB.6495.1	14	-	1798	1	intergenic	novelGene_1365	20510026	20511823
PB.6496.11	14	-	1350	1	intergenic	novelGene_1366	20584358	20585707
PB.6504.1	14	+	1436	1	intergenic	novelGene_1367	23222327	23223762
PB.6513.1	14	-	1668	1	intergenic	novelGene_1368	33152804	33154471
PB.6514.1	14	+	1558	1	intergenic	novelGene_1371	33249626	33251183
PB.6515.1	14	+	1659	1	intergenic	novelGene_1373	33282691	33284349
PB.6516.2	14	-	1578	1	intergenic	novelGene_1369	33184083	33185660
PB.6516.3	14	-	1856	1	intergenic	novelGene_1370	33238012	33239867
PB.6516.4	14	-	1505	1	intergenic	novelGene_1372	33280940	33282444
PB.6516.5	14	-	1860	1	intergenic	novelGene_1374	33299224	33301083
PB.6516.6	14	-	2241	1	intergenic	novelGene_1375	33315809	33318049
PB.6519.4	14	-	1223	1	intergenic	novelGene_1376	34628285	34629507
PB.6522.1	14	-	2157	1	intergenic	novelGene_1377	35784792	35786948
PB.6523.1	14	-	2939	1	intergenic	novelGene_1379	35800146	35803084
PB.6523.2	14	-	1468	1	intergenic	novelGene_1378	35800146	35801613
PB.6524.1	14	+	2927	1	intergenic	novelGene_1380	35809991	35812917
PB.6525.1	14	-	3039	1	intergenic	novelGene_1381	35831378	35834416
PB.6526.1	14	+	1087	1	intergenic	novelGene_1382	35843338	35844424
PB.6527.1	14	-	3407	1	intergenic	novelGene_1384	35860088	35863494

PB.6527.2	14	-	1514	1	intergenic	novelGene_1383	35860088	35861601
PB.6528.1	14	-	1282	1	intergenic	novelGene_1385	35882188	35883469
PB.6529.1	14	+	1526	1	intergenic	novelGene_1386	36448725	36450250
PB.6534.1	14	+	2803	3	antisense	novelGene_ENSCHIG00000022058_AS	37561231	37578550
PB.6536.2	14	+	2145	1	intergenic	novelGene_1387	37845970	37848114
PB.6537.4	14	+	1912	1	intergenic	novelGene_1388	38710753	38712664
PB.6542.1	14	-	2024	1	antisense	novelGene_ENSCHIG00000013214_AS	40045043	40047066
PB.6552.1	14	+	1716	1	intergenic	novelGene_1389	45482181	45483896
PB.6567.3	14	-	2248	1	intergenic	novelGene_1390	50868572	50870819
PB.6579.1	14	-	1540	1	intergenic	novelGene_1391	54058285	54059824
PB.6582.1	14	-	2185	1	intergenic	novelGene_1392	55673650	55675834
PB.6583.1	14	+	1593	1	intergenic	novelGene_1393	55699618	55701210
PB.6587.5	14	-	1276	1	intergenic	novelGene_1394	57336822	57338097
PB.6597.1	14	+	1726	1	intergenic	novelGene_1395	59008804	59010529
PB.6603.1	14	-	1488	1	intergenic	novelGene_1396	60526108	60527595
PB.6607.1	14	-	2757	1	antisense	novelGene_ENSCHIG00000024410_AS	62097998	62100754
PB.6610.5	14	+	858	1	intergenic	novelGene_1398	62828106	62828963
PB.6611.1	14	-	1453	1	intergenic	novelGene_1397	62656273	62657725
PB.6614.3	14	+	2124	1	intergenic	novelGene_1399	65111178	65113301
PB.6614.4	14	+	2323	1	intergenic	novelGene_1400	65172672	65174994
PB.6625.1	14	+	3035	1	intergenic	novelGene_1401	66347061	66350095
PB.6630.6	14	-	2353	1	intergenic	novelGene_1402	66525356	66527708
PB.6636.1	14	-	1580	1	intergenic	novelGene_1403	67368402	67369981
PB.6638.1	14	-	1668	1	intergenic	novelGene_1404	69407445	69409112
PB.6639.1	14	+	1900	1	intergenic	novelGene_1405	71264578	71266477
PB.6645.7	14	-	2090	1	intergenic	novelGene_1406	73697505	73699594
PB.6648.2	14	-	2450	1	intergenic	novelGene_1407	74018135	74020584
PB.6648.4	14	-	3268	1	intergenic	novelGene_1408	74105821	74109088
PB.6648.5	14	-	2790	1	intergenic	novelGene_1409	74106299	74109088
PB.6652.1	14	-	2965	1	intergenic	novelGene_1410	78983623	78986587
PB.6660.1	14	-	1462	2	intergenic	novelGene_1411	80417004	80418649
PB.6660.2	14	-	677	3	intergenic	novelGene_1412	80417004	80418649
PB.6663.1	14	+	1752	3	intergenic	novelGene_1413	80735146	80740938
PB.6673.1	14	-	2145	4	intergenic	novelGene_1415	80956077	80960590
PB.6673.2	14	-	2064	4	intergenic	novelGene_1414	80956077	80960558
PB.6674.3	14	+	2510	1	intergenic	novelGene_1416	80967561	80970070
PB.6679.1	14	+	2931	2	intergenic	novelGene_1417	81178702	81182142
PB.6681.1	14	-	2268	4	antisense	novelGene_ENSC	81180803	81189439

						HIG00000021121_AS		
PB.6716.1	14	+	1815	1	intergenic	novelGene_1418	93693700	93695514
PB.6717.1	14	+	1254	1	intergenic	novelGene_1419	93701180	93702433
PB.6721.1	15	+	1741	2	intergenic	novelGene_1420	348066	349991
PB.6728.1	15	-	2030	1	intergenic	novelGene_1421	1216278	1218307
PB.6729.6	15	+	1421	1	intergenic	novelGene_1422	1267311	1268731
PB.6730.1	15	-	3246	1	intergenic	novelGene_1423	1451827	1455072
PB.6730.2	15	-	1739	1	intergenic	novelGene_1424	1451828	1453566
PB.6732.1	15	+	2085	1	antisense	novelGene_ENSCHIG00000024436_AS	2275315	2277399
PB.6742.1	15	-	1744	2	intergenic	novelGene_1425	2447818	2456912
PB.6748.17	15	+	1903	1	intergenic	novelGene_1426	4021941	4023843
PB.6749.2	15	-	2366	1	intergenic	novelGene_1427	5253873	5256238
PB.6749.3	15	-	3280	1	intergenic	novelGene_1428	5261930	5265209
PB.6772.9	15	-	2753	1	intergenic	novelGene_1429	6404523	6407275
PB.6774.2	15	+	2394	2	intergenic	novelGene_1430	6424813	6427737
PB.6775.1	15	-	1762	1	intergenic	novelGene_1431	6503080	6504841
PB.6785.5	15	-	2734	1	intergenic	novelGene_1432	8016571	8019304
PB.6808.3	15	-	1625	1	intergenic	novelGene_1433	18224067	18225691
PB.6814.3	15	+	2278	1	intergenic	novelGene_1434	19175209	19177486
PB.6824.1	15	+	1579	1	intergenic	novelGene_1435	24815549	24817127
PB.6825.2	15	+	1044	1	intergenic	novelGene_1436	24859572	24860615
PB.6833.1	15	+	1738	1	intergenic	novelGene_1437	26967652	26969389
PB.6836.1	15	+	975	1	intergenic	novelGene_1438	27602744	27603718
PB.6840.1	15	-	2423	1	intergenic	novelGene_1439	27917375	27919797
PB.6846.1	15	-	3211	1	intergenic	novelGene_1440	28550067	28553277
PB.6854.1	15	+	3547	1	intergenic	novelGene_1441	29447083	29450629
PB.6861.1	15	-	2218	2	antisense	novelGene_ENSCHIG00000023957_AS	29784886	29787190
PB.6892.1	15	-	1507	3	intergenic	novelGene_1443	35558010	35561341
PB.6892.2	15	-	1355	3	intergenic	novelGene_1442	35558010	35561315
PB.6905.6	15	-	3586	3	intergenic	novelGene_1444	36172370	36266628
PB.6909.1	15	-	1702	1	intergenic	novelGene_1445	37324248	37325949
PB.6916.1	15	+	1727	1	intergenic	novelGene_1446	38759312	38761038
PB.6919.1	15	-	2593	1	intergenic	novelGene_1447	39009981	39012573
PB.6922.1	15	-	2065	1	intergenic	novelGene_1448	39196053	39198117
PB.6923.1	15	+	3122	1	intergenic	novelGene_1449	39201565	39204686
PB.6929.1	15	-	2328	1	intergenic	novelGene_1450	40273057	40275384
PB.6938.1	15	-	3731	1	intergenic	novelGene_1451	42341385	42345115
PB.6940.1	15	+	1845	1	intergenic	novelGene_1452	42932794	42934638
PB.6941.4	15	-	2048	1	intergenic	novelGene_1453	43118571	43120618

PB.6944.1	15	+	2487	1	intergenic	novelGene_1454	44085060	44087546
PB.6949.1	15	+	1741	1	intergenic	novelGene_1455	46720940	46722680
PB.6950.1	15	+	1474	4	intergenic	novelGene_1456	46884680	46911258
PB.6960.1	15	-	2510	1	intergenic	novelGene_1457	48648442	48650951
PB.6967.1	15	+	1908	1	intergenic	novelGene_1458	51434532	51436439
PB.6971.1	15	-	2187	1	antisense	novelGene_ENSCHIG00000025539_AS	51650833	51653019
PB.7012.1	15	+	2067	3	antisense	novelGene_ENSCHIG00000014004_AS	54457253	54461465
PB.7016.1	15	+	2089	1	intergenic	novelGene_1459	54589180	54591268
PB.7017.1	15	+	2914	1	intergenic	novelGene_1460	54661980	54664893
PB.7018.1	15	-	2346	1	intergenic	novelGene_1461	54664689	54667034
PB.7024.1	15	+	2932	2	intergenic	novelGene_1462	56277189	56390694
PB.7025.1	15	-	1876	1	intergenic	novelGene_1463	56422025	56423900
PB.7026.1	15	-	1900	1	intergenic	novelGene_1464	56428025	56429924
PB.7029.1	15	+	1776	1	intergenic	novelGene_1465	57609478	57611253
PB.7043.1	15	-	2136	1	intergenic	novelGene_1466	60317695	60319830
PB.7050.1	15	+	3602	1	intergenic	novelGene_1467	64132994	64136595
PB.7055.1	15	+	3975	1	intergenic	novelGene_1468	64741449	64745423
PB.7058.1	15	+	678	2	intergenic	novelGene_1469	65110657	65115174
PB.7059.3	15	-	2837	1	antisense	novelGene_ENSCHIG00000002085_AS	65275802	65278638
PB.7065.1	15	+	2441	1	intergenic	novelGene_1470	67070028	67072468
PB.7066.1	15	-	2416	1	intergenic	novelGene_1471	67090459	67092874
PB.7068.1	15	-	2622	1	intergenic	novelGene_1472	67154968	67157589
PB.7073.1	15	-	1507	1	intergenic	novelGene_1473	68332255	68333761
PB.7074.1	15	+	1925	1	intergenic	novelGene_1474	68367935	68369859
PB.7075.1	15	-	2525	1	intergenic	novelGene_1475	68375648	68378172
PB.7076.1	15	-	1701	1	intergenic	novelGene_1476	68409969	68411669
PB.7077.1	15	-	3265	1	intergenic	novelGene_1477	68434524	68437788
PB.7078.1	15	-	2149	1	intergenic	novelGene_1478	68452127	68454275
PB.7081.1	15	+	1878	1	intergenic	novelGene_1479	71966570	71968447
PB.7081.2	15	+	2658	1	intergenic	novelGene_1480	71966631	71969288
PB.7084.1	15	+	2192	2	antisense	novelGene_ENSCHIG00000002119_AS	75922802	75927899
PB.7085.1	15	+	2100	1	intergenic	novelGene_1481	75951208	75953307
PB.7085.2	15	+	2509	1	intergenic	novelGene_1482	75951470	75953978
PB.7086.1	15	-	1373	1	intergenic	novelGene_1483	75955864	75957236
PB.7087.1	15	+	2416	3	intergenic	novelGene_1484	76056102	76061459
PB.7087.2	15	+	2311	2	intergenic	novelGene_1485	76056102	76061459

PB.7088.1	15	+	1274	2	intergenic	novelGene_1486	76104680	76107287
PB.7088.3	15	+	2553	1	intergenic	novelGene_1487	76104734	76107286
PB.7089.18	15	-	2792	1	intergenic	novelGene_1489	76241082	76243873
PB.7089.4	15	-	1280	1	intergenic	novelGene_1488	76158812	76160091
PB.7097.3	15	-	2108	1	intergenic	novelGene_1490	77902739	77904846
PB.7100.1	15	-	2271	2	intergenic	novelGene_1491	79282560	79295133
PB.7101.3	15	-	1866	1	intergenic	novelGene_1492	80757851	80759716
PB.7109.1	16	+	2579	2	intergenic	novelGene_1493	456609	462239
PB.7110.1	16	+	1915	1	intergenic	novelGene_1495	476350	478264
PB.7111.1	16	-	3084	1	intergenic	novelGene_1494	476269	479352
PB.7114.1	16	+	2650	3	antisense	novelGene_ENSCHIG00000019212_AS	915667	932537
PB.7116.1	16	-	2361	1	antisense	novelGene_ENSCHIG00000011089_AS	950569	952929
PB.7119.1	16	+	2639	2	intergenic	novelGene_1496	1541078	1546206
PB.7119.2	16	+	2579	2	intergenic	novelGene_1497	1541118	1546206
PB.7119.3	16	+	2781	2	intergenic	novelGene_1498	1541667	1546205
PB.7120.1	16	-	2465	1	intergenic	novelGene_1499	1682220	1684684
PB.7121.1	16	+	3132	1	intergenic	novelGene_1500	1684707	1687838
PB.7124.1	16	-	1661	1	intergenic	novelGene_1501	2332499	2334159
PB.7127.1	16	-	2022	1	intergenic	novelGene_1502	2775926	2777947
PB.7129.1	16	-	3744	1	intergenic	novelGene_1504	2862033	2865776
PB.7129.2	16	-	2329	1	intergenic	novelGene_1503	2862033	2864361
PB.7140.1	16	-	1737	2	antisense	novelGene_ENSCHIG00000020279_AS	3707874	3712433
PB.7143.1	16	+	1746	2	antisense	novelGene_ENSCHIG00000016979_AS	3914572	3916555
PB.7146.1	16	-	2988	8	intergenic	novelGene_1505	4049150	4069794
PB.7148.10	16	-	1462	1	intergenic	novelGene_1506	4197233	4198694
PB.7149.3	16	-	2917	1	intergenic	novelGene_1508	4223504	4226420
PB.7149.4	16	-	1821	1	intergenic	novelGene_1507	4223504	4225324
PB.7149.5	16	-	1205	1	intergenic	novelGene_1509	4225175	4226379
PB.7150.1	16	+	1700	1	intergenic	novelGene_1510	4254863	4256562
PB.7151.1	16	+	1460	4	intergenic	novelGene_1512	4474970	4482752
PB.7151.2	16	+	1348	4	intergenic	novelGene_1511	4474970	4482640
PB.7159.1	16	-	3325	1	intergenic	novelGene_1513	10748278	10751602
PB.7191.1	16	+	1818	1	antisense	novelGene_ENSCHIG00000020527_AS	26059095	26060912
PB.7194.1	16	-	2222	1	intergenic	novelGene_1514	27095882	27098103

PB.7195.3	16	-	1589	1	intergenic	novelGene_1515	27109368	27110956
PB.7202.1	16	+	2811	1	intergenic	novelGene_1516	27741567	27744377
PB.7202.2	16	+	1140	1	intergenic	novelGene_1517	27743238	27744377
PB.7209.1	16	-	1687	1	intergenic	novelGene_1518	29253928	29255614
PB.7210.1	16	-	2222	1	intergenic	novelGene_1519	29258400	29260621
PB.7213.1	16	-	2242	1	intergenic	novelGene_1520	29846551	29848792
PB.7215.1	16	-	2792	1	antisense	novelGene_ENSCHIG00000004900_AS	30942093	30944884
PB.7215.2	16	-	1992	1	antisense	novelGene_ENSCHIG00000004900_AS	30942920	30944911
PB.7217.1	16	+	1423	1	intergenic	novelGene_1521	31098048	31099470
PB.7221.1	16	-	1768	1	antisense	novelGene_ENSCHIG00000026612_AS	32479529	32481296
PB.7238.1	16	+	1499	1	intergenic	novelGene_1522	36818251	36819749
PB.7246.4	16	+	1912	1	intergenic	novelGene_1523	38530880	38532791
PB.7249.1	16	-	2049	1	intergenic	novelGene_1524	39705145	39707193
PB.7252.1	16	-	1983	5	intergenic	novelGene_1525	40049375	40066756
PB.7269.1	16	+	3063	4	intergenic	novelGene_1526	41300103	41401529
PB.7269.2	16	+	1508	1	intergenic	novelGene_1527	41323852	41325359
PB.7273.1	16	-	2950	1	intergenic	novelGene_1528	41686947	41689896
PB.7273.2	16	-	1456	1	intergenic	novelGene_1529	41686948	41688403
PB.7274.4	16	-	1684	1	intergenic	novelGene_1530	41752747	41754430
PB.7274.5	16	-	3299	1	intergenic	novelGene_1531	41752748	41756046
PB.7275.3	16	-	1608	1	intergenic	novelGene_1532	41996257	41997864
PB.7284.1	16	-	2414	1	intergenic	novelGene_1533	42698681	42701094
PB.7288.1	16	-	1933	1	intergenic	novelGene_1534	43172578	43174510
PB.7289.1	16	-	1732	1	intergenic	novelGene_1535	43325187	43326918
PB.7290.1	16	-	1221	1	intergenic	novelGene_1536	43534668	43535888
PB.7303.2	16	-	1352	1	intergenic	novelGene_1537	45448139	45449490
PB.7323.3	16	+	1841	1	intergenic	novelGene_1538	49353972	49355812
PB.7335.1	16	-	1726	2	antisense	novelGene_ENSCHIG00000019097_AS	49643431	49647148
PB.7358.4	16	+	2399	1	intergenic	novelGene_1539	50111942	50114340
PB.7365.1	16	+	2769	1	intergenic	novelGene_1540	50367668	50370436
PB.7371.1	16	+	2238	1	intergenic	novelGene_1541	50969011	50971248
PB.7372.1	16	+	1201	1	intergenic	novelGene_1542	52308549	52309749
PB.7377.1	16	+	1768	1	intergenic	novelGene_1543	53618408	53620175
PB.7380.1	16	-	3086	3	intergenic	novelGene_1544	53756026	53759813
PB.7380.2	16	-	921	10	intergenic	novelGene_1545	53756031	53759812
PB.7381.1	16	+	1995	1	intergenic	novelGene_1546	53810917	53812911

PB.7381.2	16	+	2269	1	intergenic	novelGene_1547	53812861	53815129
PB.7383.6	16	+	1646	1	intergenic	novelGene_1548	54278741	54280386
PB.7394.1	16	+	1282	1	intergenic	novelGene_1549	59091929	59093210
PB.7395.1	16	-	1341	1	intergenic	novelGene_1550	59130752	59132092
PB.7412.1	16	-	1897	1	intergenic	novelGene_1551	62676941	62678837
PB.7422.7	16	+	2072	1	intergenic	novelGene_1552	64080880	64082951
PB.7422.8	16	+	1854	1	intergenic	novelGene_1553	64115532	64117385
PB.7428.12	16	-	3840	1	intergenic	novelGene_1554	64906766	64910605
PB.7428.13	16	-	1156	1	intergenic	novelGene_1555	64909337	64910492
PB.7429.1	16	-	1788	3	intergenic	novelGene_1556	65005460	65013345
PB.7433.3	16	-	1533	1	intergenic	novelGene_1557	66345155	66346687
PB.7436.2	16	+	2627	1	intergenic	novelGene_1558	67675386	67678012
PB.7443.13	16	-	1488	1	intergenic	novelGene_1559	69589100	69590587
PB.7461.6	16	-	1618	1	intergenic	novelGene_1560	72255766	72257383
PB.7464.1	16	-	3050	1	intergenic	novelGene_1561	72527272	72530321
PB.7464.2	16	-	3315	1	intergenic	novelGene_1562	72530096	72533410
PB.7465.1	16	+	1991	1	intergenic	novelGene_1563	73784198	73786188
PB.7468.1	16	+	1786	2	antisense	novelGene_ENSCHIG00000009909_AS	74675018	74676955
PB.7470.2	16	-	1854	1	intergenic	novelGene_1564	75217056	75218909
PB.7471.5	16	+	1435	1	intergenic	novelGene_1565	75768987	75770421
PB.7472.1	16	-	2350	1	antisense	novelGene_ENSCHIG00000014879_AS	75758525	75760874
PB.7474.1	16	+	1772	1	intergenic	novelGene_1566	76275013	76276784
PB.7475.1	16	-	2293	2	intergenic	novelGene_1567	76368682	76414158
PB.7478.1	16	+	2511	1	intergenic	novelGene_1568	77995988	77998498
PB.7488.1	16	-	2024	1	intergenic	novelGene_1569	78618805	78620828
PB.7488.2	16	-	3272	1	intergenic	novelGene_1570	78618806	78622077
PB.7494.1	16	+	4042	2	intergenic	novelGene_1571	79280488	79286651
PB.7494.2	16	+	3200	2	intergenic	novelGene_1572	79280489	79286657
PB.7499.1	17	+	2432	1	intergenic	novelGene_1573	69885	72316
PB.7504.1	17	+	3216	2	antisense	novelGene_ENSCHIG00000016094_AS	832287	839866
PB.7512.1	17	-	2689	2	antisense	novelGene_ENSCHIG00000009005_AS	1215591	1220640
PB.7513.1	17	+	2528	2	antisense	novelGene_ENSCHIG00000018019_AS	1252060	1254723
PB.7513.2	17	+	2594	1	intergenic	novelGene_1574	1252130	1254723
PB.7528.1	17	+	1825	1	antisense	novelGene_ENSC	1844869	1846693

						HIG00000014172_AS		
PB.7532.3	17	+	1905	9	intergenic	novelGene_1575	1930390	1959035
PB.7537.1	17	-	437	2	intergenic	novelGene_1576	2368967	2371025
PB.7545.1	17	-	2343	1	intergenic	novelGene_1577	2956421	2958763
PB.7550.1	17	+	1629	1	intergenic	novelGene_1578	3431278	3432906
PB.7559.1	17	+	2198	1	intergenic	novelGene_1579	5906994	5909191
PB.7566.6	17	-	1663	1	intergenic	novelGene_1580	6788578	6790240
PB.7567.1	17	+	1704	1	intergenic	novelGene_1581	6857829	6859532
PB.7569.1	17	-	1749	1	intergenic	novelGene_1582	6918980	6920728
PB.7580.1	17	+	1001	1	intergenic	novelGene_1583	7817080	7818080
PB.7587.3	17	-	2794	1	intergenic	novelGene_1584	8161157	8163950
PB.7588.3	17	-	1679	1	intergenic	novelGene_1585	8179014	8180692
PB.7603.3	17	+	4253	1	intergenic	novelGene_1586	8828963	8833215
PB.7614.1	17	-	1211	1	intergenic	novelGene_1587	9926756	9927966
PB.7621.1	17	-	2005	1	intergenic	novelGene_1588	12488081	12490085
PB.7622.1	17	+	2179	1	intergenic	novelGene_1589	12532355	12534533
PB.7636.1	17	+	1699	6	intergenic	novelGene_1590	15847522	15857245
PB.7642.8	17	+	3334	1	intergenic	novelGene_1591	16648923	16652256
PB.7648.1	17	-	1673	2	intergenic	novelGene_1592	16894172	16897121
PB.7650.1	17	+	1638	1	intergenic	novelGene_1593	16909015	16910652
PB.7665.1	17	+	2292	1	intergenic	novelGene_1594	17999883	18002174
PB.7673.1	17	+	1231	5	intergenic	novelGene_1595	18478137	18494695
PB.7680.1	17	-	2439	1	intergenic	novelGene_1596	18662980	18665418
PB.7681.1	17	+	1393	3	intergenic	novelGene_1597	18882226	18891614
PB.7689.1	17	+	2113	1	intergenic	novelGene_1598	19166126	19168238
PB.7692.1	17	+	4206	5	antisense	novelGene_ENSCHIG00000013573_AS	19466943	19502865
PB.7692.2	17	+	4193	4	intergenic	novelGene_1599	19467451	19502871
PB.7692.3	17	+	4266	5	intergenic	novelGene_1600	19467466	19502871
PB.7700.1	17	+	1079	7	intergenic	novelGene_1601	24902425	24906031
PB.7700.2	17	+	2112	7	intergenic	novelGene_1602	24902438	24907077
PB.7700.3	17	+	2526	7	intergenic	novelGene_1603	24902450	24907503
PB.7700.4	17	+	2359	7	intergenic	novelGene_1604	24902453	24907339
PB.7700.5	17	+	1174	7	intergenic	novelGene_1605	24902500	24906031
PB.7706.2	17	+	2265	1	intergenic	novelGene_1606	26167525	26169789
PB.7719.1	17	+	2885	1	intergenic	novelGene_1607	28845983	28848867
PB.7725.2	17	+	1832	1	intergenic	novelGene_1608	30874012	30875843
PB.7726.1	17	+	1937	1	intergenic	novelGene_1609	30959754	30961690
PB.7727.1	17	+	1502	1	intergenic	novelGene_1610	31172392	31173893
PB.7728.1	17	+	2393	1	intergenic	novelGene_1611	31219460	31221852
PB.7729.1	17	+	1886	1	intergenic	novelGene_1612	31237209	31239094
PB.7730.1	17	-	1422	1	intergenic	novelGene_1613	31285324	31286745

PB.7731.1	17	-	1705	1	intergenic	novelGene_1614	31379975	31381679
PB.7740.1	17	+	1897	1	antisense	novelGene_ENSCHIG00000024238_AS	41028383	41030279
PB.7740.2	17	+	1593	1	antisense	novelGene_ENSCHIG00000024238_AS	41028395	41029987
PB.7748.6	17	-	1558	1	intergenic	novelGene_1615	52447919	52449476
PB.7748.7	17	-	1380	1	intergenic	novelGene_1616	52456176	52457555
PB.7751.1	17	-	3290	1	antisense	novelGene_ENSCHIG00000022044_AS	52623476	52626765
PB.7752.1	17	-	2904	3	intergenic	novelGene_1617	52734251	52737815
PB.7752.2	17	-	1898	3	intergenic	novelGene_1618	52735301	52737845
PB.7754.1	17	-	1646	1	intergenic	novelGene_1619	52790918	52792563
PB.7754.2	17	-	3681	1	intergenic	novelGene_1621	52790931	52794611
PB.7754.3	17	-	2593	1	intergenic	novelGene_1620	52790931	52793523
PB.7762.1	17	-	1665	1	intergenic	novelGene_1622	55121284	55122948
PB.7764.1	17	-	1687	1	intergenic	novelGene_1623	56274440	56276126
PB.7767.1	17	-	1093	1	intergenic	novelGene_1624	56544484	56545576
PB.7773.3	17	+	1948	1	intergenic	novelGene_1625	60248841	60250788
PB.7773.4	17	+	1653	1	intergenic	novelGene_1626	60253351	60255003
PB.7776.7	17	+	2682	1	intergenic	novelGene_1627	60762367	60765048
PB.7778.1	17	+	1543	1	intergenic	novelGene_1628	63681044	63682586
PB.7780.5	17	-	2471	1	intergenic	novelGene_1629	64174884	64177354
PB.7781.1	17	+	1374	1	intergenic	novelGene_1630	64335114	64336487
PB.7783.1	17	-	1441	1	intergenic	novelGene_1631	64476555	64477995
PB.7785.1	17	+	1767	1	intergenic	novelGene_1632	65607033	65608799
PB.7789.1	17	-	1907	2	intergenic	novelGene_1633	66168812	66172346
PB.7791.1	17	-	1092	2	antisense	novelGene_ENSCHIG00000016830_AS	66561364	66584117
PB.7793.1	17	-	1306	1	intergenic	novelGene_1634	66676764	66678069
PB.7804.1	18	+	1929	4	antisense	novelGene_ENSCHIG00000020541_AS	86199	98587
PB.7805.1	18	+	1859	1	antisense	novelGene_ENSCHIG00000020541_AS	114452	116310
PB.7817.1	18	+	2399	1	intergenic	novelGene_1635	3001410	3003808
PB.7820.1	18	-	2941	1	intergenic	novelGene_1636	3121796	3124736
PB.7822.11	18	-	2114	1	intergenic	novelGene_1637	3228663	3230776
PB.7822.12	18	-	2467	1	intergenic	novelGene_1638	3266807	3269273
PB.7825.1	18	+	2317	2	antisense	novelGene_ENSC	3386789	3399223

						HIG00000012134_ AS		
PB.7832.1	18	+	1503	2	intergenic	novelGene_1639	3984585	3989992
PB.7833.1	18	-	3509	10	intergenic	novelGene_1640	3990941	4028815
PB.7833.2	18	-	4018	9	intergenic	novelGene_1641	3992481	4028783
PB.7833.3	18	-	2368	10	intergenic	novelGene_1642	3992486	4028797
PB.7833.4	18	-	3164	8	intergenic	novelGene_1643	3997977	4028807
PB.7833.5	18	-	1951	8	intergenic	novelGene_1644	3999216	4028833
PB.7835.1	18	-	1932	1	intergenic	novelGene_1645	4052448	4054379
PB.7845.1	18	-	1579	1	intergenic	novelGene_1646	6827349	6828927
PB.7846.1	18	-	2016	1	intergenic	novelGene_1647	7143054	7145069
PB.7847.1	18	+	1688	1	intergenic	novelGene_1648	7725635	7727322
PB.7858.1	18	-	1810	1	intergenic	novelGene_1649	9331276	9333085
PB.7859.1	18	+	2120	1	intergenic	novelGene_1650	9337459	9339578
PB.7860.1	18	-	1809	1	intergenic	novelGene_1651	9388090	9389898
PB.7865.1	18	+	1914	1	intergenic	novelGene_1652	10505633	10507546
PB.7866.1	18	-	1619	1	intergenic	novelGene_1653	10574155	10575773
PB.7867.1	18	-	2456	1	intergenic	novelGene_1654	10743813	10746268
PB.7869.1	18	-	1389	1	intergenic	novelGene_1655	11178065	11179453
PB.7874.5	18	-	2238	1	intergenic	novelGene_1656	11844475	11846712
PB.7876.3	18	+	3692	1	intergenic	novelGene_1657	11951883	11955574
PB.7876.4	18	+	3250	1	intergenic	novelGene_1658	11952049	11955298
PB.7886.1	18	+	2923	1	intergenic	novelGene_1659	13045756	13048678
PB.7894.1	18	+	1936	1	antisense	novelGene_ENSC HIG00000009862_ AS	14652264	14654199
PB.7894.2	18	+	1861	6	antisense	novelGene_ENSC HIG00000009862_ AS	14652264	14673404
PB.7897.1	18	-	1391	2	antisense	novelGene_ENSC HIG00000004137_ AS	14905938	14909707
PB.7912.5	18	-	2075	1	intergenic	novelGene_1660	15771985	15774059
PB.7912.6	18	-	2444	1	intergenic	novelGene_1661	15841774	15844217
PB.7922.1	18	-	2472	1	intergenic	novelGene_1662	16082330	16084801
PB.7934.1	18	-	1802	1	intergenic	novelGene_1663	18548546	18550347
PB.7935.2	18	-	1338	1	intergenic	novelGene_1664	18597026	18598363
PB.7936.1	18	-	1398	1	intergenic	novelGene_1665	18735033	18736430
PB.7940.1	18	+	3282	1	intergenic	novelGene_1666	20337532	20340813
PB.7946.1	18	-	1730	1	intergenic	novelGene_1667	21666657	21668386
PB.7952.3	18	+	2217	1	intergenic	novelGene_1668	22930356	22932572
PB.7953.1	18	+	1308	1	intergenic	novelGene_1669	23148906	23150213
PB.7957.1	18	-	2607	4	intergenic	novelGene_1670	24599753	24611891
PB.7957.2	18	-	1892	4	intergenic	novelGene_1671	24600292	24611583

PB.7957.3	18	-	1018	4	intergenic	novelGene_1672	24601528	24613282
PB.7961.2	18	+	1012	1	intergenic	novelGene_1673	25207642	25208653
PB.7964.1	18	-	956	1	intergenic	novelGene_1674	25412232	25413187
PB.7964.2	18	-	403	3	intergenic	novelGene_1675	25412233	25413187
PB.7965.1	18	-	407	3	intergenic	novelGene_1676	25450964	25453562
PB.7972.1	18	-	1256	1	antisense	novelGene_ENSCHIG00000021821_AS	26244897	26246152
PB.7975.1	18	+	2576	1	intergenic	novelGene_1677	26375710	26378285
PB.7982.2	18	+	2425	1	intergenic	novelGene_1678	26740265	26742689
PB.7988.4	18	-	1094	1	intergenic	novelGene_1679	27096648	27097741
PB.7996.1	18	+	2175	1	antisense	novelGene_ENSCHIG00000010452_AS	27679289	27681463
PB.7996.2	18	+	1092	1	intergenic	novelGene_1680	27680372	27681463
PB.7997.1	18	+	1839	1	intergenic	novelGene_1681	27705210	27707048
PB.8013.5	18	+	1822	1	intergenic	novelGene_1682	35995103	35996924
PB.8030.1	18	+	2743	1	intergenic	novelGene_1683	36379129	36381871
PB.8031.1	18	-	2111	2	intergenic	novelGene_1684	36383736	36386559
PB.8034.4	18	-	2109	1	intergenic	novelGene_1685	36524613	36526721
PB.8035.1	18	-	2664	1	intergenic	novelGene_1686	36528971	36531634
PB.8039.1	18	-	2322	1	antisense	novelGene_ENSCHIG00000011549_AS	36612960	36615281
PB.8057.1	18	+	1291	2	intergenic	novelGene_1687	37183873	37186359
PB.8059.2	18	+	2295	1	intergenic	novelGene_1688	37346320	37348614
PB.8060.1	18	-	2334	1	intergenic	novelGene_1689	37360563	37362896
PB.8065.1	18	-	1469	1	intergenic	novelGene_1690	37618081	37619549
PB.8070.1	18	+	2685	5	antisense	novelGene_ENSCHIG00000010568_AS	37749869	37753671
PB.8082.1	18	+	1341	1	intergenic	novelGene_1691	40394402	40395742
PB.8104.5	18	-	2011	1	intergenic	novelGene_1692	44898324	44900334
PB.8105.4	18	+	2262	1	intergenic	novelGene_1693	45263572	45265833
PB.8107.1	18	-	1985	1	antisense	novelGene_ENSCHIG00000009458_AS	45685951	45687935
PB.8112.1	18	+	504	3	intergenic	novelGene_1694	46115790	46117972
PB.8114.1	18	+	2223	1	intergenic	novelGene_1696	46287728	46289950
PB.8114.2	18	+	514	3	intergenic	novelGene_1695	46287728	46289947
PB.8127.11	18	-	587	6	intergenic	novelGene_1697	46988815	46993480
PB.8127.12	18	-	1960	4	intergenic	novelGene_1701	46988816	46993480
PB.8127.15	18	-	1852	5	intergenic	novelGene_1702	46988816	46993480
PB.8127.2	18	-	3271	2	intergenic	novelGene_1698	46988815	46993480

PB.8127.3	18	-	2656	3	intergenic	novelGene_1699	46988815	46993480
PB.8127.9	18	-	629	7	intergenic	novelGene_1700	46988815	46993480
PB.8131.1	18	+	3150	19	intergenic	novelGene_1703	47181025	47191150
PB.8142.1	18	+	2847	2	antisense	novelGene_ENSCHIG00000014862_AS	47529363	47534167
PB.8147.1	18	+	3319	3	intergenic	novelGene_1704	47698942	47719796
PB.8148.1	18	-	1439	1	intergenic	novelGene_1705	47700832	47702270
PB.8151.1	18	-	1685	1	intergenic	novelGene_1706	47892373	47894057
PB.8155.1	18	-	2256	2	intergenic	novelGene_1707	48307488	48311173
PB.8169.17	18	+	2401	1	intergenic	novelGene_1709	49311911	49314311
PB.8170.1	18	-	1995	1	intergenic	novelGene_1708	49306163	49308157
PB.8179.1	18	-	2213	1	intergenic	novelGene_1710	49562906	49565118
PB.8182.1	18	-	2188	2	intergenic	novelGene_1711	49789732	49800855
PB.8182.2	18	-	1831	1	intergenic	novelGene_1712	49799031	49800861
PB.8223.2	18	-	1416	1	intergenic	novelGene_1713	51648341	51649756
PB.8224.1	18	+	2254	3	intergenic	novelGene_1714	51679335	51685246
PB.8224.2	18	+	2416	2	intergenic	novelGene_1718	51679365	51685258
PB.8224.3	18	+	2147	3	intergenic	novelGene_1715	51679365	51685194
PB.8224.4	18	+	1997	2	intergenic	novelGene_1716	51679365	51685247
PB.8224.5	18	+	1972	2	intergenic	novelGene_1717	51679365	51685247
PB.8224.6	18	+	2970	1	intergenic	novelGene_1719	51680001	51682970
PB.8224.7	18	+	1600	2	intergenic	novelGene_1720	51680046	51685258
PB.8254.1	18	+	2916	5	intergenic	novelGene_1721	53229487	53241335
PB.8259.4	18	+	1961	1	intergenic	novelGene_1722	53509214	53511174
PB.8260.1	18	-	2195	1	intergenic	novelGene_1723	53551255	53553449
PB.8273.1	18	-	2585	2	antisense	novelGene_ENSCHIG00000026154_AS	53842683	53848256
PB.8296.4	18	-	1305	1	intergenic	novelGene_1724	54440547	54441851
PB.8300.1	18	-	1728	1	intergenic	novelGene_1725	54639880	54641607
PB.8309.1	18	+	3093	1	intergenic	novelGene_1726	55099144	55102236
PB.8309.2	18	+	1806	1	intergenic	novelGene_1727	55100431	55102236
PB.8311.1	18	-	2172	1	intergenic	novelGene_1728	55149662	55151833
PB.8377.5	18	-	1650	1	intergenic	novelGene_1729	57275977	57277626
PB.8378.1	18	+	2955	8	intergenic	novelGene_1730	57290472	57296725
PB.8378.2	18	+	2450	9	intergenic	novelGene_1731	57290488	57296726
PB.8406.1	18	+	2244	2	intergenic	novelGene_1732	58471171	58474030
PB.8409.1	18	-	1913	7	intergenic	novelGene_1733	58516872	58523152
PB.8421.1	18	-	4303	1	antisense	novelGene_ENSCHIG00000023288_AS	59442295	59446597
PB.8422.3	18	+	2834	1	intergenic	novelGene_1734	59635898	59638731
PB.8442.1	18	-	2579	8	intergenic	novelGene_1735	63432317	63454643

PB.8445.1	18	+	1416	1	intergenic	novelGene_1736	63642565	63643980
PB.8460.1	18	+	1258	8	intergenic	novelGene_1737	64114024	64120289
PB.8482.1	18	+	1294	2	intergenic	novelGene_1738	64920777	64924590
PB.8487.1	18	+	2668	5	intergenic	novelGene_1739	65237405	65245075
PB.8489.1	18	-	2448	4	intergenic	novelGene_1740	65284897	65311990
PB.8489.2	18	-	2365	2	intergenic	novelGene_1741	65296611	65311961
PB.8494.1	18	+	4556	5	intergenic	novelGene_1743	65959042	65981133
PB.8494.2	18	+	4360	4	intergenic	novelGene_1742	65959042	65981132
PB.8495.1	18	+	4021	4	intergenic	novelGene_1744	65986151	65992370
PB.8497.1	18	-	3564	4	intergenic	novelGene_1745	66050515	66059049
PB.8502.1	18	-	1689	5	intergenic	novelGene_1746	66242860	66266325
PB.8505.1	18	+	3562	1	antisense	novelGene_ENSCHIG00000019927_AS	66430641	66434202
PB.8510.1	18	-	2257	1	intergenic	novelGene_1747	66802725	66804981
PB.8512.1	18	-	2402	4	antisense	novelGene_ENSCHIG00000016772_AS	66966598	66983236
PB.8513.1	18	+	2679	1	intergenic	novelGene_1748	67045550	67048228
PB.8514.2	18	+	2634	1	intergenic	novelGene_1750	67073473	67076106
PB.8515.1	18	-	1953	2	intergenic	novelGene_1749	67066366	67068937
PB.8517.1	18	+	2669	1	intergenic	novelGene_1751	67094655	67097323
PB.8518.1	18	+	2082	3	intergenic	novelGene_1752	67107160	67114931
PB.8519.3	18	-	2092	1	intergenic	novelGene_1753	67127315	67129406
PB.8524.1	18	+	3899	2	intergenic	novelGene_1754	67222215	67228379
PB.8525.1	18	+	3459	4	intergenic	novelGene_1755	67229039	67235257
PB.8525.2	18	+	3040	4	intergenic	novelGene_1756	67229473	67235257
PB.8525.3	18	+	2505	4	intergenic	novelGene_1757	67229473	67235257
PB.8525.4	18	+	3978	2	intergenic	novelGene_1758	67229486	67235257
PB.8525.5	18	+	2722	4	intergenic	novelGene_1759	67229486	67235257
PB.8525.6	18	+	3590	3	intergenic	novelGene_1760	67229488	67235257
PB.8525.7	18	+	2852	5	intergenic	novelGene_1761	67229491	67235257
PB.8536.1	19	+	1745	1	intergenic	novelGene_1762	8103116	8104860
PB.8542.1	19	+	2980	1	intergenic	novelGene_1763	8497716	8500695
PB.8542.2	19	+	1824	1	intergenic	novelGene_1764	8498873	8500696
PB.8554.1	19	-	1289	1	intergenic	novelGene_1765	10014192	10015480
PB.8557.1	19	-	1173	1	intergenic	novelGene_1766	10282295	10283467
PB.8559.2	19	+	4028	1	intergenic	novelGene_1767	10301727	10305754
PB.8560.1	19	+	2945	1	intergenic	novelGene_1768	10431064	10434008
PB.8564.3	19	+	1206	1	intergenic	novelGene_1769	10697204	10698409
PB.8567.1	19	+	1319	1	intergenic	novelGene_1770	11467176	11468494
PB.8579.1	19	+	1188	1	intergenic	novelGene_1771	13193323	13194510
PB.8580.3	19	-	3929	1	intergenic	novelGene_1772	13299324	13303252
PB.8585.1	19	-	2089	2	intergenic	novelGene_1773	13930795	13933745

PB.8585.2	19	-	1850	2	intergenic	novelGene_1774	13931034	13933745
PB.8586.1	19	+	1934	1	intergenic	novelGene_1775	13949681	13951614
PB.8586.2	19	+	1317	2	intergenic	novelGene_1776	13949681	13951614
PB.8586.3	19	+	493	3	intergenic	novelGene_1777	13949681	13951614
PB.8587.1	19	-	1066	2	intergenic	novelGene_1778	13968903	13970784
PB.8587.2	19	-	1878	1	intergenic	novelGene_1779	13968907	13970784
PB.8587.3	19	-	1312	2	intergenic	novelGene_1780	13968907	13970784
PB.8587.4	19	-	496	3	intergenic	novelGene_1781	13968907	13970784
PB.8591.1	19	+	1231	4	intergenic	novelGene_1782	14090958	14096189
PB.8591.2	19	+	999	4	intergenic	novelGene_1783	14090970	14095966
PB.8591.3	19	+	1579	4	intergenic	novelGene_1784	14090971	14096550
PB.8591.4	19	+	2086	5	intergenic	novelGene_1785	14091141	14105988
PB.8591.6	19	+	550	4	intergenic	novelGene_1786	14091250	14095800
PB.8593.2	19	-	3564	1	intergenic	novelGene_1787	14217372	14220935
PB.8596.1	19	-	893	2	antisense	novelGene_ENSCHIG00000025650_AS	14412596	14415467
PB.8597.1	19	-	1769	1	intergenic	novelGene_1788	14484375	14486143
PB.8602.1	19	+	1908	2	antisense	novelGene_ENSCHIG00000023884_AS	14727373	14731973
PB.8617.1	19	-	1549	1	intergenic	novelGene_1789	17812661	17814209
PB.8618.1	19	+	2967	1	antisense	novelGene_ENSCHIG00000026246_AS	17818412	17821378
PB.8620.1	19	-	1751	1	antisense	novelGene_ENSCHIG00000017954_AS	17887322	17889072
PB.8635.2	19	+	2154	1	intergenic	novelGene_1790	19813283	19815436
PB.8640.2	19	+	2565	1	intergenic	novelGene_1791	19928735	19931299
PB.8653.5	19	-	1248	1	intergenic	novelGene_1792	20262066	20263313
PB.8654.1	19	+	2090	2	antisense	novelGene_ENSCHIG00000011736_AS	20324804	20329779
PB.8655.1	19	-	2531	1	intergenic	novelGene_1793	20422760	20425290
PB.8661.1	19	-	2143	1	intergenic	novelGene_1794	20701459	20703601
PB.8662.1	19	+	2021	1	intergenic	novelGene_1795	20779072	20781092
PB.8663.4	19	-	1941	1	intergenic	novelGene_1796	20803497	20805437
PB.8667.6	19	+	3621	1	intergenic	novelGene_1797	21350112	21353732
PB.8667.7	19	+	1779	1	intergenic	novelGene_1798	21351954	21353732
PB.8671.2	19	+	982	1	intergenic	novelGene_1799	21698068	21699049
PB.8686.5	19	-	2715	2	intergenic	novelGene_1800	22606762	22611504
PB.8686.6	19	-	2896	2	intergenic	novelGene_1801	22607504	22612427
PB.8692.1	19	+	1898	1	intergenic	novelGene_1802	23002605	23004502

PB.8696.1	19	+	2886	1	intergenic	novelGene_1803	23171301	23174186
PB.8697.3	19	-	2297	1	intergenic	novelGene_1804	23235338	23237634
PB.8701.1	19	-	2522	1	intergenic	novelGene_1805	24042974	24045495
PB.8704.1	19	+	2589	1	intergenic	novelGene_1806	24147395	24149983
PB.8736.1	19	+	2814	5	intergenic	novelGene_1807	26087733	26108676
PB.8736.2	19	+	2004	6	intergenic	novelGene_1808	26087733	26108677
PB.8736.3	19	+	2956	5	intergenic	novelGene_1809	26091945	26108677
PB.8747.1	19	+	2295	2	intergenic	novelGene_1810	26360525	26368542
PB.8758.1	19	-	2012	1	antisense	novelGene_ENSCHIG00000019869_AS	26612032	26614043
PB.8791.5	19	-	1783	1	intergenic	novelGene_1811	27125352	27127134
PB.8800.1	19	+	1729	1	intergenic	novelGene_1812	27451058	27452786
PB.8818.1	19	+	2675	1	intergenic	novelGene_1813	28393888	28396562
PB.8818.2	19	+	1507	1	intergenic	novelGene_1814	28395056	28396562
PB.8828.1	19	-	1445	2	intergenic	novelGene_1815	29471299	29477706
PB.8831.1	19	+	3509	1	intergenic	novelGene_1816	30437828	30441336
PB.8831.2	19	+	2427	1	intergenic	novelGene_1817	30438910	30441336
PB.8834.1	19	-	3392	1	antisense	novelGene_ENSCHIG00000010093_AS	31776143	31779534
PB.8836.10	19	-	2147	1	intergenic	novelGene_1818	32451186	32453332
PB.8871.1	19	-	1881	2	intergenic	novelGene_1819	34469596	34475205
PB.8871.2	19	-	1341	2	intergenic	novelGene_1820	34470136	34475205
PB.8875.1	19	-	2340	1	intergenic	novelGene_1821	34594213	34596552
PB.8877.5	19	-	1117	1	intergenic	novelGene_1822	34778453	34779569
PB.8883.1	19	+	1965	1	antisense	novelGene_ENSCHIG00000011901_AS	35149541	35151505
PB.8885.1	19	+	2666	1	intergenic	novelGene_1823	35162042	35164707
PB.8914.1	19	+	1709	1	intergenic	novelGene_1824	36948109	36949817
PB.8920.10	19	-	2606	1	intergenic	novelGene_1825	37337970	37340575
PB.8921.1	19	+	1172	1	intergenic	novelGene_1826	37492350	37493521
PB.8924.1	19	+	1465	1	intergenic	novelGene_1827	37521753	37523217
PB.8926.1	19	-	1290	2	intergenic	novelGene_1828	37576251	37578949
PB.8939.6	19	-	1475	2	intergenic	novelGene_1829	38404010	38427058
PB.8939.7	19	-	1284	2	intergenic	novelGene_1830	38404010	38427146
PB.8940.1	19	+	1444	1	intergenic	novelGene_1831	38553422	38554865
PB.8944.1	19	+	3221	2	intergenic	novelGene_1832	38958530	38981039
PB.8950.1	19	-	503	5	intergenic	novelGene_1833	39092671	39096519
PB.8957.3	19	-	2577	1	intergenic	novelGene_1834	39483483	39486059
PB.8967.1	19	+	1578	3	intergenic	novelGene_1835	39933912	39937083
PB.8976.1	19	-	2337	1	antisense	novelGene_ENSCHIG00000013161_	40077567	40079903

						AS_novelGene_ENSCHIG00000006338_AS		
PB.8991.1	19	+	1139	1	intergenic	novelGene_1836	40650924	40652062
PB.9014.1	19	-	2405	3	intergenic	novelGene_1837	41163190	41165712
PB.9014.2	19	-	2342	2	intergenic	novelGene_1838	41163191	41165712
PB.9037.1	19	+	1667	1	intergenic	novelGene_1839	41790551	41792217
PB.9046.1	19	+	2104	4	antisense	novelGene_ENSCHIG00000020307_AS	42060790	42066561
PB.9049.1	19	+	1501	1	intergenic	novelGene_1840	42196694	42198194
PB.9050.1	19	-	2691	1	intergenic	novelGene_1841	42248795	42251485
PB.9067.1	19	+	1644	2	intergenic	novelGene_1842	42642140	42656742
PB.9073.2	19	+	2449	1	intergenic	novelGene_1843	42810606	42813054
PB.9075.1	19	-	2644	1	intergenic	novelGene_1844	42903456	42906099
PB.9091.1	19	+	1520	1	intergenic	novelGene_1845	43676081	43677600
PB.9097.1	19	-	2637	1	intergenic	novelGene_1846	44158984	44161620
PB.9105.1	19	+	2187	1	intergenic	novelGene_1847	44413958	44416144
PB.9110.1	19	+	2211	1	intergenic	novelGene_1848	44569607	44571817
PB.9111.2	19	-	4050	1	intergenic	novelGene_1849	44580250	44584299
PB.9111.3	19	-	3120	1	intergenic	novelGene_1850	44581422	44584541
PB.9112.1	19	+	1450	1	intergenic	novelGene_1851	44697608	44699057
PB.9118.1	19	+	1788	1	antisense	novelGene_ENSCHIG00000012970_AS	45107605	45109392
PB.9126.1	19	-	1491	1	intergenic	novelGene_1852	46563478	46564968
PB.9128.1	19	+	1313	1	intergenic	novelGene_1853	47003751	47005063
PB.9137.2	19	+	2181	1	intergenic	novelGene_1854	47646771	47648951
PB.9142.6	19	-	1646	1	intergenic	novelGene_1855	47835927	47837572
PB.9143.1	19	-	1380	1	intergenic	novelGene_1856	47881796	47883175
PB.9150.1	19	-	1770	1	intergenic	novelGene_1857	48452102	48453871
PB.9153.2	19	-	3541	1	intergenic	novelGene_1858	49053074	49056614
PB.9156.1	19	-	2382	1	intergenic	novelGene_1859	49203569	49205950
PB.9162.1	19	+	2452	2	intergenic	novelGene_1860	49643821	49646893
PB.9163.2	19	-	2863	11	intergenic	novelGene_1861	49660924	49678722
PB.9175.1	19	+	1594	7	intergenic	novelGene_1862	50235801	50238360
PB.9178.1	19	+	727	5	intergenic	novelGene_1863	50261753	50265355
PB.9188.1	19	-	2990	1	intergenic	novelGene_1864	50392197	50395186
PB.9189.1	19	-	3091	1	intergenic	novelGene_1865	50401476	50404566
PB.9192.1	19	+	1228	5	intergenic	novelGene_1866	50421079	50429100
PB.9192.2	19	+	1522	5	intergenic	novelGene_1867	50421244	50429100
PB.9200.1	19	-	2120	1	antisense	novelGene_ENSCHIG00000008794_AS	50570220	50572339

PB.9202.2	19	+	1413	2	intergenic	novelGene_1868	50622451	50625942
PB.9204.1	19	-	1637	1	intergenic	novelGene_1869	50765545	50767181
PB.9213.1	19	-	1794	1	intergenic	novelGene_1870	51484447	51486240
PB.9231.1	19	-	3216	1	intergenic	novelGene_1871	53049204	53052419
PB.9257.1	19	+	2723	1	intergenic	novelGene_1872	54917248	54919970
PB.9257.2	19	+	1665	1	intergenic	novelGene_1873	54918306	54919970
PB.9309.4	19	+	2425	1	intergenic	novelGene_1874	57724312	57726736
PB.9312.3	19	+	2122	2	intergenic	novelGene_1875	60513567	60515859
PB.9316.1	19	+	1790	1	intergenic	novelGene_1876	60764658	60766447
PB.9317.1	19	+	1713	1	intergenic	novelGene_1877	60766983	60768695
PB.9322.1	19	+	1374	1	intergenic	novelGene_1878	61117148	61118521
PB.9327.1	19	-	3235	2	intergenic	novelGene_1879	62481750	62485794
PB.9327.2	19	-	2364	3	intergenic	novelGene_1880	62481751	62485812
PB.1014.1	2	+	2756	1	intergenic	novelGene_230	113468878	113471633
PB.1020.1	2	-	4017	1	intergenic	novelGene_231	114120134	114124150
PB.1027.19	2	+	2194	1	intergenic	novelGene_232	116651003	116653196
PB.1027.20	2	+	1615	2	intergenic	novelGene_233	116651497	116653194
PB.1034.2	2	+	1764	8	intergenic	novelGene_234	117903307	117927791
PB.1038.2	2	-	3303	11	intergenic	novelGene_235	118035686	118041549
PB.1039.1	2	-	5434	21	antisense	novelGene_ENSCHIG00000000755_AS	118043103	118054802
PB.1040.1	2	-	3950	18	antisense	novelGene_ENSCHIG00000000755_AS	118058571	118065997
PB.1040.10	2	-	3434	17	antisense	novelGene_ENSCHIG00000000755_AS	118065129	118078636
PB.1041.1	2	-	1639	19	intergenic	novelGene_236	118118917	118130121
PB.1041.10	2	-	2840	12	intergenic	novelGene_238	118142567	118151149
PB.1041.2	2	-	4794	30	intergenic	novelGene_237	118126829	118149904
PB.1042.1	2	-	1719	7	intergenic	novelGene_239	118166533	118168945
PB.1042.2	2	-	5336	22	intergenic	novelGene_240	118168227	118206566
PB.1054.1	2	+	2147	1	intergenic	novelGene_241	126603164	126605310
PB.1055.9	2	+	1664	1	intergenic	novelGene_242	126782934	126784597
PB.1059.1	2	+	4992	51	intergenic	novelGene_243	129111131	129150850
PB.1059.10	2	+	4748	51	intergenic	novelGene_264	129111242	129150754
PB.1059.106	2	+	1525	3	intergenic	novelGene_281	129148809	129151435
PB.1059.11	2	+	4714	51	intergenic	novelGene_252	129111242	129150683
PB.1059.12	2	+	4704	51	intergenic	novelGene_253	129111242	129150753
PB.1059.13	2	+	4559	48	intergenic	novelGene_254	129111242	129150753
PB.1059.14	2	+	4471	50	intergenic	novelGene_251	129111242	129149707
PB.1059.15	2	+	4464	44	intergenic	novelGene_266	129111242	129151018
PB.1059.16	2	+	4443	46	intergenic	novelGene_255	129111242	129150753

PB.1059.17	2	+	4321	1	intergenic	novelGene_246	129111242	129115562
PB.1059.18	2	+	4316	44	intergenic	novelGene_256	129111242	129150753
PB.1059.19	2	+	4199	44	intergenic	novelGene_257	129111242	129150753
PB.1059.20	2	+	4002	41	intergenic	novelGene_258	129111242	129150753
PB.1059.21	2	+	3891	28	intergenic	novelGene_268	129111242	129151435
PB.1059.22	2	+	3750	36	intergenic	novelGene_265	129111242	129150757
PB.1059.23	2	+	3209	28	intergenic	novelGene_259	129111242	129150753
PB.1059.24	2	+	2793	22	intergenic	novelGene_260	129111242	129150753
PB.1059.25	2	+	2718	21	intergenic	novelGene_261	129111242	129150753
PB.1059.26	2	+	2112	1	intergenic	novelGene_245	129111242	129113353
PB.1059.27	2	+	2107	4	intergenic	novelGene_247	129111242	129125331
PB.1059.28	2	+	1931	4	intergenic	novelGene_248	129111242	129125331
PB.1059.29	2	+	1881	24	intergenic	novelGene_250	129111242	129134637
PB.1059.3	2	+	5491	51	intergenic	novelGene_271	129111242	129151460
PB.1059.30	2	+	1775	1	intergenic	novelGene_244	129111242	129113016
PB.1059.31	2	+	1686	5	intergenic	novelGene_249	129111242	129125331
PB.1059.42	2	+	3872	41	intergenic	novelGene_272	129129095	129150753
PB.1059.49	2	+	3472	34	intergenic	novelGene_273	129132438	129150753
PB.1059.5	2	+	5125	46	intergenic	novelGene_269	129111242	129151435
PB.1059.59	2	+	3690	29	intergenic	novelGene_275	129134327	129151385
PB.1059.6	2	+	5049	51	intergenic	novelGene_267	129111242	129151018
PB.1059.60	2	+	3058	29	intergenic	novelGene_274	129134327	129150753
PB.1059.7	2	+	4899	50	intergenic	novelGene_262	129111242	129150753
PB.1059.73	2	+	3228	21	intergenic	novelGene_276	129137380	129151435
PB.1059.77	2	+	1828	1	intergenic	novelGene_277	129139468	129141295
PB.1059.78	2	+	2448	19	intergenic	novelGene_278	129139521	129150818
PB.1059.79	2	+	3014	18	intergenic	novelGene_279	129139656	129151435
PB.1059.8	2	+	4881	44	intergenic	novelGene_270	129111242	129151435
PB.1059.9	2	+	4769	51	intergenic	novelGene_263	129111242	129150753
PB.1059.91	2	+	1736	11	intergenic	novelGene_280	129143917	129150753
PB.1060.1	2	+	1304	1	intergenic	novelGene_282	129308853	129310156
PB.1073.1	2	-	2498	1	intergenic	novelGene_283	131293900	131296397
PB.1074.1	2	-	2188	1	intergenic	novelGene_284	131299853	131302040
PB.1076.1	2	-	1737	1	intergenic	novelGene_285	131624269	131626005
PB.1086.1	2	+	1987	1	intergenic	novelGene_286	134566249	134568235
PB.1092.1	2	-	1976	1	intergenic	novelGene_287	135390647	135392622
PB.1093.12	2	-	3214	1	intergenic	novelGene_288	135505181	135508394
PB.544.1	2	+	1406	3	antisense	novelGene_ENSCHIG00000025682_AS	1119978	1122286
PB.544.2	2	+	1506	2	antisense	novelGene_ENSCHIG00000025682_AS	1119979	1122287
PB.545.1	2	+	1468	3	intergenic	novelGene_144	1150072	1161158

PB.547.1	2	-	2250	1	intergenic	novelGene_145	2296750	2298999
PB.549.1	2	+	1363	1	intergenic	novelGene_146	2348008	2349370
PB.550.1	2	+	3142	2	intergenic	novelGene_147	2447629	2450965
PB.556.6	2	-	2637	2	intergenic	novelGene_148	3078660	3083054
PB.563.6	2	+	2244	1	intergenic	novelGene_149	3940729	3942972
PB.571.1	2	+	1988	1	antisense	novelGene_ENSCHIG00000023316_AS	4871418	4873405
PB.575.9	2	+	1756	1	intergenic	novelGene_150	5180811	5182566
PB.576.1	2	-	1661	1	intergenic	novelGene_151	5210370	5212030
PB.583.1	2	-	2069	1	intergenic	novelGene_152	6143679	6145747
PB.585.1	2	+	1202	1	intergenic	novelGene_153	6415371	6416572
PB.600.1	2	+	2460	3	intergenic	novelGene_154	6988062	6992531
PB.600.2	2	+	3156	2	intergenic	novelGene_156	6988621	6992535
PB.600.3	2	+	2436	3	intergenic	novelGene_155	6988621	6992531
PB.601.1	2	+	1467	1	intergenic	novelGene_157	7003832	7005298
PB.606.1	2	+	2380	1	intergenic	novelGene_158	7548320	7550699
PB.609.1	2	-	2674	1	intergenic	novelGene_159	7752714	7755387
PB.618.1	2	-	2179	2	intergenic	novelGene_160	8725905	8736944
PB.631.4	2	+	966	2	intergenic	novelGene_161	9310725	9312117
PB.633.1	2	+	1843	1	intergenic	novelGene_162	9546461	9548303
PB.643.1	2	+	1951	2	intergenic	novelGene_164	9771230	9775730
PB.643.2	2	+	1668	2	intergenic	novelGene_163	9771230	9775447
PB.648.1	2	+	2896	1	antisense	novelGene_ENSCHIG00000023030_AS_novelGene_ENSCHIG00000004209_AS	10049463	10052358
PB.662.2	2	-	2174	1	intergenic	novelGene_165	10579423	10581596
PB.667.1	2	-	2643	1	intergenic	novelGene_166	10892707	10895349
PB.669.4	2	+	694	3	intergenic	novelGene_167	11048993	11051261
PB.675.4	2	+	1685	1	intergenic	novelGene_168	11240022	11241706
PB.676.5	2	+	1746	1	intergenic	novelGene_169	11399351	11401096
PB.682.7	2	-	1741	1	intergenic	novelGene_170	13327205	13328945
PB.682.8	2	-	1596	1	intergenic	novelGene_171	13367600	13369195
PB.689.1	2	+	2042	1	intergenic	novelGene_173	14158126	14160167
PB.690.3	2	-	1316	1	intergenic	novelGene_172	14138333	14139648
PB.694.1	2	+	1544	1	intergenic	novelGene_174	14354377	14355920
PB.700.1	2	+	1553	1	antisense	novelGene_ENSCHIG00000013777_AS	14407966	14409518
PB.700.2	2	+	1937	1	antisense	novelGene_ENSCHIG00000013777_AS	14408084	14410020

PB.709.1	2	+	1234	1	intergenic	novelGene_175	14837929	14839162
PB.713.8	2	-	2974	1	intergenic	novelGene_176	14999222	15002195
PB.718.3	2	-	1347	1	intergenic	novelGene_177	15520831	15522177
PB.724.1	2	-	1218	5	intergenic	novelGene_178	16306362	16311427
PB.724.4	2	-	941	5	intergenic	novelGene_179	16306637	16311425
PB.724.5	2	-	538	5	intergenic	novelGene_180	16307040	16311425
PB.725.1	2	-	2443	2	intergenic	novelGene_181	16324324	16326841
PB.727.2	2	-	1539	1	intergenic	novelGene_182	16752809	16754347
PB.728.1	2	+	1923	1	intergenic	novelGene_183	16862930	16864852
PB.737.1	2	-	1671	1	antisense	novelGene_ENSCHIG00000013398_AS	17826319	17827989
PB.740.1	2	+	1240	1	intergenic	novelGene_185	19824781	19826020
PB.740.2	2	+	1078	1	intergenic	novelGene_184	19824781	19825858
PB.742.1	2	+	1813	1	intergenic	novelGene_186	20071700	20073512
PB.758.5	2	-	1430	1	intergenic	novelGene_187	25094186	25095615
PB.759.6	2	+	2107	1	intergenic	novelGene_188	26231145	26233251
PB.779.1	2	+	2471	3	antisense	novelGene_ENSCHIG00000022853_AS	28957059	28964377
PB.800.1	2	+	2664	1	intergenic	novelGene_189	29894113	29896776
PB.803.2	2	-	362	4	intergenic	novelGene_190	31306017	31308480
PB.810.1	2	+	1864	1	intergenic	novelGene_191	32662962	32664825
PB.812.2	2	+	2409	1	intergenic	novelGene_192	37734804	37737212
PB.819.1	2	+	2236	1	intergenic	novelGene_193	40070795	40073030
PB.829.3	2	-	2211	1	intergenic	novelGene_194	44004922	44007132
PB.834.1	2	-	2660	1	intergenic	novelGene_195	44895155	44897814
PB.835.1	2	+	1873	1	intergenic	novelGene_196	44989500	44991372
PB.840.1	2	-	3140	1	intergenic	novelGene_197	45627497	45630636
PB.846.1	2	+	2081	1	intergenic	novelGene_198	46252742	46254822
PB.847.9	2	-	1722	1	intergenic	novelGene_199	46261719	46263440
PB.874.2	2	-	2015	1	intergenic	novelGene_200	51612024	51614038
PB.878.1	2	+	1385	1	intergenic	novelGene_201	56186808	56188192
PB.882.1	2	+	1301	1	antisense	novelGene_ENSCHIG00000008500_AS	56509934	56511234
PB.896.1	2	-	1921	1	intergenic	novelGene_202	65085754	65087674
PB.900.1	2	+	1454	1	intergenic	novelGene_203	70673589	70675042
PB.901.1	2	+	1907	4	intergenic	novelGene_204	70722945	70841147
PB.904.1	2	-	1336	1	intergenic	novelGene_205	71430994	71432329
PB.907.1	2	-	1359	1	intergenic	novelGene_206	73895625	73896983
PB.912.1	2	+	1675	1	intergenic	novelGene_207	74797679	74799353
PB.917.1	2	-	848	1	intergenic	novelGene_208	77622385	77623232
PB.918.1	2	-	1354	1	intergenic	novelGene_209	77632120	77633473

PB.921.1	2	+	2041	1	intergenic	novelGene_211	84115676	84117716
PB.922.8	2	-	1651	1	intergenic	novelGene_210	84107134	84108784
PB.922.9	2	-	1680	1	intergenic	novelGene_212	84126234	84127913
PB.927.1	2	-	1716	1	antisense	novelGene_ENSCHIG00000018947_AS	89541348	89543063
PB.934.1	2	-	3268	1	antisense	novelGene_ENSCHIG00000020327_AS	91543691	91546958
PB.935.1	2	+	1974	1	intergenic	novelGene_213	91549303	91551276
PB.943.1	2	+	1172	1	intergenic	novelGene_214	92186404	92187575
PB.945.1	2	+	1984	1	intergenic	novelGene_215	92377117	92379100
PB.946.1	2	+	1793	1	intergenic	novelGene_216	92390780	92392572
PB.947.1	2	+	1501	1	intergenic	novelGene_217	92405577	92407077
PB.956.1	2	-	2281	1	intergenic	novelGene_218	98462431	98464711
PB.957.1	2	+	3740	1	intergenic	novelGene_219	98800231	98803970
PB.958.1	2	+	2666	1	intergenic	novelGene_220	98863709	98866374
PB.962.1	2	+	2018	1	intergenic	novelGene_221	99195796	99197813
PB.964.1	2	+	2282	1	antisense	novelGene_ENSCHIG00000022217_AS	99460856	99463137
PB.964.2	2	+	1748	1	antisense	novelGene_ENSCHIG00000022217_AS	99461030	99462777
PB.965.9	2	+	3796	1	intergenic	novelGene_222	99553748	99557543
PB.970.1	2	-	1569	1	intergenic	novelGene_223	100074883	100076451
PB.970.2	2	-	1226	1	intergenic	novelGene_224	100075230	100076455
PB.971.1	2	+	1344	2	antisense	novelGene_ENSCHIG00000023380_AS	100087702	100090094
PB.972.1	2	+	2949	1	intergenic	novelGene_225	100143314	100146262
PB.972.2	2	+	1052	1	intergenic	novelGene_226	100145211	100146262
PB.973.1	2	+	1439	1	intergenic	novelGene_227	100169583	100171021
PB.974.9	2	-	1686	1	intergenic	novelGene_228	100229027	100230712
PB.988.1	2	-	1555	1	intergenic	novelGene_229	108579715	108581269
PB.9328.1	20	-	1900	1	intergenic	novelGene_1881	354171	356070
PB.9330.1	20	-	2476	1	intergenic	novelGene_1882	502193	504668
PB.9331.1	20	+	1611	1	intergenic	novelGene_1883	824609	826219
PB.9332.1	20	-	2004	1	intergenic	novelGene_1884	1020358	1022361
PB.9333.1	20	+	1587	1	intergenic	novelGene_1885	1039161	1040747
PB.9341.1	20	+	1651	1	intergenic	novelGene_1886	3843992	3845642
PB.9343.2	20	-	1876	2	intergenic	novelGene_1887	3901285	4031582
PB.9344.1	20	-	1780	1	intergenic	novelGene_1888	4051785	4053564
PB.9345.1	20	-	3088	1	intergenic	novelGene_1889	4151428	4154515

PB.9345.2	20	-	1955	1	intergenic	novelGene_1890	4151429	4153383
PB.9348.6	20	+	2759	1	intergenic	novelGene_1891	4656524	4659282
PB.9361.4	20	+	1696	1	intergenic	novelGene_1892	8096009	8097704
PB.9383.1	20	+	2035	1	intergenic	novelGene_1895	11399995	11402029
PB.9384.1	20	+	1356	1	intergenic	novelGene_1896	11423176	11424531
PB.9385.1	20	+	1343	1	intergenic	novelGene_1897	11481202	11482544
PB.9386.1	20	+	2298	1	intergenic	novelGene_1899	11494759	11497056
PB.9387.1	20	-	2230	1	intergenic	novelGene_1893	11398328	11400557
PB.9387.2	20	-	2258	1	intergenic	novelGene_1894	11398643	11400900
PB.9388.8	20	-	3416	1	intergenic	novelGene_1898	11492060	11495475
PB.9389.1	20	-	2126	1	intergenic	novelGene_1900	12426736	12428861
PB.9390.1	20	-	2054	1	intergenic	novelGene_1901	12473907	12475960
PB.9392.3	20	-	1662	1	intergenic	novelGene_1902	12583825	12585486
PB.9393.1	20	-	1625	1	intergenic	novelGene_1903	13441514	13443138
PB.9397.1	20	+	1778	1	intergenic	novelGene_1904	13632324	13634101
PB.9411.1	20	+	2482	1	intergenic	novelGene_1905	18017036	18019517
PB.9412.1	20	-	2559	1	intergenic	novelGene_1906	18028348	18030906
PB.9418.1	20	+	2944	2	antisense	novelGene_ENSCHIG00000006526_AS	18787456	18914501
PB.9421.2	20	-	1892	1	intergenic	novelGene_1907	20863951	20865842
PB.9424.6	20	-	1682	2	intergenic	novelGene_1908	22143515	22146385
PB.9424.7	20	-	1016	2	intergenic	novelGene_1909	22144183	22146387
PB.9429.1	20	+	2147	1	antisense	novelGene_ENSCHIG00000024413_AS	23826731	23828877
PB.9433.2	20	-	1479	1	intergenic	novelGene_1910	24635528	24637006
PB.9436.1	20	-	1274	1	intergenic	novelGene_1911	25065421	25066694
PB.9440.1	20	-	1998	1	antisense	novelGene_ENSCHIG00000014594_AS	26010430	26012427
PB.9442.1	20	-	2369	1	intergenic	novelGene_1912	26151534	26153902
PB.9448.1	20	-	2983	1	antisense	novelGene_ENSCHIG00000012683_AS	30469383	30472365
PB.9451.1	20	+	2020	1	intergenic	novelGene_1913	31271756	31273775
PB.9456.4	20	-	1832	1	intergenic	novelGene_1914	32606470	32608301
PB.9465.1	20	-	2101	1	antisense	novelGene_ENSCHIG00000009529_AS	35037221	35039321
PB.9466.1	20	-	1861	1	intergenic	novelGene_1915	35041418	35043278
PB.9468.1	20	+	3146	2	intergenic	novelGene_1916	35344616	35347932
PB.9468.3	20	+	1973	1	intergenic	novelGene_1917	35397555	35399527
PB.9469.1	20	-	1871	1	intergenic	novelGene_1918	35448758	35450628

PB.9470.1	20	+	1572	1	intergenic	novelGene_1919	35514077	35515648
PB.9472.1	20	-	3358	1	intergenic	novelGene_1920	35846220	35849577
PB.9476.1	20	-	1459	1	intergenic	novelGene_1921	37110857	37112315
PB.9477.8	20	-	1653	1	intergenic	novelGene_1922	37249672	37251324
PB.9477.9	20	-	2005	2	intergenic	novelGene_1923	37304816	37341361
PB.9478.1	20	+	3362	1	intergenic	novelGene_1925	37341475	37344836
PB.9478.2	20	+	3257	1	intergenic	novelGene_1924	37341475	37344731
PB.9493.1	20	-	2187	1	intergenic	novelGene_1926	40864390	40866576
PB.9497.1	20	-	2514	1	intergenic	novelGene_1927	41271362	41273875
PB.9508.1	20	-	2433	1	intergenic	novelGene_1928	57170617	57173049
PB.9512.1	20	-	1359	1	intergenic	novelGene_1929	58572118	58573476
PB.9521.1	20	-	1648	2	intergenic	novelGene_1930	63658021	63662825
PB.9525.1	20	-	2045	1	intergenic	novelGene_1931	66477095	66479139
PB.9531.1	20	+	826	2	intergenic	novelGene_1932	70832504	70833716
PB.9543.4	21	+	1474	2	intergenic	novelGene_1933	1092012	1099267
PB.9544.1	21	+	3621	3	intergenic	novelGene_1934	1128609	1135474
PB.9544.2	21	+	1435	3	intergenic	novelGene_1935	1130795	1135474
PB.9546.1	21	+	3003	1	intergenic	novelGene_1936	1497991	1500993
PB.9551.5	21	+	1372	1	intergenic	novelGene_1937	5444696	5446067
PB.9554.3	21	+	1273	1	intergenic	novelGene_1938	6674607	6675879
PB.9555.1	21	-	1673	1	intergenic	novelGene_1939	6754912	6756584
PB.9558.1	21	+	2630	1	intergenic	novelGene_1940	7116900	7119529
PB.9559.1	21	+	2308	1	intergenic	novelGene_1941	7159035	7161342
PB.9560.1	21	-	2038	1	intergenic	novelGene_1942	7171064	7173101
PB.9561.1	21	-	2147	1	intergenic	novelGene_1943	7216825	7218971
PB.9562.1	21	+	2288	1	intergenic	novelGene_1944	7339856	7342143
PB.9564.1	21	-	2042	1	intergenic	novelGene_1945	9816646	9818687
PB.9567.1	21	-	2338	1	intergenic	novelGene_1946	13306745	13309082
PB.9568.1	21	-	1673	1	intergenic	novelGene_1947	13317561	13319233
PB.9569.1	21	+	2211	1	intergenic	novelGene_1948	13408106	13410316
PB.9571.1	21	+	2131	1	intergenic	novelGene_1949	14143108	14145238
PB.9573.4	21	+	2727	1	intergenic	novelGene_1950	15828336	15831062
PB.9573.5	21	+	1456	1	intergenic	novelGene_1951	15829607	15831062
PB.9581.1	21	+	2748	1	intergenic	novelGene_1952	18965750	18968497
PB.9584.1	21	-	1458	1	intergenic	novelGene_1953	19217688	19219145
PB.9588.1	21	+	3016	1	intergenic	novelGene_1954	19989922	19992937
PB.9588.2	21	+	1966	1	intergenic	novelGene_1955	19990972	19992937
PB.9592.1	21	-	2022	1	intergenic	novelGene_1956	20351447	20353468
PB.9598.1	21	-	2814	6	intergenic	novelGene_1957	20541686	20584502
PB.9601.4	21	-	2406	1	intergenic	novelGene_1958	20784662	20787067
PB.9615.1	21	+	2046	1	antisense	novelGene_ENSCHIG00000010086_AS	21434002	21436047
PB.9616.11	21	-	1381	1	intergenic	novelGene_1959	21391151	21392531

PB.9616.24	21	-	1533	1	intergenic	novelGene_1960	21486746	21488278
PB.9616.26	21	-	1993	1	intergenic	novelGene_1961	21491110	21493102
PB.9619.5	21	-	2257	1	intergenic	novelGene_1962	21644267	21646523
PB.9625.1	21	+	2122	1	intergenic	novelGene_1963	22405020	22407141
PB.9628.1	21	+	1971	1	antisense	novelGene_ENSCHIG00000015479_AS	22555068	22557038
PB.9631.2	21	-	1756	1	intergenic	novelGene_1964	23484208	23485963
PB.9638.1	21	-	4024	2	antisense	novelGene_ENSCHIG00000005376_AS	24686750	24691044
PB.9638.2	21	-	2274	2	antisense	novelGene_ENSCHIG00000005376_AS	24686750	24691023
PB.9638.3	21	-	1529	2	antisense	novelGene_ENSCHIG00000005376_AS	24686750	24691052
PB.9643.8	21	+	2136	1	intergenic	novelGene_1965	25701851	25703986
PB.9645.1	21	-	2817	2	antisense	novelGene_ENSCHIG00000022641_AS	25964360	25972213
PB.9647.1	21	+	2625	5	intergenic	novelGene_1966	26265448	26356007
PB.9648.1	21	+	1302	1	intergenic	novelGene_1967	26424373	26425674
PB.9653.1	21	+	2651	2	intergenic	novelGene_1968	26937539	26945185
PB.9658.1	21	+	2023	1	intergenic	novelGene_1969	27045521	27047543
PB.9660.1	21	-	1935	1	antisense	novelGene_ENSCHIG00000015732_AS	27286416	27288350
PB.9661.1	21	+	3072	1	intergenic	novelGene_1970	27296388	27299459
PB.9661.2	21	+	2009	1	intergenic	novelGene_1971	27297345	27299353
PB.9663.1	21	-	1898	1	intergenic	novelGene_1972	28078978	28080875
PB.9664.1	21	-	1937	1	intergenic	novelGene_1973	28111014	28112950
PB.9668.1	21	+	2093	3	intergenic	novelGene_1974	28860510	28931289
PB.9670.1	21	-	2564	1	intergenic	novelGene_1975	29973474	29976037
PB.9689.1	21	-	970	1	intergenic	novelGene_1976	32016288	32017257
PB.9693.1	21	+	1983	1	intergenic	novelGene_1977	32686976	32688958
PB.9723.1	21	-	1670	1	antisense	novelGene_ENSCHIG00000007305_AS	40978961	40980630
PB.9726.6	21	-	1708	1	intergenic	novelGene_1978	41208031	41209738
PB.9731.1	21	+	1695	1	intergenic	novelGene_1979	42872019	42873713
PB.9733.1	21	+	1982	1	intergenic	novelGene_1980	44306887	44308868
PB.9744.1	21	-	2505	2	intergenic	novelGene_1981	44955306	44964146
PB.9776.1	21	+	1141	2	intergenic	novelGene_1982	54921100	54922858

PB.9776.2	21	+	2182	4	antisense	novelGene_ENSCHIG00000020878_AS	54921109	54924876
PB.9776.3	21	+	3089	2	antisense	novelGene_ENSCHIG00000020878_AS	54921115	54924821
PB.9776.4	21	+	2525	3	antisense	novelGene_ENSCHIG00000020878_AS	54921115	54924878
PB.9776.5	21	+	2139	4	antisense	novelGene_ENSCHIG00000020878_AS	54921115	54924816
PB.9776.6	21	+	1951	3	antisense	novelGene_ENSCHIG00000020878_AS	54921115	54924878
PB.9776.7	21	+	504	3	intergenic	novelGene_1983	54921115	54922857
PB.9781.7	21	-	2178	1	intergenic	novelGene_1984	55728456	55730633
PB.9782.1	21	-	1895	1	intergenic	novelGene_1985	56042304	56044198
PB.9783.1	21	+	1680	11	antisense	novelGene_ENSCHIG00000017489_AS	56047414	56134060
PB.9788.1	21	-	346	3	intergenic	novelGene_1986	56276947	56287884
PB.9791.5	21	-	2129	1	intergenic	novelGene_1987	56880432	56882560
PB.9796.1	21	-	1503	3	intergenic	novelGene_1988	57289459	57297216
PB.9796.2	21	-	1355	2	intergenic	novelGene_1989	57289459	57297228
PB.9798.1	21	-	2178	1	intergenic	novelGene_1990	57324338	57326515
PB.9806.1	21	-	1640	1	intergenic	novelGene_1991	59295276	59296915
PB.9808.1	21	+	1901	2	intergenic	novelGene_1992	60228330	60275116
PB.9813.1	21	-	1363	1	intergenic	novelGene_1993	60682694	60684056
PB.9815.1	21	-	2832	3	intergenic	novelGene_1994	63231996	63253923
PB.9815.2	21	-	2862	1	intergenic	novelGene_1995	63251094	63253955
PB.9827.1	21	+	3305	3	intergenic	novelGene_1997	64994986	65001178
PB.9827.2	21	+	3291	3	intergenic	novelGene_1998	64994986	65001204
PB.9827.3	21	+	1792	3	intergenic	novelGene_1996	64994986	64999665
PB.9828.1	21	-	1665	1	intergenic	novelGene_1999	65104971	65106635
PB.9830.1	21	+	1971	1	intergenic	novelGene_2000	65752260	65754230
PB.9832.1	21	-	2078	1	intergenic	novelGene_2001	66025136	66027213
PB.9833.1	21	+	1886	1	intergenic	novelGene_2002	66093635	66095520
PB.9841.2	21	+	2653	2	intergenic	novelGene_2003	67057128	67066039
PB.9850.1	21	-	369	4	intergenic	novelGene_2004	67734995	67740250
PB.9851.1	21	-	3027	1	intergenic	novelGene_2005	68367589	68370615
PB.9852.4	21	+	3186	1	intergenic	novelGene_2006	68594252	68597437
PB.9858.1	21	-	2683	2	intergenic	novelGene_2007	68807468	68810627
PB.9858.2	21	-	3628	2	intergenic	novelGene_2008	68809647	68826836

PB.9858.3	21	-	1978	2	intergenic	novelGene_2010	68811308	68826847
PB.9858.4	21	-	320	1	intergenic	novelGene_2009	68811308	68811627
PB.9860.1	21	+	1784	2	intergenic	novelGene_2011	68879688	68882006
PB.9861.19	21	+	3207	1	intergenic	novelGene_2012	68998281	69001487
PB.10007.1	22	-	1990	1	intergenic	novelGene_2051	35009778	35011767
PB.10010.1	22	-	1140	1	intergenic	novelGene_2052	36843348	36844487
PB.10011.1	22	+	1879	1	intergenic	novelGene_2053	36845190	36847068
PB.10012.1	22	+	1740	1	intergenic	novelGene_2054	36847661	36849400
PB.10013.1	22	+	2303	1	intergenic	novelGene_2055	37201754	37204056
PB.10014.1	22	+	2352	1	intergenic	novelGene_2056	37230841	37233192
PB.10023.1	22	+	2087	1	intergenic	novelGene_2057	42807708	42809794
PB.10027.8	22	-	2691	1	intergenic	novelGene_2058	42956167	42958857
PB.10030.11	22	-	1706	1	intergenic	novelGene_2059	43207031	43208736
PB.10031.1	22	-	1909	4	intergenic	novelGene_2060	43216342	43223803
PB.10036.1	22	-	2486	1	intergenic	novelGene_2061	43771694	43774179
PB.10039.3	22	+	2082	1	intergenic	novelGene_2062	44130211	44132292
PB.10043.3	22	+	2890	1	intergenic	novelGene_2063	46931786	46934675
PB.10062.1	22	-	2826	22	antisense	novelGene_ENSCHIG00000023928_AS	48247876	48253144
PB.10093.2	22	+	1536	1	intergenic	novelGene_2064	50090541	50092076
PB.10117.1	22	-	2185	1	intergenic	novelGene_2065	50836187	50838371
PB.10128.6	22	-	2531	1	intergenic	novelGene_2066	51107468	51109998
PB.10129.1	22	+	2230	1	intergenic	novelGene_2067	51110018	51112247
PB.10131.1	22	-	1411	1	intergenic	novelGene_2068	51169276	51170686
PB.10132.1	22	+	2723	1	intergenic	novelGene_2069	51225733	51228455
PB.10132.2	22	+	2563	1	intergenic	novelGene_2070	51226339	51228901
PB.10132.3	22	+	1436	1	intergenic	novelGene_2071	51227020	51228455
PB.10137.1	22	+	2550	37	intergenic	novelGene_2072	51373209	51382793
PB.10142.1	22	-	382	4	intergenic	novelGene_2073	51478212	51480177
PB.10142.2	22	-	1970	1	intergenic	novelGene_2074	51478213	51480182
PB.10163.1	22	-	1575	2	intergenic	novelGene_2075	53155185	53167937
PB.10165.1	22	+	1921	2	intergenic	novelGene_2076	53274965	53282503
PB.10171.1	22	+	3141	1	intergenic	novelGene_2077	53603018	53606158
PB.10179.6	22	+	2768	2	intergenic	novelGene_2078	54264807	54268647
PB.10183.8	22	-	2892	1	intergenic	novelGene_2079	55579556	55582447
PB.10185.3	22	+	1232	1	intergenic	novelGene_2082	56004475	56005706
PB.10186.1	22	-	1865	1	intergenic	novelGene_2080	55990720	55992584
PB.10187.1	22	-	2009	1	intergenic	novelGene_2081	55995295	55997303
PB.10196.3	22	-	1548	2	intergenic	novelGene_2083	56780274	56804103
PB.10200.1	22	-	2440	1	intergenic	novelGene_2084	57227477	57229916
PB.10200.2	22	-	1833	1	intergenic	novelGene_2085	57228529	57230361
PB.10201.1	22	+	3286	1	intergenic	novelGene_2086	57341353	57344638
PB.10210.3	22	-	1871	1	intergenic	novelGene_2087	58965041	58966911

PB.10213.1	22	+	2345	1	intergenic	novelGene_2088	59036330	59038674
PB.10216.1	22	+	1371	2	intergenic	novelGene_2089	59151675	59153706
PB.10216.2	22	+	2016	1	intergenic	novelGene_2090	59151690	59153705
PB.10219.1	22	-	3688	2	intergenic	novelGene_2091	59400611	59404798
PB.10223.1	22	-	1370	1	intergenic	novelGene_2092	59590327	59591696
PB.10224.3	22	+	1851	1	intergenic	novelGene_2093	59657373	59659223
PB.9869.1	22	-	1844	1	intergenic	novelGene_2013	769391	771234
PB.9870.6	22	-	1904	1	intergenic	novelGene_2014	937301	939204
PB.9870.7	22	-	2181	1	intergenic	novelGene_2015	960644	962824
PB.9870.8	22	-	2076	1	intergenic	novelGene_2016	971517	973592
PB.9871.2	22	+	1374	1	intergenic	novelGene_2017	1304331	1305704
PB.9878.1	22	+	2237	1	intergenic	novelGene_2018	6182381	6184617
PB.9881.6	22	-	2216	1	intergenic	novelGene_2019	6822780	6824995
PB.9887.1	22	+	1952	1	intergenic	novelGene_2020	7387261	7389212
PB.9891.1	22	+	3589	1	intergenic	novelGene_2021	9472756	9476344
PB.9896.1	22	+	1851	1	intergenic	novelGene_2022	10434153	10436003
PB.9899.1	22	+	4136	1	intergenic	novelGene_2023	11205662	11209797
PB.9900.1	22	-	2157	1	intergenic	novelGene_2024	11224010	11226166
PB.9901.1	22	+	1407	1	intergenic	novelGene_2025	11310047	11311453
PB.9920.1	22	-	2206	4	antisense	novelGene_ENSCHIG00000025599_AS	13196911	13223499
PB.9922.1	22	+	3458	2	intergenic	novelGene_2026	14212510	14220876
PB.9922.2	22	+	2815	3	intergenic	novelGene_2027	14212510	14220876
PB.9924.1	22	-	2972	1	intergenic	novelGene_2028	14341125	14344096
PB.9937.1	22	+	1990	1	intergenic	novelGene_2029	15141025	15143014
PB.9938.1	22	+	1657	1	antisense	novelGene_ENSCHIG00000012109_AS	15308144	15309800
PB.9941.1	22	+	3541	1	intergenic	novelGene_2030	15454586	15458126
PB.9942.1	22	-	2108	4	intergenic	novelGene_2031	16189768	16202016
PB.9942.2	22	-	1898	4	intergenic	novelGene_2032	16191370	16201992
PB.9944.1	22	+	1728	4	intergenic	novelGene_2033	16224014	16235195
PB.9945.1	22	+	1390	1	intergenic	novelGene_2034	16237238	16238627
PB.9948.2	22	+	1410	1	intergenic	novelGene_2035	16287461	16288870
PB.9950.4	22	-	2210	1	intergenic	novelGene_2036	16512506	16514715
PB.9969.1	22	-	2545	1	intergenic	novelGene_2037	16966847	16969391
PB.9970.3	22	-	2136	1	intergenic	novelGene_2038	17028565	17030700
PB.9971.1	22	+	3489	4	intergenic	novelGene_2039	17030846	17046049
PB.9971.2	22	+	4316	4	intergenic	novelGene_2043	17030904	17046504
PB.9971.3	22	+	4010	4	intergenic	novelGene_2040	17030904	17046119
PB.9971.4	22	+	3936	4	intergenic	novelGene_2042	17030904	17046124
PB.9971.5	22	+	3780	3	intergenic	novelGene_2041	17030904	17046120
PB.9976.1	22	-	2801	1	intergenic	novelGene_2044	21029731	21032531

PB.9985.1	22	+	2808	1	intergenic	novelGene_2045	28112851	28115658
PB.9986.1	22	+	2267	1	intergenic	novelGene_2046	28216761	28219027
PB.9988.1	22	-	1294	1	intergenic	novelGene_2047	28293218	28294511
PB.9991.1	22	-	1894	1	intergenic	novelGene_2048	29151487	29153380
PB.9995.1	22	-	2254	1	intergenic	novelGene_2049	30159928	30162181
PB.9997.1	22	-	2419	1	intergenic	novelGene_2050	31524693	31527111
PB.10233.1	23	+	2343	1	intergenic	novelGene_2094	359974	362316
PB.10233.2	23	+	3238	1	intergenic	novelGene_2096	359978	363215
PB.10233.3	23	+	2753	1	intergenic	novelGene_2095	359978	362730
PB.10234.1	23	+	2188	2	intergenic	novelGene_2097	552738	560830
PB.10242.1	23	+	1955	1	intergenic	novelGene_2098	2078381	2080335
PB.10243.1	23	-	4022	1	intergenic	novelGene_2100	2240583	2244604
PB.10243.2	23	-	1901	1	intergenic	novelGene_2099	2240583	2242483
PB.10244.1	23	+	3376	1	intergenic	novelGene_2101	2271260	2274635
PB.10245.1	23	+	3166	1	intergenic	novelGene_2102	2307939	2311104
PB.10254.1	23	+	1188	1	intergenic	novelGene_2103	4728285	4729472
PB.10257.1	23	-	1325	7	intergenic	novelGene_2104	4908436	4918397
PB.10260.1	23	-	1981	1	intergenic	novelGene_2105	5161858	5163838
PB.10261.1	23	+	2294	1	intergenic	novelGene_2106	5182482	5184775
PB.10270.1	23	-	1748	1	intergenic	novelGene_2107	8704385	8706132
PB.10282.1	23	+	2088	1	antisense	novelGene_ENSCHIG00000026737_AS	11003951	11006038
PB.10283.6	23	-	1484	1	intergenic	novelGene_2108	10980863	10982346
PB.10284.4	23	+	1611	1	intergenic	novelGene_2109	11095546	11097156
PB.10286.1	23	-	1965	1	intergenic	novelGene_2110	12820505	12822469
PB.10287.6	23	+	1364	1	intergenic	novelGene_2111	12949379	12950742
PB.10289.1	23	+	1742	1	intergenic	novelGene_2112	13841754	13843495
PB.10296.2	23	-	1094	2	intergenic	novelGene_2113	15216670	15218826
PB.10298.7	23	+	2293	1	intergenic	novelGene_2114	15583459	15585751
PB.10306.3	23	-	1568	1	intergenic	novelGene_2115	17041211	17042778
PB.10307.1	23	-	1399	1	intergenic	novelGene_2116	17176593	17177991
PB.10310.1	23	-	1224	1	intergenic	novelGene_2117	17651408	17652631
PB.10321.1	23	+	1608	3	intergenic	novelGene_2118	18908276	18911838
PB.10324.1	23	+	2591	1	intergenic	novelGene_2119	19391791	19394381
PB.10325.1	23	+	1494	2	intergenic	novelGene_2120	19417354	19421701
PB.10328.1	23	+	1488	3	intergenic	novelGene_2121	19847586	19851540
PB.10331.10	23	-	2191	7	intergenic	novelGene_2123	20930278	20934427
PB.10331.11	23	-	2104	7	intergenic	novelGene_2130	20930278	20934428
PB.10331.12	23	-	1991	7	intergenic	novelGene_2124	20930278	20934427
PB.10331.14	23	-	1945	7	intergenic	novelGene_2132	20930451	20934442
PB.10331.15	23	-	2041	7	intergenic	novelGene_2133	20930453	20934452
PB.10331.16	23	-	1494	7	intergenic	novelGene_2134	20930887	20934427
PB.10331.2	23	-	2319	7	intergenic	novelGene_2122	20930073	20934438

PB.10331.3	23	-	3603	3	intergenic	novelGene_2125	20930278	20934427
PB.10331.4	23	-	3006	4	intergenic	novelGene_2126	20930278	20934427
PB.10331.5	23	-	2728	5	intergenic	novelGene_2131	20930278	20934442
PB.10331.6	23	-	2655	5	intergenic	novelGene_2127	20930278	20934427
PB.10331.8	23	-	2384	6	intergenic	novelGene_2128	20930278	20934427
PB.10331.9	23	-	2326	6	intergenic	novelGene_2129	20930278	20934427
PB.10333.1	23	-	1536	2	intergenic	novelGene_2135	21151306	21155253
PB.10336.1	23	-	1808	1	intergenic	novelGene_2136	21220402	21222209
PB.10345.1	23	-	2940	2	intergenic	novelGene_2137	21511562	21522652
PB.10346.1	23	-	1220	1	intergenic	novelGene_2138	21557971	21559190
PB.10350.4	23	-	1475	2	intergenic	novelGene_2139	21615659	21617358
PB.10368.2	23	+	1609	1	intergenic	novelGene_2140	22110591	22112199
PB.10375.1	23	-	2449	1	intergenic	novelGene_2141	22323623	22326071
PB.10375.2	23	-	2008	1	intergenic	novelGene_2142	22324083	22326090
PB.10389.1	23	+	1658	4	intergenic	novelGene_2143	22459186	22461841
PB.10389.2	23	+	1299	5	intergenic	novelGene_2144	22459186	22461841
PB.10390.1	23	-	1974	1	intergenic	novelGene_2145	22464370	22466343
PB.10395.1	23	+	2833	18	intergenic	novelGene_2146	22553045	22559434
PB.10421.1	23	-	1698	1	intergenic	novelGene_2147	24374844	24376541
PB.10427.1	23	-	1231	1	intergenic	novelGene_2148	26863626	26864856
PB.10437.1	23	-	1801	1	intergenic	novelGene_2149	28924033	28925833
PB.10442.1	23	-	2457	1	intergenic	novelGene_2150	30940878	30943334
PB.10448.3	23	+	2730	1	intergenic	novelGene_2151	31193029	31195758
PB.10459.11	23	-	1435	1	intergenic	novelGene_2152	32114200	32115634
PB.10491.1	23	-	1462	1	intergenic	novelGene_2153	36486377	36487838
PB.10492.1	23	+	1384	1	intergenic	novelGene_2154	36857320	36858703
PB.10493.4	23	-	949	1	intergenic	novelGene_2155	36896145	36897093
PB.10493.5	23	-	1769	1	intergenic	novelGene_2156	36998169	36999937
PB.10501.4	23	-	1682	1	intergenic	novelGene_2157	37913952	37915633
PB.10504.1	23	+	1887	1	intergenic	novelGene_2158	38162806	38164692
PB.10504.2	23	+	1667	2	intergenic	novelGene_2159	38162863	38164691
PB.10507.1	23	+	931	1	intergenic	novelGene_2160	38224308	38225238
PB.10513.1	23	+	1297	1	intergenic	novelGene_2161	38697416	38698712
PB.10516.1	23	-	1952	1	intergenic	novelGene_2162	38986005	38987956
PB.10518.1	23	-	1452	1	intergenic	novelGene_2163	39122658	39124109
PB.10522.5	23	-	2008	1	intergenic	novelGene_2164	39351974	39353981
PB.10528.1	23	-	745	6	intergenic	novelGene_2165	39916315	39927133
PB.10529.1	23	+	2223	3	intergenic	novelGene_2166	39966745	40061629
PB.10574.1	24	+	3743	1	intergenic	novelGene_2167	587332	591074
PB.10577.1	24	+	1900	4	intergenic	novelGene_2168	1284822	1292071
PB.10578.4	24	+	3389	1	intergenic	novelGene_2169	2312566	2315954
PB.10579.1	24	+	3058	1	intergenic	novelGene_2170	2579534	2582591
PB.10580.1	24	+	1887	1	intergenic	novelGene_2171	2665812	2667698
PB.10581.1	24	-	2214	1	intergenic	novelGene_2172	3483463	3485676

PB.10584.7	24	-	1190	1	intergenic	novelGene_2173	4025539	4026728
PB.10585.4	24	+	2315	1	intergenic	novelGene_2174	4174300	4176614
PB.10592.1	24	+	1492	3	intergenic	novelGene_2175	13921294	13926307
PB.10601.1	24	+	2387	1	intergenic	novelGene_2176	21485153	21487539
PB.10606.1	24	+	2258	1	intergenic	novelGene_2177	22031079	22033336
PB.10610.1	24	-	2620	3	antisense	novelGene_ENSCHIG00000000359_AS	24685929	24691010
PB.10610.2	24	-	2998	4	antisense	novelGene_ENSCHIG00000000359_AS	24685930	24693941
PB.10611.1	24	-	2024	1	intergenic	novelGene_2178	25059751	25061774
PB.10618.8	24	-	1997	1	intergenic	novelGene_2179	26099666	26101662
PB.10621.11	24	+	2200	1	intergenic	novelGene_2180	26436123	26438322
PB.10622.1	24	+	1283	1	intergenic	novelGene_2181	26441893	26443175
PB.10626.2	24	+	2365	1	intergenic	novelGene_2182	31766028	31768392
PB.10626.3	24	+	2147	1	intergenic	novelGene_2183	31936682	31938828
PB.10629.1	24	-	1172	1	intergenic	novelGene_2184	32807642	32808813
PB.10636.1	24	+	2072	3	intergenic	novelGene_2185	33621981	33625431
PB.10638.1	24	-	2661	1	intergenic	novelGene_2186	34751422	34754082
PB.10639.1	24	+	1727	1	intergenic	novelGene_2187	34784815	34786541
PB.10652.1	24	-	2095	1	intergenic	novelGene_2188	37516518	37518612
PB.10653.1	24	+	1437	1	intergenic	novelGene_2189	37542074	37543510
PB.10657.3	24	+	1133	1	intergenic	novelGene_2190	37779725	37780857
PB.10659.1	24	+	1091	1	intergenic	novelGene_2191	37901280	37902370
PB.10660.1	24	+	2477	2	antisense	novelGene_ENSCHIG00000017982_AS	38012256	38026423
PB.10664.2	24	+	3400	1	intergenic	novelGene_2192	41433348	41436747
PB.10664.3	24	+	2020	1	intergenic	novelGene_2193	41434728	41436747
PB.10665.1	24	-	1789	1	antisense	novelGene_ENSCHIG00000023205_AS	41584732	41586520
PB.10666.1	24	+	3330	2	intergenic	novelGene_2194	41712030	41728812
PB.10676.3	24	+	4159	1	intergenic	novelGene_2200	43076698	43080856
PB.10676.4	24	+	2986	1	intergenic	novelGene_2199	43076698	43079683
PB.10676.5	24	+	2621	1	intergenic	novelGene_2198	43076698	43079318
PB.10676.6	24	+	2520	1	intergenic	novelGene_2197	43076698	43079217
PB.10676.7	24	+	1585	1	intergenic	novelGene_2196	43076698	43078282
PB.10676.8	24	+	1416	1	intergenic	novelGene_2195	43076698	43078113
PB.10689.1	24	+	1396	1	intergenic	novelGene_2201	43591267	43592662
PB.10692.4	24	+	1604	1	intergenic	novelGene_2202	43825240	43826843
PB.10694.1	24	+	2252	1	intergenic	novelGene_2203	44980950	44983201
PB.10709.1	24	+	2527	3	antisense	novelGene_ENSC	49660883	49690844

						HIG00000016585_AS		
PB.10709.2	24	+	2212	3	intergenic	novelGene_2204	49661161	49664672
PB.10715.1	24	-	1823	1	intergenic	novelGene_2205	50676144	50677966
PB.10722.1	24	-	2303	1	intergenic	novelGene_2206	54820840	54823142
PB.10723.1	24	+	1113	1	intergenic	novelGene_2207	54991039	54992151
PB.10724.1	24	+	2442	1	intergenic	novelGene_2208	55044355	55046796
PB.10725.1	24	+	1662	1	intergenic	novelGene_2209	55156178	55157839
PB.10726.1	24	+	2004	1	intergenic	novelGene_2210	55179320	55181323
PB.10729.2	24	+	1796	1	intergenic	novelGene_2211	56502594	56504389
PB.10733.1	24	+	1346	1	intergenic	novelGene_2212	57545633	57546978
PB.10737.1	24	+	2309	1	intergenic	novelGene_2213	58947544	58949852
PB.10737.2	24	+	1872	2	intergenic	novelGene_2214	58947544	58951762
PB.10740.1	24	-	2060	1	intergenic	novelGene_2215	61835790	61837849
PB.10745.1	24	-	2918	1	antisense	novelGene_ENSCHIG00000016340_AS	61999226	62002143
PB.10757.1	25	+	2522	1	intergenic	novelGene_2216	211851	214372
PB.10789.5	25	-	1150	9	antisense	novelGene_ENSCHIG00000007917_AS	1291899	1303802
PB.10789.7	25	-	1632	1	intergenic	novelGene_2217	1315114	1316745
PB.10795.1	25	+	794	2	intergenic	novelGene_2218	1480464	1481482
PB.10831.1	25	+	1462	1	intergenic	novelGene_2219	2654368	2655829
PB.10854.1	25	+	2078	1	antisense	novelGene_ENSCHIG00000023808_AS	3830913	3832990
PB.10856.1	25	+	1916	1	intergenic	novelGene_2220	3864513	3866428
PB.10866.1	25	+	1465	1	intergenic	novelGene_2221	7784017	7785481
PB.10866.2	25	+	2478	1	intergenic	novelGene_2222	7784065	7786542
PB.10871.1	25	-	2156	2	intergenic	novelGene_2223	9743929	9748679
PB.10874.1	25	+	2063	1	intergenic	novelGene_2224	10108291	10110353
PB.10875.1	25	+	4097	1	intergenic	novelGene_2225	10113857	10117953
PB.10876.8	25	-	2924	1	intergenic	novelGene_2226	10150291	10153214
PB.10881.8	25	-	1925	1	intergenic	novelGene_2227	10449061	10450985
PB.10882.1	25	-	2171	2	intergenic	novelGene_2228	10559509	10562796
PB.10885.4	25	+	1880	1	intergenic	novelGene_2230	12933044	12934923
PB.10886.1	25	-	2496	1	intergenic	novelGene_2229	12927322	12929817
PB.10887.1	25	+	1431	1	intergenic	novelGene_2231	13329812	13331242
PB.10889.4	25	+	3987	1	intergenic	novelGene_2232	13425542	13429528
PB.10899.1	25	+	1866	1	intergenic	novelGene_2233	15411355	15413220
PB.10902.2	25	-	1764	1	intergenic	novelGene_2234	16296695	16298458
PB.10902.3	25	-	3093	1	intergenic	novelGene_2235	16296697	16299789
PB.10903.6	25	-	2561	1	intergenic	novelGene_2236	16402153	16404713

PB.10904.1	25	-	1955	1	intergenic	novelGene_2237	16717228	16719182
PB.10916.7	25	-	1806	2	intergenic	novelGene_2238	18542198	18548503
PB.10923.1	25	+	1497	1	antisense	novelGene_ENSCHIG00000013287_AS	19948779	19950275
PB.10939.1	25	-	1098	1	intergenic	novelGene_2239	22160062	22161159
PB.10941.1	25	-	2653	1	antisense	novelGene_ENSCHIG00000019448_AS	22373572	22376224
PB.10961.1	25	-	3500	1	intergenic	novelGene_2240	25872154	25875653
PB.10990.1	25	+	986	2	intergenic	novelGene_2241	26455977	26457584
PB.11021.1	25	-	3563	1	intergenic	novelGene_2242	27367170	27370732
PB.11030.3	25	-	1649	1	intergenic	novelGene_2243	27618172	27619820
PB.11031.1	25	-	1992	3	intergenic	novelGene_2244	27622429	27628263
PB.11032.1	25	-	1667	1	intergenic	novelGene_2245	27703881	27705547
PB.11038.5	25	+	1746	2	intergenic	novelGene_2246	28051351	28053175
PB.11038.9	25	+	1823	1	intergenic	novelGene_2247	28051353	28053175
PB.11039.1	25	-	1736	1	intergenic	novelGene_2248	28069953	28071688
PB.11044.1	25	+	1164	1	intergenic	novelGene_2249	29802724	29803887
PB.11045.1	25	-	4049	1	intergenic	novelGene_2250	30476034	30480082
PB.11046.1	25	+	1874	1	intergenic	novelGene_2251	32460700	32462573
PB.11050.1	25	+	2322	1	antisense	novelGene_ENSCHIG00000008326_AS	32628894	32631215
PB.11051.16	25	-	3039	1	intergenic	novelGene_2252	32606130	32609168
PB.11062.4	25	+	2057	7	intergenic	novelGene_2253	33442789	33448115
PB.11067.3	25	-	1879	1	intergenic	novelGene_2254	33626266	33628144
PB.11068.3	25	-	2929	1	intergenic	novelGene_2255	33671230	33674158
PB.11071.1	25	+	2092	1	intergenic	novelGene_2256	33944902	33946993
PB.11071.2	25	+	3028	1	intergenic	novelGene_2257	33944927	33947954
PB.11071.3	25	+	3139	1	intergenic	novelGene_2258	33944936	33948074
PB.11084.5	25	-	2196	1	intergenic	novelGene_2259	35627003	35629198
PB.11090.1	25	+	2917	4	intergenic	novelGene_2260	36437944	36446426
PB.11093.1	25	-	2695	1	intergenic	novelGene_2261	36503002	36505696
PB.11102.1	25	+	1940	1	antisense	novelGene_ENSCHIG00000002627_AS	36779740	36781679
PB.11114.1	25	+	3418	6	intergenic	novelGene_2262	37178180	37191097
PB.11114.2	25	+	1207	6	intergenic	novelGene_2263	37178203	37188909
PB.11128.1	25	+	1397	1	intergenic	novelGene_2264	37724261	37725657
PB.11137.1	25	+	1985	1	antisense	novelGene_ENSCHIG00000015455_AS	37869326	37871310
PB.11142.1	25	+	2736	1	intergenic	novelGene_2265	38053469	38056204

PB.11143.1	25	-	1422	1	intergenic	novelGene_2266	38057755	38059176
PB.11152.4	25	-	2427	1	intergenic	novelGene_2267	38992580	38995006
PB.11159.1	25	+	1441	3	intergenic	novelGene_2268	39348674	39359161
PB.11160.1	25	+	2226	1	intergenic	novelGene_2269	39362490	39364715
PB.11167.1	25	-	372	1	antisense	novelGene_ENSCHIG00000013316_AS	39661582	39661953
PB.11170.1	25	+	3589	1	intergenic	novelGene_2270	39998171	40001759
PB.11170.2	25	+	2337	1	intergenic	novelGene_2271	39998176	40000512
PB.11172.1	25	-	1814	2	intergenic	novelGene_2272	40265910	40269188
PB.11174.1	25	+	1350	1	intergenic	novelGene_2273	41008711	41010060
PB.11175.1	25	+	1817	1	intergenic	novelGene_2274	41458822	41460638
PB.11176.1	25	+	1491	1	intergenic	novelGene_2275	41462544	41464034
PB.11185.1	25	-	2517	2	antisense	novelGene_ENSCHIG00000013183_AS	41820650	41824597
PB.11196.1	26	-	1847	2	intergenic	novelGene_2276	326638	329721
PB.11196.2	26	-	3089	1	intergenic	novelGene_2277	326639	329727
PB.11197.2	26	-	1921	3	intergenic	novelGene_2278	333368	339887
PB.11197.3	26	-	1975	3	intergenic	novelGene_2279	333368	339928
PB.11206.1	26	+	2276	1	intergenic	novelGene_2280	5416609	5418884
PB.11208.4	26	+	2826	1	intergenic	novelGene_2281	6533006	6535831
PB.11208.5	26	+	1724	1	intergenic	novelGene_2282	6533680	6535403
PB.11208.6	26	+	1659	1	intergenic	novelGene_2283	6534172	6535830
PB.11222.1	26	-	1282	1	intergenic	novelGene_2284	9176801	9178082
PB.11226.6	26	-	2599	1	intergenic	novelGene_2285	9704839	9707437
PB.11236.2	26	+	3402	1	intergenic	novelGene_2286	12017855	12021256
PB.11238.1	26	+	2335	1	intergenic	novelGene_2287	12229949	12232283
PB.11241.1	26	-	2860	1	intergenic	novelGene_2288	12398273	12401132
PB.11248.1	26	+	1912	1	intergenic	novelGene_2289	13043581	13045492
PB.11251.1	26	+	2019	1	intergenic	novelGene_2290	14304312	14306330
PB.11258.17	26	+	1577	1	intergenic	novelGene_2291	16993842	16995418
PB.11258.18	26	+	1742	1	intergenic	novelGene_2292	17012641	17014382
PB.11258.22	26	+	2909	1	intergenic	novelGene_2293	17075923	17078831
PB.11258.23	26	+	2130	1	intergenic	novelGene_2294	17077091	17079220
PB.11259.1	26	-	1966	1	antisense	novelGene_ENSCHIG00000013355_AS	17061540	17063505
PB.11266.1	26	-	1227	1	intergenic	novelGene_2295	17879665	17880891
PB.11267.1	26	+	1675	1	intergenic	novelGene_2296	18412992	18414666
PB.11268.1	26	+	1624	1	intergenic	novelGene_2297	18468267	18469890
PB.11273.1	26	+	1914	1	intergenic	novelGene_2298	19315345	19317258
PB.11275.1	26	+	1395	1	intergenic	novelGene_2299	20526512	20527906
PB.11282.1	26	+	1538	1	intergenic	novelGene_2300	21403012	21404549

PB.11285.1	26	+	2270	6	intergenic	novelGene_2301	23192361	23208355
PB.11289.2	26	+	3678	1	intergenic	novelGene_2302	27121359	27125036
PB.11290.1	26	-	1794	1	intergenic	novelGene_2303	27169472	27171265
PB.11294.1	26	+	2448	1	intergenic	novelGene_2304	27598813	27601260
PB.11298.1	26	+	2066	3	intergenic	novelGene_2305	27774811	27780514
PB.11298.2	26	+	384	4	intergenic	novelGene_2306	27774820	27780515
PB.11318.1	26	+	2161	1	antisense	novelGene_ENSCHIG00000011471_AS	28870194	28872354
PB.11334.1	26	-	2283	1	intergenic	novelGene_2307	30240806	30243088
PB.11354.1	26	+	2014	2	antisense	novelGene_ENSCHIG00000020686_AS	32623513	32629162
PB.11355.2	26	-	1781	1	intergenic	novelGene_2308	32610685	32612465
PB.11358.2	26	-	1937	3	intergenic	novelGene_2309	32742889	32747244
PB.11377.1	26	+	3429	1	intergenic	novelGene_2310	34405808	34409236
PB.11377.2	26	+	3020	1	intergenic	novelGene_2311	34406388	34409407
PB.11382.1	26	+	1640	1	intergenic	novelGene_2312	34766119	34767758
PB.11400.1	26	+	1758	1	antisense	novelGene_ENSCHIG00000007545_AS	37908651	37910408
PB.11403.3	26	-	3181	1	intergenic	novelGene_2313	38027027	38030207
PB.11405.1	26	-	2100	1	intergenic	novelGene_2314	38476155	38478254
PB.11411.1	26	+	1956	1	intergenic	novelGene_2315	40045173	40047128
PB.11418.2	26	-	1463	1	intergenic	novelGene_2316	40675184	40676646
PB.11426.2	26	-	1475	1	intergenic	novelGene_2317	42077497	42078971
PB.11428.9	26	+	1759	1	intergenic	novelGene_2318	42647789	42649547
PB.11430.1	26	-	1579	1	intergenic	novelGene_2319	43417567	43419145
PB.11438.1	27	+	2601	1	intergenic	novelGene_2320	3107330	3109930
PB.11444.1	27	+	2121	1	intergenic	novelGene_2321	5503748	5505868
PB.11447.2	27	+	2962	1	intergenic	novelGene_2322	6614174	6617135
PB.11449.1	27	+	3344	1	intergenic	novelGene_2323	7360362	7363705
PB.11455.4	27	-	1237	1	intergenic	novelGene_2324	7931818	7933054
PB.11460.1	27	-	1680	1	intergenic	novelGene_2325	8255936	8257615
PB.11465.1	27	-	2073	1	intergenic	novelGene_2326	8553348	8555420
PB.11467.1	27	+	791	1	intergenic	novelGene_2327	8768145	8768935
PB.11473.1	27	-	2341	2	intergenic	novelGene_2328	9496722	9499187
PB.11476.5	27	-	1616	1	intergenic	novelGene_2329	11570048	11571663
PB.11478.1	27	+	1398	1	intergenic	novelGene_2330	11600435	11601832
PB.11480.1	27	-	3131	1	intergenic	novelGene_2332	11714021	11717151
PB.11480.2	27	-	1910	1	intergenic	novelGene_2331	11714021	11715930
PB.11481.5	27	-	3073	1	intergenic	novelGene_2333	11808150	11811222
PB.11486.1	27	+	2170	1	intergenic	novelGene_2334	12283585	12285754
PB.11503.1	27	+	3282	1	antisense	novelGene_ENSC	17001189	17004470

						HIG00000002290_AS		
PB.11503.2	27	+	1617	1	antisense	novelGene_ENSCHIG00000002290_AS	17002854	17004470
PB.11504.4	27	-	1209	1	intergenic	novelGene_2335	17142862	17144070
PB.11509.4	27	-	3000	1	intergenic	novelGene_2336	19104806	19107805
PB.11515.2	27	-	2740	1	intergenic	novelGene_2337	20018003	20020742
PB.11521.9	27	-	1652	1	intergenic	novelGene_2338	21917878	21919529
PB.11532.1	27	-	825	1	intergenic	novelGene_2339	26387654	26388478
PB.11534.1	27	+	938	1	intergenic	novelGene_2340	29083983	29084920
PB.11547.1	27	-	2328	2	intergenic	novelGene_2341	30599641	30611069
PB.11554.1	27	-	1463	1	intergenic	novelGene_2342	36836971	36838433
PB.11558.1	27	+	3097	1	intergenic	novelGene_2343	37752227	37755323
PB.11561.1	27	+	2032	1	intergenic	novelGene_2344	38215806	38217837
PB.11564.1	27	+	385	2	intergenic	novelGene_2345	38583702	38591456
PB.11569.1	27	-	3027	1	intergenic	novelGene_2346	43184354	43187380
PB.11572.3	27	+	1477	1	intergenic	novelGene_2347	44105230	44106706
PB.11575.1	28	-	3046	5	intergenic	novelGene_2348	176646	191008
PB.11575.2	28	-	2821	5	intergenic	novelGene_2349	176862	190999
PB.11575.3	28	-	2153	1	intergenic	novelGene_2350	188807	190959
PB.11577.1	28	+	1973	1	intergenic	novelGene_2351	841816	843788
PB.11590.1	28	+	1523	6	intergenic	novelGene_2352	2312317	2343428
PB.11593.1	28	+	1689	1	intergenic	novelGene_2353	3034295	3035983
PB.11595.1	28	-	2344	2	intergenic	novelGene_2354	3511700	3521515
PB.11598.1	28	+	1578	2	intergenic	novelGene_2355	3937514	3947231
PB.11606.5	28	+	1498	1	intergenic	novelGene_2357	4487894	4489391
PB.11607.1	28	-	1707	1	intergenic	novelGene_2356	4486958	4488664
PB.11609.1	28	-	1114	3	intergenic	novelGene_2358	6553779	6562471
PB.11611.1	28	+	4187	1	intergenic	novelGene_2359	9924526	9928712
PB.11615.1	28	-	1557	2	antisense	novelGene_ENSCHIG00000000069_AS	10423565	10425713
PB.11625.1	28	-	1378	1	intergenic	novelGene_2360	12137380	12138757
PB.11626.1	28	-	2057	1	intergenic	novelGene_2361	13934946	13937002
PB.11627.1	28	-	2844	2	intergenic	novelGene_2362	14308615	14345373
PB.11633.4	28	-	2165	1	intergenic	novelGene_2363	15310849	15313013
PB.11635.1	28	+	1650	1	antisense	novelGene_ENSCHIG00000000321_AS	15658636	15660285
PB.11636.10	28	-	1581	1	intergenic	novelGene_2364	15645624	15647204
PB.11642.1	28	+	2455	1	intergenic	novelGene_2365	15957157	15959611
PB.11643.1	28	-	1882	1	intergenic	novelGene_2366	15968101	15969982
PB.11654.1	28	-	1937	1	intergenic	novelGene_2367	16517229	16519165

PB.11655.1	28	+	1514	1	intergenic	novelGene_2368	16864744	16866257
PB.11662.1	28	-	3112	2	antisense	novelGene_ENSCHIG00000020844_AS	17403304	17409716
PB.11662.2	28	-	2021	1	intergenic	novelGene_2369	17403306	17405326
PB.11664.1	28	-	4113	1	intergenic	novelGene_2370	17520389	17524501
PB.11669.9	28	-	1933	1	intergenic	novelGene_2371	18625665	18627597
PB.11672.1	28	-	1411	1	intergenic	novelGene_2372	19026389	19027799
PB.11672.7	28	-	2265	1	intergenic	novelGene_2373	19028221	19030485
PB.11682.1	28	+	2493	1	antisense	novelGene_ENSCHIG00000000496_AS	19937658	19940150
PB.11686.7	28	-	3329	1	intergenic	novelGene_2374	20173520	20176848
PB.11689.1	28	+	1326	1	intergenic	novelGene_2375	20419635	20420960
PB.11691.1	28	+	2311	1	intergenic	novelGene_2376	20459506	20461816
PB.11699.3	28	+	1411	1	intergenic	novelGene_2377	21162341	21163751
PB.11701.10	28	-	3251	1	intergenic	novelGene_2378	25947973	25951223
PB.11702.1	28	+	1461	1	intergenic	novelGene_2379	26161893	26163353
PB.11704.1	28	-	1128	1	intergenic	novelGene_2380	26293776	26294903
PB.11708.1	28	+	1347	1	intergenic	novelGene_2381	26678265	26679611
PB.11714.1	28	-	1781	1	intergenic	novelGene_2382	29333215	29334995
PB.11714.2	28	-	1639	1	intergenic	novelGene_2383	29333357	29334995
PB.11716.1	28	+	2029	1	intergenic	novelGene_2384	30057648	30059676
PB.11718.1	28	-	1966	1	intergenic	novelGene_2385	31349690	31351655
PB.11719.1	28	+	1748	1	intergenic	novelGene_2386	31550808	31552555
PB.11722.1	28	+	2350	1	intergenic	novelGene_2387	31938682	31941031
PB.11723.5	28	-	2769	1	intergenic	novelGene_2388	31991003	31993771
PB.11729.1	28	+	1676	1	intergenic	novelGene_2389	36209057	36210732
PB.11730.9	28	-	1519	2	intergenic	novelGene_2390	36210412	36214383
PB.11732.1	28	-	2279	1	antisense	novelGene_ENSCHIG00000019035_AS	36462102	36464380
PB.11734.2	28	+	1881	2	intergenic	novelGene_2391	36587966	36687270
PB.11735.1	28	+	3841	1	intergenic	novelGene_2392	36818138	36821978
PB.11736.1	28	+	1651	1	intergenic	novelGene_2393	36981576	36983226
PB.11745.1	28	-	1685	1	antisense	novelGene_ENSCHIG00000007684_AS	37471295	37472979
PB.11747.1	28	+	1769	1	intergenic	novelGene_2394	37963562	37965330
PB.11748.1	28	-	2626	2	antisense	novelGene_ENSCHIG00000001320_AS	38024124	38029616
PB.11748.2	28	-	2159	2	antisense	novelGene_ENSCHIG00000001320_	38024607	38029632

						AS		
PB.11750.2	28	-	2710	1	intergenic	novelGene_2395	39501137	39503846
PB.11751.4	28	-	288	1	intergenic	novelGene_2396	40990538	40990825
PB.11753.1	28	-	1767	1	intergenic	novelGene_2397	41237723	41239489
PB.11762.1	28	-	2910	1	intergenic	novelGene_2398	42465309	42468218
PB.11763.7	28	-	1647	1	intergenic	novelGene_2399	43003642	43005288
PB.11764.1	28	-	2383	4	intergenic	novelGene_2400	43090428	43128888
PB.11768.1	28	+	1349	2	intergenic	novelGene_2401	43894524	43895994
PB.11776.1	29	+	984	4	intergenic	novelGene_2402	803550	880820
PB.11777.1	29	-	1699	3	intergenic	novelGene_2403	4567569	4591368
PB.11782.1	29	-	1843	1	antisense	novelGene_ENSCHIG00000013901_AS	6812250	6814092
PB.11788.1	29	+	2934	1	intergenic	novelGene_2404	8686868	8689801
PB.11790.5	29	+	1483	1	intergenic	novelGene_2406	8980111	8981593
PB.11791.1	29	-	1492	1	intergenic	novelGene_2405	8971716	8973207
PB.11792.1	29	-	1241	1	antisense	novelGene_ENSCHIG00000015634_AS	9019624	9020864
PB.11793.2	29	+	3256	1	intergenic	novelGene_2407	9164028	9167283
PB.11793.3	29	+	1806	1	intergenic	novelGene_2408	9268481	9270286
PB.11795.1	29	+	1956	1	intergenic	novelGene_2409	11437490	11439445
PB.11801.1	29	+	2082	1	intergenic	novelGene_2410	12044084	12046165
PB.11802.1	29	-	2025	1	intergenic	novelGene_2411	12044662	12046686
PB.11815.1	29	-	1630	1	intergenic	novelGene_2412	17457672	17459301
PB.11820.1	29	+	2061	1	intergenic	novelGene_2413	17960090	17962150
PB.11873.1	29	-	1192	1	intergenic	novelGene_2414	35633164	35634355
PB.11892.1	29	-	1489	1	intergenic	novelGene_2415	40588784	40590272
PB.11902.1	29	+	2014	1	intergenic	novelGene_2416	41176956	41178969
PB.11904.1	29	+	1958	2	antisense	novelGene_ENSCHIG00000022487_AS	41242408	41249219
PB.11941.1	29	+	2671	1	antisense	novelGene_ENSCHIG00000015400_AS	42995515	42998185
PB.11954.1	29	-	2401	1	intergenic	novelGene_2417	43534095	43536495
PB.11974.1	29	+	2883	2	intergenic	novelGene_2418	44031981	44035606
PB.11974.14	29	+	1617	1	intergenic	novelGene_2427	44035810	44037426
PB.11974.2	29	+	5161	1	intergenic	novelGene_2425	44032266	44037426
PB.11974.3	29	+	3341	1	intergenic	novelGene_2424	44032266	44035606
PB.11974.4	29	+	3156	2	intergenic	novelGene_2422	44032266	44035603
PB.11974.5	29	+	2977	1	intergenic	novelGene_2421	44032266	44035242
PB.11974.6	29	+	2926	3	intergenic	novelGene_2423	44032266	44035603
PB.11974.7	29	+	2675	1	intergenic	novelGene_2420	44032266	44034940

PB.11974.8	29	+	1784	1	intergenic	novelGene_2419	44032266	44034049
PB.11974.9	29	+	2933	2	intergenic	novelGene_2426	44032368	44035605
PB.11975.1	29	+	3295	1	intergenic	novelGene_2428	44038298	44041592
PB.11975.2	29	+	1605	1	intergenic	novelGene_2429	44039988	44041592
PB.11976.1	29	+	4394	1	intergenic	novelGene_2430	44045398	44049791
PB.11976.2	29	+	3058	1	intergenic	novelGene_2431	44046734	44049791
PB.11976.3	29	+	1990	1	intergenic	novelGene_2432	44047802	44049791
PB.11976.4	29	+	955	1	intergenic	novelGene_2433	44048837	44049791
PB.11977.1	29	+	1037	1	intergenic	novelGene_2434	44052232	44053268
PB.11978.1	29	+	4385	1	intergenic	novelGene_2459	44090918	44095302
PB.11978.10	29	+	2412	1	intergenic	novelGene_2444	44090918	44093329
PB.11978.11	29	+	2198	1	intergenic	novelGene_2443	44090918	44093115
PB.11978.12	29	+	2088	2	intergenic	novelGene_2449	44090918	44094690
PB.11978.13	29	+	2081	2	intergenic	novelGene_2450	44090918	44094690
PB.11978.14	29	+	2067	2	intergenic	novelGene_2451	44090918	44094690
PB.11978.15	29	+	1966	2	intergenic	novelGene_2446	44090918	44094587
PB.11978.16	29	+	1753	1	intergenic	novelGene_2442	44090918	44092670
PB.11978.17	29	+	1648	2	intergenic	novelGene_2452	44090918	44094690
PB.11978.18	29	+	1491	2	intergenic	novelGene_2453	44090918	44094690
PB.11978.19	29	+	1455	1	intergenic	novelGene_2441	44090918	44092372
PB.11978.2	29	+	3821	1	intergenic	novelGene_2458	44090918	44094738
PB.11978.20	29	+	1172	1	intergenic	novelGene_2440	44090918	44092089
PB.11978.21	29	+	1171	2	intergenic	novelGene_2454	44090918	44094690
PB.11978.22	29	+	1001	1	intergenic	novelGene_2439	44090918	44091918
PB.11978.23	29	+	866	1	intergenic	novelGene_2438	44090918	44091783
PB.11978.24	29	+	720	1	intergenic	novelGene_2437	44090918	44091637
PB.11978.25	29	+	596	1	intergenic	novelGene_2436	44090918	44091513
PB.11978.26	29	+	209	1	intergenic	novelGene_2435	44090918	44091126
PB.11978.3	29	+	3688	1	intergenic	novelGene_2447	44090918	44094605
PB.11978.4	29	+	3405	2	intergenic	novelGene_2455	44090918	44094690
PB.11978.5	29	+	3395	2	intergenic	novelGene_2460	44090918	44097559
PB.11978.6	29	+	3216	2	intergenic	novelGene_2456	44090918	44094690
PB.11978.7	29	+	3129	2	intergenic	novelGene_2448	44090918	44094682
PB.11978.8	29	+	3051	2	intergenic	novelGene_2457	44090918	44094690
PB.11978.9	29	+	2836	1	intergenic	novelGene_2445	44090918	44093753
PB.11979.1	29	-	1065	1	intergenic	novelGene_2461	44091255	44092319
PB.11980.1	29	-	1826	1	intergenic	novelGene_2462	44094326	44096151
PB.11980.2	29	-	1504	1	intergenic	novelGene_2463	44095799	44097302
PB.11998.4	29	-	1854	1	antisense	novelGene_ENSCHIG00000001753_AS	44414369	44416222
PB.12004.1	29	+	2284	1	antisense	novelGene_ENSCHIG00000023526_AS	44534213	44536496

PB.12022.1	29	-	1861	4	intergenic	novelGene_2464	45005518	45008982
PB.12022.2	29	-	1758	4	intergenic	novelGene_2465	45005518	45009005
PB.12031.1	29	-	1513	1	antisense	novelGene_ENSCHIG00000018714_AS	45473268	45474780
PB.12032.1	29	+	2736	1	intergenic	novelGene_2466	45479355	45482090
PB.12087.1	29	+	1142	3	antisense	novelGene_ENSCHIG00000014907_AS	49318165	49322641
PB.12091.1	29	-	1871	2	antisense	novelGene_ENSCHIG00000016294_AS	49569032	49571405
PB.12097.1	29	+	3893	1	antisense	novelGene_ENSCHIG00000002335_AS	49933063	49936955
PB.12102.1	29	+	2672	2	intergenic	novelGene_2467	50438597	50441324
PB.12102.2	29	+	2355	2	intergenic	novelGene_2468	50438918	50441324
PB.12107.1	29	+	1155	1	antisense	novelGene_ENSCHIG00000015513_AS	50913852	50915006
PB.1095.1	3	-	709	2	intergenic	novelGene_289	137789	139212
PB.1096.1	3	-	638	2	intergenic	novelGene_290	248060	249422
PB.1097.2	3	-	689	2	intergenic	novelGene_291	270513	272156
PB.1098.2	3	-	2286	1	intergenic	novelGene_292	349097	351382
PB.1112.1	3	+	2996	1	antisense	novelGene_ENSCHIG00000016722_AS	1110165	1113160
PB.1114.3	3	-	2301	1	intergenic	novelGene_293	1127209	1129509
PB.1116.1	3	+	2210	3	antisense	novelGene_ENSCHIG00000020160_AS	1421526	1440968
PB.1120.1	3	-	1541	1	intergenic	novelGene_294	2984991	2986531
PB.1123.1	3	+	4346	1	intergenic	novelGene_295	3182216	3186561
PB.1123.2	3	+	1878	3	intergenic	novelGene_296	3182218	3186561
PB.1128.11	3	-	1303	1	intergenic	novelGene_297	3516026	3517328
PB.1128.16	3	-	2215	1	intergenic	novelGene_298	3517961	3520175
PB.1128.23	3	-	2668	1	intergenic	novelGene_299	3587702	3590369
PB.1130.1	3	+	4528	1	intergenic	novelGene_300	3863579	3868106
PB.1130.3	3	+	2239	1	intergenic	novelGene_301	3865868	3868106
PB.1134.1	3	-	3284	1	intergenic	novelGene_302	4727549	4730832
PB.1134.2	3	-	2182	1	intergenic	novelGene_303	4728652	4730833
PB.1134.3	3	-	1731	1	intergenic	novelGene_304	4729102	4730832
PB.1135.1	3	-	1843	1	intergenic	novelGene_305	5380694	5382536
PB.1146.1	3	+	1391	1	intergenic	novelGene_306	8283545	8284935

PB.1149.1	3	-	2034	1	intergenic	novelGene_307	8448110	8450143
PB.1151.2	3	+	1136	1	intergenic	novelGene_308	9893505	9894640
PB.1154.2	3	-	2189	1	intergenic	novelGene_309	9985506	9987694
PB.1178.1	3	-	1422	3	antisense	novelGene_ENSCHIG00000021957_AS	12390002	12397974
PB.1180.1	3	+	1449	1	intergenic	novelGene_310	12465817	12467265
PB.1182.9	3	-	2758	1	intergenic	novelGene_311	12482486	12485243
PB.1193.1	3	+	1820	2	intergenic	novelGene_312	12881797	12931895
PB.1199.1	3	+	2433	1	intergenic	novelGene_313	13905409	13907841
PB.1200.1	3	+	2176	1	intergenic	novelGene_314	13954976	13957151
PB.1201.1	3	+	1252	1	intergenic	novelGene_315	13960915	13962166
PB.1202.2	3	+	2528	1	intergenic	novelGene_316	14021028	14023555
PB.1205.1	3	-	1533	2	antisense	novelGene_ENSCHIG00000008154_AS	14181706	14183785
PB.1212.9	3	+	1903	1	intergenic	novelGene_317	14742132	14744034
PB.1225.1	3	-	1334	2	intergenic	novelGene_318	16610906	16659562
PB.1226.1	3	+	1508	1	intergenic	novelGene_319	16908308	16909815
PB.1266.2	3	-	2516	1	intergenic	novelGene_320	20192510	20195025
PB.1273.1	3	+	1624	1	intergenic	novelGene_321	20318474	20320097
PB.1284.1	3	+	2599	14	intergenic	novelGene_322	21056570	21081287
PB.1284.2	3	+	2005	15	intergenic	novelGene_324	21056573	21081287
PB.1284.3	3	+	1960	14	intergenic	novelGene_323	21056573	21081286
PB.1284.4	3	+	1859	14	intergenic	novelGene_325	21056573	21081287
PB.1284.5	3	+	1815	13	intergenic	novelGene_326	21056573	21081287
PB.1284.6	3	+	1498	11	intergenic	novelGene_327	21056573	21081287
PB.1284.7	3	+	1831	9	intergenic	novelGene_328	21066841	21081287
PB.1286.2	3	-	3530	1	intergenic	novelGene_329	21249094	21252623
PB.1290.7	3	+	1606	1	intergenic	novelGene_330	21706883	21708488
PB.1302.1	3	+	2284	2	intergenic	novelGene_331	26380190	26390402
PB.1303.3	3	-	1623	1	intergenic	novelGene_332	26520871	26522493
PB.1313.1	3	-	2295	1	intergenic	novelGene_333	27272318	27274612
PB.1316.1	3	+	2865	3	intergenic	novelGene_334	27579979	27588307
PB.1330.1	3	+	1746	1	antisense	novelGene_ENSCHIG00000013959_AS	29094808	29096553
PB.1333.8	3	-	1682	1	intergenic	novelGene_335	29338542	29340223
PB.1346.1	3	-	1519	7	intergenic	novelGene_336	34947307	34984503
PB.1346.2	3	-	1716	8	intergenic	novelGene_337	34947308	34984513
PB.1346.3	3	-	1908	9	intergenic	novelGene_338	34947309	34984502
PB.1346.4	3	-	1866	9	intergenic	novelGene_339	34947310	34984526
PB.1346.5	3	-	1684	8	intergenic	novelGene_340	34947310	34984532
PB.1347.6	3	+	1317	1	intergenic	novelGene_341	36330926	36332242

PB.1347.7	3	+	3131	1	intergenic	novelGene_342	36379442	36382572
PB.1348.1	3	+	3049	1	intergenic	novelGene_343	36646741	36649789
PB.1348.2	3	+	1516	1	intergenic	novelGene_344	36649776	36651291
PB.1349.1	3	+	1815	1	antisense	novelGene_ENSCHIG00000026504_AS	36841658	36843472
PB.1352.1	3	+	2761	3	intergenic	novelGene_348	37307513	37320587
PB.1352.2	3	+	2703	4	intergenic	novelGene_346	37307513	37320424
PB.1352.3	3	+	2598	3	intergenic	novelGene_347	37307513	37320424
PB.1352.4	3	+	2193	3	intergenic	novelGene_345	37307513	37320019
PB.1352.5	3	+	2743	2	intergenic	novelGene_349	37310195	37320424
PB.1356.1	3	-	1510	3	intergenic	novelGene_350	38679412	38681801
PB.1362.14	3	-	2288	1	intergenic	novelGene_351	40364708	40366995
PB.1362.19	3	-	2943	1	intergenic	novelGene_352	40582719	40585661
PB.1367.5	3	+	1592	1	intergenic	novelGene_353	42570006	42571597
PB.1371.1	3	+	1738	1	intergenic	novelGene_355	43302014	43303751
PB.1372.4	3	-	2054	1	intergenic	novelGene_354	43265236	43267289
PB.1372.5	3	-	1910	1	intergenic	novelGene_356	43400258	43402167
PB.1372.6	3	-	1682	1	intergenic	novelGene_357	43400505	43402186
PB.1382.1	3	-	2350	1	intergenic	novelGene_358	51779728	51782077
PB.1385.1	3	-	1703	1	intergenic	novelGene_359	52557543	52559245
PB.1389.1	3	+	1950	1	genic_intron	novelGene_360	54332447	54334396
PB.1390.1	3	+	3028	1	intergenic	novelGene_361	54403861	54406888
PB.1397.8	3	+	1730	1	intergenic	novelGene_362	58615149	58616878
PB.1401.1	3	-	2217	1	antisense	novelGene_ENSCHIG00000012566_AS	61587430	61589646
PB.1411.1	3	+	2493	1	intergenic	novelGene_363	63828077	63830569
PB.1422.1	3	-	1470	1	intergenic	novelGene_364	66919766	66921235
PB.1426.1	3	-	868	1	intergenic	novelGene_365	67363934	67364801
PB.1427.1	3	+	3293	1	intergenic	novelGene_366	68316574	68319866
PB.1427.2	3	+	1712	1	intergenic	novelGene_367	68318155	68319866
PB.1430.1	3	+	1341	1	intergenic	novelGene_369	69024977	69026317
PB.1431.7	3	-	3891	1	intergenic	novelGene_368	69020028	69023918
PB.1434.1	3	-	1915	1	intergenic	novelGene_370	69664269	69666183
PB.1454.4	3	-	1046	1	intergenic	novelGene_371	74377062	74378107
PB.1456.5	3	+	2942	1	intergenic	novelGene_372	76938017	76940958
PB.1462.1	3	+	1648	2	intergenic	novelGene_373	77413071	77418382
PB.1469.1	3	+	1903	1	intergenic	novelGene_374	78190945	78192847
PB.1471.1	3	+	1210	1	intergenic	novelGene_375	78244510	78245719
PB.1472.2	3	+	1226	1	intergenic	novelGene_376	78456623	78457848
PB.1475.1	3	+	448	1	antisense	novelGene_ENSCHIG00000025322_	81705567	81706014

						AS		
PB.1480.1	3	+	1417	1	intergenic	novelGene_377	85704705	85706121
PB.1511.1	3	+	1440	1	intergenic	novelGene_378	88297242	88298681
PB.1520.8	3	+	1431	1	intergenic	novelGene_379	88989048	88990478
PB.1523.3	3	+	1915	1	intergenic	novelGene_380	89713833	89715747
PB.1523.6	3	+	2148	1	intergenic	novelGene_381	89764124	89766271
PB.1531.1	3	+	2741	3	intergenic	novelGene_382	90938371	90942057
PB.1538.1	3	-	1290	1	intergenic	novelGene_383	91156584	91157873
PB.1539.1	3	+	3191	1	intergenic	novelGene_384	91195916	91199106
PB.1546.1	3	-	2418	1	intergenic	novelGene_385	92035921	92038338
PB.1547.2	3	-	1700	1	intergenic	novelGene_386	92040015	92041714
PB.1550.1	3	+	2264	1	intergenic	novelGene_387	92913028	92915291
PB.1553.1	3	-	2074	1	intergenic	novelGene_389	93614598	93616671
PB.1553.2	3	-	816	1	intergenic	novelGene_388	93614598	93615413
PB.1554.5	3	-	1242	6	intergenic	novelGene_390	93781667	93828782
PB.1558.1	3	-	1920	1	intergenic	novelGene_391	94232500	94234419
PB.1559.1	3	-	1946	1	intergenic	novelGene_392	94271649	94273594
PB.1564.1	3	-	1809	2	intergenic	novelGene_393	94696572	94699209
PB.1573.1	3	-	3193	1	intergenic	novelGene_394	97171973	97175165
PB.1589.2	3	-	1534	1	intergenic	novelGene_395	99085850	99087383
PB.1598.1	3	+	2569	1	antisense	novelGene_ENSCHIG00000021928_AS	99485417	99487985
PB.1614.1	3	-	1768	1	antisense	novelGene_ENSCHIG00000024595_AS	100018188	100019955
PB.1616.1	3	-	1521	1	intergenic	novelGene_396	100045049	100046569
PB.1629.1	3	-	1216	1	intergenic	novelGene_397	100443815	100445030
PB.1644.1	3	-	906	1	intergenic	novelGene_398	100935774	100936679
PB.1655.1	3	-	249	1	intergenic	novelGene_399	102049163	102049411
PB.1658.7	3	-	695	3	intergenic	novelGene_400	102148081	102163524
PB.1661.1	3	+	1120	1	intergenic	novelGene_401	102361878	102362997
PB.1661.2	3	+	749	2	intergenic	novelGene_402	102361878	102362997
PB.1662.1	3	+	1741	1	intergenic	novelGene_403	102365711	102367451
PB.1662.2	3	+	699	2	intergenic	novelGene_404	102365711	102367451
PB.1663.1	3	-	2533	1	intergenic	novelGene_405	102415760	102418292
PB.1663.2	3	-	609	2	intergenic	novelGene_406	102417169	102418292
PB.1665.1	3	+	1681	2	intergenic	novelGene_407	102519104	102521496
PB.1667.1	3	+	1608	1	intergenic	novelGene_408	102562096	102563703
PB.1667.2	3	+	693	2	intergenic	novelGene_409	102562096	102563734
PB.1668.1	3	+	1654	1	intergenic	novelGene_410	102631080	102632733
PB.1668.2	3	+	681	2	intergenic	novelGene_411	102631081	102632733
PB.1669.1	3	+	679	2	intergenic	novelGene_412	102637191	102639456
PB.1670.1	3	+	668	2	intergenic	novelGene_413	102648958	102650267

PB.1671.1	3	+	801	3	intergenic	novelGene_415	102762522	102764729
PB.1672.1	3	-	1950	3	intergenic	novelGene_414	102735470	102764512
PB.1675.1	3	+	932	3	intergenic	novelGene_416	102936506	102938359
PB.1675.2	3	+	1842	1	intergenic	novelGene_418	102936507	102938348
PB.1675.3	3	+	1172	2	intergenic	novelGene_419	102936507	102938348
PB.1675.4	3	+	1188	2	intergenic	novelGene_420	102936507	102938348
PB.1675.5	3	+	971	2	intergenic	novelGene_417	102936507	102938147
PB.1676.1	3	+	2475	1	intergenic	novelGene_422	102978750	102981224
PB.1676.2	3	+	2440	2	intergenic	novelGene_421	102978750	102981222
PB.1676.3	3	+	1255	2	intergenic	novelGene_423	102978750	102981224
PB.1681.1	3	-	2059	2	intergenic	novelGene_424	103101347	103105601
PB.1694.4	3	-	2011	1	intergenic	novelGene_425	103576690	103578700
PB.1726.2	3	-	1801	1	intergenic	novelGene_426	104763437	104765237
PB.1735.1	3	-	2251	2	intergenic	novelGene_427	105219227	105223667
PB.1751.1	3	-	2932	1	intergenic	novelGene_428	105758210	105761141
PB.1760.1	3	-	1267	2	intergenic	novelGene_429	106022117	106031934
PB.1765.1	3	+	2504	5	intergenic	novelGene_430	106967125	106986644
PB.1766.1	3	+	3098	6	intergenic	novelGene_431	107007796	107020067
PB.1766.2	3	+	3210	7	intergenic	novelGene_432	107007798	107020067
PB.1766.3	3	+	2980	6	intergenic	novelGene_433	107008406	107020067
PB.1767.1	3	+	1861	6	intergenic	novelGene_434	107069386	107112034
PB.1789.1	3	-	2718	1	intergenic	novelGene_435	110802265	110804982
PB.1810.1	3	+	2587	10	antisense	novelGene_ENSCHIG00000023854_AS	111841517	111845959
PB.1824.1	3	+	3113	1	intergenic	novelGene_436	113487929	113491041
PB.1837.1	3	-	2001	1	intergenic	novelGene_437	118921395	118923395
PB.1843.4	3	+	2589	1	intergenic	novelGene_438	119673153	119675741
PB.1847.1	4	-	3368	1	intergenic	novelGene_439	449044	452411
PB.1849.1	4	-	4318	1	intergenic	novelGene_440	1199595	1203912
PB.1851.1	4	+	1601	1	intergenic	novelGene_441	1266910	1268510
PB.1855.4	4	-	1069	1	intergenic	novelGene_442	2745309	2746377
PB.1884.1	4	-	2715	1	intergenic	novelGene_443	8088361	8091075
PB.1890.1	4	+	1448	1	intergenic	novelGene_445	8718530	8719977
PB.1891.2	4	-	1724	1	intergenic	novelGene_444	8683605	8685328
PB.1900.1	4	-	3988	3	intergenic	novelGene_446	14091918	14098409
PB.1900.2	4	-	543	4	intergenic	novelGene_447	14091918	14098409
PB.1900.3	4	-	1600	1	intergenic	novelGene_448	14094098	14095697
PB.1901.1	4	-	600	4	intergenic	novelGene_449	14112014	14122986
PB.1904.2	4	-	790	5	intergenic	novelGene_450	14517036	14522568
PB.1907.1	4	+	2106	1	intergenic	novelGene_451	15527745	15529850
PB.1919.1	4	+	3965	1	intergenic	novelGene_452	17372543	17376507
PB.1919.2	4	+	2580	1	intergenic	novelGene_453	17373928	17376507
PB.1921.1	4	+	1069	2	intergenic	novelGene_454	17519836	17525538

PB.1926.1	4	-	824	1	intergenic	novelGene_455	18804388	18805211
PB.1929.1	4	-	2281	1	intergenic	novelGene_456	20624870	20627150
PB.1932.1	4	-	1985	3	intergenic	novelGene_458	20920941	20936711
PB.1932.2	4	-	2157	3	intergenic	novelGene_457	20920941	20936307
PB.1932.4	4	-	1575	3	intergenic	novelGene_459	20921360	20936720
PB.1932.5	4	-	1404	3	intergenic	novelGene_460	20921515	20936704
PB.1932.6	4	-	1191	3	intergenic	novelGene_461	20921778	20936754
PB.1932.7	4	-	1022	3	intergenic	novelGene_462	20921925	20936732
PB.1932.8	4	-	769	3	intergenic	novelGene_463	20922176	20936730
PB.1937.26	4	-	2381	1	intergenic	novelGene_464	21707560	21709940
PB.1937.27	4	-	1242	1	intergenic	novelGene_465	21724597	21725838
PB.1943.1	4	+	5666	8	intergenic	novelGene_466	24887728	24939421
PB.1944.1	4	+	1253	1	intergenic	novelGene_467	25065909	25067161
PB.1946.1	4	+	2202	2	intergenic	novelGene_468	25466956	25488760
PB.1947.1	4	+	1836	2	intergenic	novelGene_469	25518025	25535519
PB.1953.1	4	+	2781	1	intergenic	novelGene_470	26396582	26399362
PB.1954.1	4	+	2102	1	intergenic	novelGene_471	26422372	26424473
PB.1957.3	4	-	1986	1	intergenic	novelGene_472	26944285	26946270
PB.1968.11	4	-	1785	1	intergenic	novelGene_473	28052505	28054289
PB.1972.1	4	-	2173	1	intergenic	novelGene_474	28514886	28517058
PB.1980.1	4	+	1122	1	intergenic	novelGene_475	33674552	33675673
PB.1985.1	4	-	1537	8	intergenic	novelGene_476	37490408	37513924
PB.1987.1	4	+	1782	1	intergenic	novelGene_477	38060044	38061825
PB.1991.1	4	+	1402	1	intergenic	novelGene_478	39147343	39148744
PB.2003.1	4	-	2746	1	intergenic	novelGene_479	42971232	42973977
PB.2014.3	4	-	656	5	intergenic	novelGene_480	43566219	43569270
PB.2018.1	4	-	2290	3	antisense	novelGene_ENSCHIG00000015588_AS	44026655	44046160
PB.2025.11	4	+	2890	1	intergenic	novelGene_484	47803576	47806465
PB.2025.15	4	+	1845	1	intergenic	novelGene_483	47803576	47805420
PB.2025.6	4	+	1515	1	intergenic	novelGene_481	47803574	47805088
PB.2025.8	4	+	1908	3	intergenic	novelGene_482	47803575	47806088
PB.2029.1	4	+	3093	1	intergenic	novelGene_485	49349154	49352246
PB.2033.1	4	+	1124	1	intergenic	novelGene_486	50489946	50491069
PB.2044.1	4	+	2637	1	intergenic	novelGene_487	52216524	52219160
PB.2046.1	4	+	1215	1	intergenic	novelGene_488	52482471	52483685
PB.2056.1	4	-	1752	1	antisense	novelGene_ENSCHIG00000016912_AS	54501534	54503285
PB.2067.10	4	+	2026	1	intergenic	novelGene_489	58912238	58914263
PB.2076.1	4	-	1316	1	intergenic	novelGene_490	64123047	64124362
PB.2078.1	4	-	2584	1	antisense	novelGene_ENSCHIG00000017336_	64313056	64315639

						AS		
PB.2079.1	4	+	1291	1	intergenic	novelGene_491	64344449	64345739
PB.2081.1	4	+	2052	1	intergenic	novelGene_492	64680177	64682228
PB.2083.1	4	-	1592	3	intergenic	novelGene_493	64891190	64893596
PB.2083.2	4	-	987	3	intergenic	novelGene_494	64891798	64893599
PB.2086.5	4	+	1419	1	intergenic	novelGene_495	68102779	68104197
PB.2099.1	4	-	2241	1	intergenic	novelGene_496	70789124	70791364
PB.2131.1	4	-	2043	1	intergenic	novelGene_497	79235570	79237612
PB.2132.1	4	-	2010	1	intergenic	novelGene_498	79329112	79331121
PB.2133.2	4	+	1579	1	intergenic	novelGene_499	79810672	79812250
PB.2137.1	4	+	1511	1	intergenic	novelGene_500	82154290	82155800
PB.2138.1	4	+	3292	1	intergenic	novelGene_501	84974649	84977940
PB.2144.1	4	+	1767	9	intergenic	novelGene_502	88442539	88461495
PB.2144.2	4	+	5450	1	intergenic	novelGene_503	88442561	88448010
PB.2144.3	4	+	3899	1	intergenic	novelGene_504	88442570	88446468
PB.2144.4	4	+	2043	10	intergenic	novelGene_505	88442570	88461495
PB.2144.5	4	+	1944	9	intergenic	novelGene_506	88442579	88461495
PB.2144.6	4	+	1835	8	intergenic	novelGene_507	88442579	88461693
PB.2144.7	4	+	1636	8	intergenic	novelGene_508	88442580	88461495
PB.2145.1	4	-	1781	1	intergenic	novelGene_509	88443581	88445361
PB.2151.1	4	+	454	3	intergenic	novelGene_510	89058282	89066187
PB.2153.1	4	+	2700	1	intergenic	novelGene_511	89557291	89559990
PB.2159.1	4	-	2059	1	antisense	novelGene_ENSCHIG00000014483_AS	94078495	94080553
PB.2161.1	4	-	1277	1	intergenic	novelGene_512	94675669	94676945
PB.2162.2	4	-	1514	1	intergenic	novelGene_513	94722980	94724493
PB.2169.1	4	-	1357	1	intergenic	novelGene_514	99779804	99781160
PB.2178.1	4	+	1630	1	antisense	novelGene_ENSCHIG00000025282_AS	105355833	105357462
PB.2182.1	4	+	464	3	intergenic	novelGene_515	106593681	106617348
PB.2196.2	4	+	1482	1	intergenic	novelGene_516	110682213	110683694
PB.2199.3	4	-	1580	1	intergenic	novelGene_517	111031990	111033569
PB.2208.1	4	-	1875	1	intergenic	novelGene_518	112938738	112940612
PB.2214.1	4	-	3535	1	intergenic	novelGene_519	116008403	116011937
PB.2214.2	4	-	2088	1	intergenic	novelGene_520	116009904	116011991
PB.2215.3	5	+	1612	1	intergenic	novelGene_521	940700	942311
PB.2221.2	5	+	2065	1	intergenic	novelGene_522	3857847	3859911
PB.2223.2	5	-	1279	1	antisense	novelGene_ENSCHIG00000024224_AS	4813075	4814353
PB.2225.1	5	+	2292	1	intergenic	novelGene_523	5381137	5383428
PB.2227.3	5	-	1651	1	intergenic	novelGene_524	5678713	5680363

PB.2230.1	5	+	2636	3	intergenic	novelGene_525	6571148	6644156
PB.2230.2	5	+	2521	2	intergenic	novelGene_526	6571179	6644156
PB.2240.3	5	-	5322	1	intergenic	novelGene_527	19164278	19169599
PB.2240.4	5	-	2386	1	intergenic	novelGene_528	19167204	19169589
PB.2240.5	5	-	2156	1	intergenic	novelGene_529	19167451	19169606
PB.2242.1	5	+	1316	1	intergenic	novelGene_530	19359507	19360822
PB.2246.25	5	-	1253	1	intergenic	novelGene_531	20851586	20852838
PB.2247.1	5	-	1980	4	intergenic	novelGene_532	20879721	20919630
PB.2248.1	5	+	1368	1	intergenic	novelGene_533	21755714	21757081
PB.2256.1	5	+	1772	1	intergenic	novelGene_534	24138367	24140138
PB.2260.3	5	+	4357	1	intergenic	novelGene_535	24645706	24650062
PB.2261.1	5	+	2012	1	intergenic	novelGene_536	24729735	24731746
PB.2264.1	5	-	623	4	antisense	novelGene_ENSCHIG00000001467_AS	25039241	25043162
PB.2264.2	5	-	2607	2	antisense	novelGene_ENSCHIG00000001467_AS	25039242	25043162
PB.2275.1	5	+	2644	1	intergenic	novelGene_537	25557097	25559740
PB.2275.2	5	+	1124	1	intergenic	novelGene_538	25558617	25559740
PB.2294.2	5	+	2326	1	intergenic	novelGene_539	26554680	26557005
PB.2307.1	5	-	2301	1	antisense	novelGene_ENSCHIG00000026933_AS	26962758	26965058
PB.2308.1	5	+	2327	9	intergenic	novelGene_540	26987908	26995855
PB.2308.4	5	+	1059	3	intergenic	novelGene_541	26993559	26995855
PB.2338.1	5	+	1949	1	intergenic	novelGene_542	28364669	28366617
PB.2338.2	5	+	1686	2	intergenic	novelGene_543	28364747	28366618
PB.2342.1	5	-	1419	1	intergenic	novelGene_544	28833701	28835119
PB.2343.1	5	+	1838	1	intergenic	novelGene_545	29056417	29058254
PB.2344.1	5	-	1459	1	intergenic	novelGene_546	29063334	29064792
PB.2349.3	5	-	1622	1	intergenic	novelGene_547	29450687	29452308
PB.2357.1	5	+	1788	1	intergenic	novelGene_548	29981293	29983080
PB.2366.1	5	+	2754	2	intergenic	novelGene_549	30494798	30512335
PB.2366.2	5	+	1649	1	intergenic	novelGene_550	30494801	30496449
PB.2366.5	5	+	2695	2	intergenic	novelGene_551	30494819	30512341
PB.2372.1	5	-	2127	1	antisense	novelGene_ENSCHIG00000024236_AS	30714841	30716967
PB.2373.1	5	+	2405	1	intergenic	novelGene_552	30728475	30730879
PB.2386.1	5	-	3138	1	antisense	novelGene_ENSCHIG00000010302_AS	33400846	33403983
PB.2386.2	5	-	2919	3	antisense	novelGene_ENSC	33400846	33403983

						HIG00000010302_ AS		
PB.2389.1	5	-	1736	1	intergenic	novelGene_553	33764022	33765757
PB.2390.1	5	+	997	1	intergenic	novelGene_554	33974630	33975626
PB.2391.1	5	+	3763	1	intergenic	novelGene_556	34055804	34059566
PB.2391.2	5	+	1796	1	intergenic	novelGene_555	34055804	34057599
PB.2392.1	5	-	1573	1	intergenic	novelGene_557	34423577	34425149
PB.2393.7	5	-	1626	2	intergenic	novelGene_558	34645421	34650629
PB.2403.1	5	-	1747	1	intergenic	novelGene_559	40516616	40518362
PB.2411.2	5	-	3364	1	intergenic	novelGene_560	43484329	43487692
PB.2411.3	5	-	1844	1	intergenic	novelGene_561	43485831	43487674
PB.2420.12	5	-	2144	1	intergenic	novelGene_562	44568818	44570961
PB.2427.1	5	+	1545	1	intergenic	novelGene_563	47767403	47768947
PB.2434.1	5	+	2223	1	intergenic	novelGene_564	48761059	48763281
PB.2435.13	5	-	2834	3	antisense	novelGene_ENSC HIG00000014883_ AS	48773304	48834147
PB.2450.1	5	-	1663	3	intergenic	novelGene_565	55063052	55064900
PB.2462.1	5	+	967	4	intergenic	novelGene_566	55533703	55535974
PB.2466.1	5	-	2313	3	intergenic	novelGene_568	55646579	55653302
PB.2466.2	5	-	2129	2	intergenic	novelGene_567	55646579	55652699
PB.2479.4	5	+	1643	1	intergenic	novelGene_569	56371279	56372921
PB.2492.1	5	-	1365	2	intergenic	novelGene_570	56580244	56581722
PB.2494.1	5	+	2385	1	intergenic	novelGene_571	56613281	56615665
PB.2506.8	5	+	1109	1	intergenic	novelGene_572	56815567	56816675
PB.2508.1	5	-	1879	1	antisense	novelGene_ENSC HIG00000013335_ AS	56821408	56823286
PB.2518.1	5	-	2786	1	intergenic	novelGene_573	59163862	59166647
PB.2519.3	5	-	2841	1	intergenic	novelGene_574	59341231	59344071
PB.2527.1	5	-	1974	1	intergenic	novelGene_575	62912056	62914029
PB.2532.10	5	+	2120	2	intergenic	novelGene_576	64055577	64077303
PB.2532.14	5	+	2063	1	intergenic	novelGene_577	64124938	64127000
PB.2538.2	5	+	1696	1	intergenic	novelGene_578	64618096	64619791
PB.2541.1	5	-	1427	10	antisense	novelGene_ENSC HIG00000009693_ AS	66292267	66303332
PB.2542.2	5	+	1654	1	intergenic	novelGene_579	66323977	66325630
PB.2546.3	5	+	1512	1	intergenic	novelGene_580	66608756	66610267
PB.2549.1	5	-	2000	1	intergenic	novelGene_581	67236597	67238596
PB.2553.1	5	+	1323	6	intergenic	novelGene_582	67513105	67558417
PB.2559.4	5	-	2350	1	intergenic	novelGene_583	68946884	68949233
PB.2567.1	5	+	1820	1	intergenic	novelGene_584	72224869	72226688
PB.2573.1	5	+	2377	1	intergenic	novelGene_585	72575817	72578193

PB.2574.8	5	-	2664	1	intergenic	novelGene_586	72854474	72857137
PB.2577.1	5	-	2519	4	intergenic	novelGene_587	73190309	73199737
PB.2580.28	5	-	2715	1	intergenic	novelGene_588	73455277	73457991
PB.2580.29	5	-	6168	1	intergenic	novelGene_590	73495718	73501885
PB.2580.30	5	-	1963	1	intergenic	novelGene_589	73495718	73497680
PB.2580.31	5	-	2925	1	intergenic	novelGene_591	73498992	73501916
PB.2608.1	5	+	3227	1	intergenic	novelGene_592	78883856	78887082
PB.2611.1	5	+	1780	1	intergenic	novelGene_593	80174116	80175895
PB.2615.3	5	-	1599	1	intergenic	novelGene_594	80715138	80716736
PB.2616.6	5	-	1721	1	intergenic	novelGene_595	81230544	81232264
PB.2623.1	5	-	1592	1	intergenic	novelGene_596	82008462	82010053
PB.2625.1	5	-	1562	1	intergenic	novelGene_597	83390930	83392491
PB.2626.1	5	+	2047	1	intergenic	novelGene_598	83410181	83412227
PB.2629.1	5	-	1175	1	intergenic	novelGene_599	84449586	84450760
PB.2632.1	5	-	2626	1	antisense	novelGene_ENSCHIG00000011005_AS	86261825	86264450
PB.2641.1	5	+	2427	1	antisense	novelGene_ENSCHIG00000025977_AS	88082005	88084431
PB.2642.1	5	-	1242	1	intergenic	novelGene_600	89020181	89021422
PB.2643.1	5	+	1754	1	intergenic	novelGene_601	89209444	89211197
PB.2644.1	5	+	2740	3	antisense	novelGene_ENSCHIG00000022500_AS	89407457	89416627
PB.2646.1	5	+	1786	1	intergenic	novelGene_602	89957322	89959107
PB.2648.4	5	-	4062	1	intergenic	novelGene_603	92111263	92115324
PB.2657.2	5	+	2005	1	intergenic	novelGene_604	93703278	93705282
PB.2661.14	5	-	2229	2	intergenic	novelGene_605	95278485	95280753
PB.2667.4	5	-	1744	1	intergenic	novelGene_606	95689976	95691719
PB.2669.1	5	+	1522	4	intergenic	novelGene_607	95750239	95753303
PB.2671.1	5	+	1430	1	intergenic	novelGene_608	95896973	95898402
PB.2676.1	5	+	1413	1	intergenic	novelGene_609	96793280	96794692
PB.2677.1	5	+	1382	1	intergenic	novelGene_610	96821636	96823017
PB.2680.9	5	+	1860	1	intergenic	novelGene_612	97563377	97565236
PB.2681.1	5	-	1378	1	intergenic	novelGene_611	97548692	97550069
PB.2688.1	5	-	2585	1	intergenic	novelGene_613	98615532	98618116
PB.2691.1	5	+	894	3	intergenic	novelGene_614	98941438	99019387
PB.2698.1	5	-	1822	1	intergenic	novelGene_615	99816570	99818391
PB.2705.1	5	+	2063	1	intergenic	novelGene_616	102170774	102172836
PB.2723.1	5	-	1150	5	intergenic	novelGene_617	102597983	102602743
PB.2723.2	5	-	1088	4	intergenic	novelGene_618	102597983	102602743
PB.2723.3	5	-	1038	6	intergenic	novelGene_619	102597990	102602743
PB.2744.1	5	+	1836	1	intergenic	novelGene_620	104412322	104414157

PB.2749.2	5	-	3255	1	intergenic	novelGene_623	104680843	104684097
PB.2749.3	5	-	2179	1	intergenic	novelGene_622	104680843	104683021
PB.2749.4	5	-	987	1	intergenic	novelGene_621	104680843	104681829
PB.2764.1	5	-	1302	1	antisense	novelGene_ENSCHIG00000013662_AS	106597496	106598797
PB.2768.1	5	+	2799	1	intergenic	novelGene_624	106974163	106976961
PB.2768.2	5	+	1610	1	intergenic	novelGene_625	106975348	106976957
PB.2776.11	5	+	2352	1	intergenic	novelGene_627	108006787	108009138
PB.2777.1	5	-	2367	1	intergenic	novelGene_626	108005062	108007428
PB.2785.1	5	-	1693	1	antisense	novelGene_ENSCHIG00000014332_AS	108378969	108380661
PB.2790.1	5	+	2260	1	intergenic	novelGene_628	108488821	108491080
PB.2798.1	5	+	2674	2	intergenic	novelGene_629	108746309	108749358
PB.2806.1	5	+	1805	1	intergenic	novelGene_630	109213399	109215203
PB.2808.1	5	+	2002	1	intergenic	novelGene_631	109253942	109255943
PB.2811.13	5	-	697	1	intergenic	novelGene_632	109299024	109299720
PB.2816.1	5	-	2263	1	intergenic	novelGene_633	109514420	109516682
PB.2826.1	5	+	2102	1	intergenic	novelGene_634	110580409	110582510
PB.2829.4	5	-	4640	2	intergenic	novelGene_635	110774513	110855743
PB.2829.5	5	-	2660	2	intergenic	novelGene_636	110819164	110855743
PB.2830.1	5	+	1638	1	intergenic	novelGene_637	110985072	110986709
PB.2835.5	5	-	1997	1	intergenic	novelGene_638	111357176	111359172
PB.2837.6	5	-	2068	1	intergenic	novelGene_639	111481179	111483246
PB.2843.4	5	-	1705	1	intergenic	novelGene_640	111613848	111615552
PB.2870.2	5	-	1633	1	intergenic	novelGene_641	114048445	114050077
PB.2877.1	5	-	1781	1	intergenic	novelGene_642	115063063	115064843
PB.2882.1	5	+	2058	2	intergenic	novelGene_643	115842025	115852276
PB.2883.1	5	-	3379	2	intergenic	novelGene_644	115855382	115859416
PB.2883.2	5	-	3305	2	intergenic	novelGene_645	115855387	115859422
PB.2883.3	5	-	3213	2	intergenic	novelGene_646	115855498	115859441
PB.2892.1	5	+	1912	1	antisense	novelGene_ENSCHIG00000017017_AS	118570820	118572731
PB.2892.2	5	+	1553	2	antisense	novelGene_ENSCHIG00000017017_AS	118570820	118572731
PB.2908.1	6	+	2133	1	intergenic	novelGene_647	2414084	2416216
PB.2915.1	6	+	2634	1	intergenic	novelGene_648	4740504	4743137
PB.2917.1	6	-	2324	4	antisense	novelGene_ENSCHIG00000022035_AS	6337316	6440178
PB.2932.1	6	-	781	1	intergenic	novelGene_649	15643719	15644499

PB.2938.1	6	+	1663	1	intergenic	novelGene_650	16165238	16166900
PB.2944.1	6	-	1548	1	intergenic	novelGene_651	17514325	17515872
PB.2955.7	6	-	1696	1	intergenic	novelGene_652	20334475	20336170
PB.2961.1	6	-	2155	1	antisense	novelGene_ENSCHIG00000025487_AS	22316036	22318190
PB.2964.1	6	-	1975	1	intergenic	novelGene_653	22505822	22507796
PB.2965.1	6	-	3372	1	intergenic	novelGene_654	22533514	22536885
PB.2966.1	6	-	2063	1	intergenic	novelGene_655	22544374	22546436
PB.2967.1	6	-	1743	1	intergenic	novelGene_656	22579653	22581395
PB.2971.1	6	-	1342	1	intergenic	novelGene_657	23724358	23725699
PB.2978.1	6	-	2075	1	antisense	novelGene_ENSCHIG00000023887_AS	25052677	25054751
PB.2988.10	6	-	1398	1	intergenic	novelGene_659	30533665	30535062
PB.2988.8	6	-	1184	1	intergenic	novelGene_658	30448992	30450175
PB.3003.2	6	-	3008	2	intergenic	novelGene_660	37853997	37857934
PB.3016.1	6	+	1818	1	intergenic	novelGene_661	46318861	46320678
PB.3032.9	6	-	1659	1	intergenic	novelGene_662	59280693	59282351
PB.3034.1	6	-	1193	1	intergenic	novelGene_663	59373597	59374789
PB.3038.1	6	+	1385	1	intergenic	novelGene_664	59749369	59750753
PB.3039.2	6	+	2000	1	intergenic	novelGene_665	59796820	59798819
PB.3041.1	6	-	3203	5	antisense	novelGene_ENSCHIG00000010597_AS	60482489	60665126
PB.3054.1	6	-	1453	1	intergenic	novelGene_666	67526104	67527556
PB.3056.1	6	-	2319	1	intergenic	novelGene_667	67909756	67912074
PB.3056.2	6	-	1724	3	intergenic	novelGene_668	67909756	67978322
PB.3056.3	6	-	2942	1	intergenic	novelGene_669	67957306	67960247
PB.3063.1	6	+	3684	4	intergenic	novelGene_670	68924932	68935096
PB.3063.2	6	+	3168	4	intergenic	novelGene_671	68924932	68936160
PB.3063.3	6	+	2103	4	intergenic	novelGene_672	68924934	68935097
PB.3068.1	6	+	1422	1	intergenic	novelGene_673	70088135	70089556
PB.3080.4	6	-	2448	1	intergenic	novelGene_674	72592217	72594664
PB.3082.1	6	+	2346	1	intergenic	novelGene_675	72785979	72788324
PB.3086.1	6	-	1346	1	intergenic	novelGene_676	72994903	72996248
PB.3087.2	6	-	1562	1	intergenic	novelGene_677	73041165	73042726
PB.3089.1	6	-	2756	1	intergenic	novelGene_678	77378829	77381584
PB.3090.1	6	+	2056	1	intergenic	novelGene_679	77383880	77385935
PB.3096.6	6	-	2485	1	intergenic	novelGene_680	86586252	86588736
PB.3101.1	6	+	1298	2	intergenic	novelGene_681	87806794	87808637
PB.3102.1	6	+	1821	12	antisense	novelGene_ENSCHIG00000008470_AS	87994251	88255001

PB.3132.1	6	-	3765	1	intergenic	novelGene_682	92884757	92888521
PB.3139.1	6	+	1749	1	antisense	novelGene_ENSCHIG00000010260_AS	97671647	97673395
PB.3139.2	6	+	1449	2	antisense	novelGene_ENSCHIG00000010260_AS	97671647	97673395
PB.3144.1	6	+	2252	1	intergenic	novelGene_683	98172720	98174971
PB.3146.1	6	-	2179	6	antisense	novelGene_ENSCHIG00000016593_AS	98175083	98183905
PB.3146.2	6	-	1888	3	intergenic	novelGene_684	98175083	98183487
PB.3146.3	6	-	1610	6	intergenic	novelGene_685	98175083	98183487
PB.3152.1	6	-	1744	1	intergenic	novelGene_686	99846145	99847888
PB.3153.1	6	+	1507	1	intergenic	novelGene_687	100152182	100153688
PB.3155.2	6	+	3342	1	intergenic	novelGene_688	101929032	101932373
PB.3155.4	6	+	1455	1	intergenic	novelGene_689	102056085	102057539
PB.3158.1	6	-	2741	1	antisense	novelGene_ENSCHIG00000008450_AS	102353525	102356265
PB.3164.1	6	-	1444	1	intergenic	novelGene_690	103763468	103764911
PB.3167.1	6	-	1491	1	intergenic	novelGene_691	104891564	104893054
PB.3176.1	6	-	1619	1	intergenic	novelGene_692	110440873	110442491
PB.3177.1	6	+	1453	1	intergenic	novelGene_693	110468812	110470264
PB.3181.1	6	+	2935	2	intergenic	novelGene_694	111135441	111143646
PB.3181.2	6	+	2128	1	intergenic	novelGene_695	111135449	111137576
PB.3181.3	6	+	544	3	intergenic	novelGene_696	111135453	111141159
PB.3204.1	6	+	1489	2	antisense	novelGene_ENSCHIG00000018283_AS	115967945	115970756
PB.3206.2	6	-	3044	2	intergenic	novelGene_698	116174982	116188703
PB.3206.3	6	-	2711	1	intergenic	novelGene_697	116174982	116177692
PB.3209.1	6	-	2180	2	intergenic	novelGene_699	116597040	116603933
PB.3210.3	6	+	3104	1	intergenic	novelGene_700	116625114	116628217
PB.3211.1	6	-	1597	1	intergenic	novelGene_701	116648582	116650178
PB.3213.1	6	-	2525	1	intergenic	novelGene_702	116650962	116653486
PB.3221.1	6	+	3549	1	intergenic	novelGene_703	117260704	117264252
PB.3227.1	7	-	2498	1	intergenic	novelGene_704	913727	916224
PB.3229.4	7	+	2016	1	intergenic	novelGene_706	2208026	2210041
PB.3229.5	7	+	1544	1	intergenic	novelGene_705	2208026	2209569
PB.3230.1	7	-	2779	1	intergenic	novelGene_707	2295727	2298505
PB.3232.3	7	+	1087	1	intergenic	novelGene_709	3382212	3383298
PB.3233.1	7	-	918	1	intergenic	novelGene_708	3262185	3263102
PB.3234.1	7	+	2466	1	intergenic	novelGene_710	3947159	3949624

PB.3235.1	7	+	3610	1	intergenic	novelGene_711	3954050	3957659
PB.3239.1	7	+	2117	1	intergenic	novelGene_712	8515510	8517626
PB.3240.27	7	-	1957	1	intergenic	novelGene_713	8633169	8635125
PB.3249.10	7	+	1556	1	intergenic	novelGene_714	15339947	15341502
PB.3256.1	7	+	2065	5	intergenic	novelGene_715	17504403	17666473
PB.3256.2	7	+	1717	3	intergenic	novelGene_716	17505108	17666475
PB.3258.1	7	+	2281	2	intergenic	novelGene_717	19773830	19776471
PB.3263.9	7	+	1469	1	intergenic	novelGene_718	22168844	22170312
PB.3264.1	7	-	2105	10	antisense	novelGene_ENSCHIG00000016206_AS	22129950	22282331
PB.3266.1	7	-	394	3	intergenic	novelGene_719	24250715	24252858
PB.3266.2	7	-	1398	1	intergenic	novelGene_720	24251473	24252870
PB.3269.1	7	+	2111	1	intergenic	novelGene_721	27204878	27206988
PB.3270.13	7	-	2735	1	intergenic	novelGene_722	27219922	27222656
PB.3273.1	7	+	1533	1	intergenic	novelGene_723	27690038	27691570
PB.3277.1	7	-	1892	1	intergenic	novelGene_724	28981553	28983444
PB.3278.1	7	-	1832	1	intergenic	novelGene_725	29244214	29246045
PB.3298.1	7	+	469	1	intergenic	novelGene_726	39885934	39886402
PB.3300.1	7	+	2170	1	intergenic	novelGene_727	40291237	40293406
PB.3316.1	7	+	1404	1	intergenic	novelGene_728	44381890	44383293
PB.3317.1	7	+	2913	1	intergenic	novelGene_729	44392385	44395297
PB.3318.1	7	-	3175	1	intergenic	novelGene_730	44638854	44642028
PB.3320.1	7	-	1963	1	intergenic	novelGene_731	44848370	44850332
PB.3323.1	7	-	1701	1	intergenic	novelGene_732	47394169	47395869
PB.3324.1	7	+	1534	1	intergenic	novelGene_733	47398639	47400172
PB.3334.1	7	-	1710	3	intergenic	novelGene_734	48262636	48282327
PB.3334.3	7	-	1551	2	intergenic	novelGene_735	48262638	48282334
PB.3338.6	7	-	1930	1	intergenic	novelGene_736	48418182	48420111
PB.3347.1	7	-	1458	1	intergenic	novelGene_737	49111831	49113288
PB.3349.1	7	-	5936	1	intergenic	novelGene_738	49224064	49229999
PB.3351.3	7	-	3044	1	intergenic	novelGene_739	49490212	49493255
PB.3352.16	7	+	1179	1	intergenic	novelGene_740	49521185	49522363
PB.3352.17	7	+	1631	1	intergenic	novelGene_741	49523273	49524903
PB.3352.18	7	+	2590	1	intergenic	novelGene_742	49533371	49535960
PB.3352.27	7	+	2653	2	intergenic	novelGene_743	49555105	49558498
PB.3352.28	7	+	1270	2	intergenic	novelGene_744	49556488	49558498
PB.3353.1	7	+	4024	1	intergenic	novelGene_745	49564445	49568468
PB.3354.1	7	-	1613	1	intergenic	novelGene_746	49603628	49605240
PB.3354.2	7	-	3019	1	intergenic	novelGene_747	49603630	49606648
PB.3355.1	7	-	3118	3	intergenic	novelGene_749	49607852	49625107
PB.3355.2	7	-	3010	2	intergenic	novelGene_748	49607852	49625104
PB.3365.1	7	+	3224	4	intergenic	novelGene_753	51323053	51337832
PB.3365.2	7	+	3181	4	intergenic	novelGene_754	51323053	51337832

PB.3365.3	7	+	2352	1	intergenic	novelGene_750	51323053	51325404
PB.3365.4	7	+	1780	4	intergenic	novelGene_752	51323053	51336388
PB.3365.5	7	+	1736	4	intergenic	novelGene_751	51323053	51336387
PB.3370.4	7	-	3287	1	intergenic	novelGene_755	53286744	53290030
PB.3374.1	7	-	2268	8	antisense	novelGene_ENSCHIG00000016782_AS	56061172	56170958
PB.3374.2	7	-	1977	1	intergenic	novelGene_756	56090043	56092019
PB.3375.1	7	+	2355	1	intergenic	novelGene_757	56171507	56173861
PB.3376.1	7	-	214	1	intergenic	novelGene_758	56412949	56413162
PB.3377.1	7	-	2120	1	intergenic	novelGene_759	56991335	56993454
PB.3379.7	7	-	2690	1	intergenic	novelGene_760	57368745	57371434
PB.3390.1	7	+	2263	1	intergenic	novelGene_761	58114756	58117018
PB.3407.2	7	+	2500	1	intergenic	novelGene_762	58997682	59000181
PB.3408.1	7	-	808	3	intergenic	novelGene_763	59064023	59134117
PB.3408.2	7	-	1972	1	intergenic	novelGene_764	59132171	59134142
PB.3418.1	7	-	4311	1	intergenic	novelGene_765	59901336	59905646
PB.3418.2	7	-	1816	5	intergenic	novelGene_767	59901336	59911550
PB.3418.3	7	-	1787	5	intergenic	novelGene_766	59901336	59911548
PB.3418.4	7	-	2468	4	intergenic	novelGene_768	59905715	59911548
PB.3420.16	7	-	2178	1	intergenic	novelGene_769	60373739	60375916
PB.3432.1	7	-	283	1	intergenic	novelGene_770	61285643	61285925
PB.3433.6	7	+	1301	1	intergenic	novelGene_771	61314666	61315966
PB.3439.2	7	-	1640	1	intergenic	novelGene_772	64189667	64191306
PB.3439.5	7	-	3734	1	intergenic	novelGene_773	64195989	64199722
PB.3451.1	7	-	2037	1	antisense	novelGene_ENSCHIG00000024204_AS	64845208	64847244
PB.3458.1	7	-	3600	1	intergenic	novelGene_774	66066024	66069623
PB.3461.5	7	-	1442	2	intergenic	novelGene_775	66250665	66256624
PB.3462.1	7	+	1566	1	intergenic	novelGene_776	66286690	66288255
PB.3471.1	7	-	1586	1	intergenic	novelGene_777	66607857	66609442
PB.3504.1	7	-	1906	1	intergenic	novelGene_778	67720065	67721970
PB.3504.2	7	-	1757	1	intergenic	novelGene_779	67720214	67721970
PB.3504.3	7	-	903	1	intergenic	novelGene_780	67721068	67721970
PB.3519.1	7	-	2138	1	antisense	novelGene_ENSCHIG00000026314_AS	71330101	71332238
PB.3536.1	7	-	2052	1	intergenic	novelGene_781	71848500	71850551
PB.3550.4	7	-	3712	1	intergenic	novelGene_782	73291612	73295323
PB.3555.1	7	-	1018	1	intergenic	novelGene_783	77342896	77343913
PB.3558.1	7	-	961	1	antisense	novelGene_ENSCHIG00000007248_AS	79641482	79642442

PB.3560.1	7	+	2232	1	antisense	novelGene_ENSCHIG00000015045_AS	79874281	79876512
PB.3564.1	7	-	1939	1	intergenic	novelGene_784	80443330	80445268
PB.3565.1	7	+	1765	4	antisense	novelGene_ENSCHIG00000026647_AS	81547131	81643704
PB.3570.1	7	-	1724	1	intergenic	novelGene_785	83694442	83696165
PB.3573.2	7	+	1492	1	intergenic	novelGene_786	84833601	84835092
PB.3578.1	7	+	1495	1	intergenic	novelGene_787	87533535	87535029
PB.3585.1	7	+	984	3	intergenic	novelGene_788	88558590	88598040
PB.3589.1	7	+	2341	6	antisense	novelGene_ENSCHIG00000014012_AS	88978597	88984898
PB.3597.3	7	-	3144	1	intergenic	novelGene_789	89223031	89226174
PB.3597.4	7	-	1846	1	intergenic	novelGene_790	89223032	89224877
PB.3602.2	7	-	2400	1	intergenic	novelGene_791	89410235	89412634
PB.3632.1	7	-	2061	1	intergenic	novelGene_792	90580502	90582562
PB.3648.7	7	-	1791	1	intergenic	novelGene_793	91253887	91255677
PB.3650.2	7	-	2615	3	antisense	novelGene_ENSCHIG00000018588_AS	91289858	91294277
PB.3650.3	7	-	2347	4	antisense	novelGene_ENSCHIG00000018588_AS	91289863	91294204
PB.3650.4	7	-	2316	2	antisense	novelGene_ENSCHIG00000018588_AS	91291774	91294269
PB.3653.1	7	-	3513	3	intergenic	novelGene_794	91784251	91791210
PB.3653.3	7	-	2257	3	intergenic	novelGene_795	91785516	91791219
PB.3653.4	7	-	2208	3	intergenic	novelGene_796	91785517	91791236
PB.3653.5	7	-	1590	3	intergenic	novelGene_797	91786162	91791198
PB.3665.1	7	+	1054	4	antisense	novelGene_ENSCHIG00000023052_AS	92095727	92116571
PB.3672.1	7	+	1360	4	antisense	novelGene_ENSCHIG00000017363_AS	92458628	92461614
PB.3672.2	7	+	2482	4	antisense	novelGene_ENSCHIG00000017363_AS	92458634	92462742
PB.3684.1	7	-	1364	1	intergenic	novelGene_798	93267185	93268548
PB.3705.1	7	+	462	5	intergenic	novelGene_799	94327167	94328655
PB.3728.1	7	-	1569	1	antisense	novelGene_ENSC	95023833	95025401

						HIG00000024731_AS		
PB.3729.1	7	+	2101	1	intergenic	novelGene_800	95041731	95043831
PB.3738.1	7	-	1972	1	intergenic	novelGene_801	95282352	95284323
PB.3754.1	7	-	3308	1	intergenic	novelGene_803	95923397	95926704
PB.3754.2	7	-	1661	1	intergenic	novelGene_802	95923397	95925057
PB.3755.1	7	-	1942	1	intergenic	novelGene_804	96068792	96070733
PB.3761.1	7	-	1326	1	intergenic	novelGene_805	96278096	96279421
PB.3779.1	7	+	2839	1	intergenic	novelGene_807	97226803	97229641
PB.3779.2	7	+	823	1	intergenic	novelGene_806	97226803	97227625
PB.3783.1	7	-	2370	3	intergenic	novelGene_808	97786758	97800467
PB.3783.2	7	-	1194	3	intergenic	novelGene_809	97787934	97800467
PB.3789.1	7	-	1775	1	intergenic	novelGene_810	97852828	97854602
PB.3806.1	7	-	2809	1	antisense	novelGene_ENSCHIG00000011017_AS	98184273	98187081
PB.3810.1	7	-	4139	4	intergenic	novelGene_811	98368484	98381024
PB.3810.2	7	-	4165	4	intergenic	novelGene_812	98368486	98381041
PB.3816.1	7	+	1913	1	intergenic	novelGene_813	100828333	100830245
PB.3817.3	7	-	3623	1	intergenic	novelGene_814	100892473	100896095
PB.3828.1	7	+	1668	2	intergenic	novelGene_815	101895695	101897809
PB.3836.1	7	+	2299	1	intergenic	novelGene_817	102312855	102315153
PB.3836.2	7	+	1874	1	intergenic	novelGene_816	102312855	102314728
PB.3852.1	7	-	1975	1	antisense	novelGene_ENSCHIG00000021205_AS	102991781	102993755
PB.3859.1	7	-	1603	2	antisense	novelGene_ENSCHIG00000020013_AS	103217950	103220064
PB.3872.1	7	+	1256	1	intergenic	novelGene_819	103850834	103852089
PB.3873.1	7	-	2144	1	intergenic	novelGene_818	103849323	103851466
PB.3893.3	7	+	2813	1	intergenic	novelGene_820	104565331	104568143
PB.3911.1	7	-	1508	1	intergenic	novelGene_821	105801239	105802746
PB.3918.1	7	+	4173	1	intergenic	novelGene_822	106324553	106328725
PB.3922.1	7	+	3976	1	antisense	novelGene_ENSCHIG00000021285_AS	107112102	107116077
PB.3925.1	7	+	2323	1	intergenic	novelGene_823	107259525	107261847
PB.3925.2	7	+	885	1	intergenic	novelGene_824	107260964	107261848
PB.3950.10	8	+	2482	1	intergenic	novelGene_825	7434765	7437246
PB.3953.2	8	-	1362	1	intergenic	novelGene_826	7831207	7832568
PB.3957.2	8	-	1925	1	intergenic	novelGene_827	8994176	8996100
PB.3958.5	8	+	1636	1	intergenic	novelGene_828	9263237	9264872
PB.3959.1	8	-	1271	1	intergenic	novelGene_829	9267752	9269022

PB.3960.1	8	-	2111	1	intergenic	novelGene_830	9297633	9299743
PB.3961.1	8	+	2892	1	intergenic	novelGene_831	9458757	9461648
PB.3961.2	8	+	1524	1	intergenic	novelGene_832	9460125	9461648
PB.3964.2	8	-	2117	1	intergenic	novelGene_833	9832530	9834646
PB.3971.5	8	+	2080	1	intergenic	novelGene_834	10874806	10876885
PB.3972.1	8	-	3197	1	intergenic	novelGene_835	10965602	10968798
PB.3977.1	8	-	1852	1	antisense	novelGene_ENSCHIG00000012805_AS_novelGene_ENSCHIG00000027260_AS	11355092	11356943
PB.3988.1	8	+	3929	1	intergenic	novelGene_836	22542863	22546791
PB.3991.1	8	+	1182	1	intergenic	novelGene_837	23521466	23522647
PB.4003.1	8	+	1567	1	intergenic	novelGene_838	27525597	27527163
PB.4004.1	8	+	1278	1	intergenic	novelGene_839	27558242	27559519
PB.4005.1	8	-	1744	1	intergenic	novelGene_840	27559151	27560894
PB.4006.1	8	+	2346	1	intergenic	novelGene_841	27563497	27565842
PB.4007.1	8	-	1467	1	intergenic	novelGene_842	27596927	27598393
PB.4008.1	8	+	1906	1	intergenic	novelGene_843	27598887	27600792
PB.4013.3	8	-	2645	3	antisense	novelGene_ENSCHIG00000012253_AS	29730591	29740256
PB.4014.1	8	-	1134	1	intergenic	novelGene_844	29967949	29969082
PB.4015.1	8	+	2604	1	intergenic	novelGene_845	29992533	29995136
PB.4016.1	8	+	1215	1	intergenic	novelGene_846	29995388	29996602
PB.4016.2	8	+	1588	1	intergenic	novelGene_847	29995868	29997455
PB.4020.1	8	+	2392	1	antisense	novelGene_ENSCHIG00000015113_AS	38249743	38252134
PB.4020.2	8	+	2032	1	antisense	novelGene_ENSCHIG00000015113_AS	38249747	38251778
PB.4021.3	8	-	2838	1	intergenic	novelGene_848	38221828	38224665
PB.4025.1	8	-	2915	1	antisense	novelGene_ENSCHIG00000024183_AS	38512834	38515748
PB.4026.1	8	-	2022	1	intergenic	novelGene_849	38595544	38597565
PB.4031.1	8	+	1880	1	antisense	novelGene_ENSCHIG00000003903_AS	39358051	39359930
PB.4034.8	8	+	1432	1	intergenic	novelGene_850	39644252	39645683
PB.4039.1	8	-	4006	1	intergenic	novelGene_851	40814941	40818946
PB.4041.1	8	+	2460	3	intergenic	novelGene_852	41707223	41769516
PB.4041.2	8	+	2449	2	intergenic	novelGene_854	41763657	41769516

PB.4041.3	8	+	1679	1	intergenic	novelGene_853	41763657	41765335
PB.4057.11	8	-	3916	1	intergenic	novelGene_855	48400429	48404344
PB.4068.1	8	-	1428	1	intergenic	novelGene_856	53512456	53513883
PB.4069.1	8	-	1397	1	intergenic	novelGene_857	53592357	53593753
PB.4072.4	8	+	2515	1	intergenic	novelGene_858	55480120	55482634
PB.4073.8	8	-	1735	1	intergenic	novelGene_859	57395272	57397006
PB.4088.1	8	+	946	1	intergenic	novelGene_860	59901422	59902367
PB.4093.2	8	-	1206	1	intergenic	novelGene_861	60180965	60182170
PB.4094.3	8	-	1344	1	intergenic	novelGene_862	60284411	60285754
PB.4095.3	8	+	1834	1	intergenic	novelGene_863	60914432	60916265
PB.4095.4	8	+	2105	1	intergenic	novelGene_864	60920540	60922644
PB.4096.1	8	-	1691	1	intergenic	novelGene_865	60924113	60925803
PB.4097.1	8	-	1254	1	intergenic	novelGene_866	60973654	60974907
PB.4100.2	8	+	1348	12	intergenic	novelGene_867	61267347	61283727
PB.4101.1	8	-	5596	2	intergenic	novelGene_869	61343972	61352499
PB.4101.2	8	-	733	3	intergenic	novelGene_868	61343972	61352486
PB.4101.3	8	-	533	2	intergenic	novelGene_870	61349021	61352485
PB.4106.4	8	+	2736	1	intergenic	novelGene_871	62277474	62280209
PB.4141.1	8	-	1591	1	intergenic	novelGene_872	69357459	69359049
PB.4145.1	8	+	2234	6	intergenic	novelGene_873	69547915	69553918
PB.4145.2	8	+	2492	5	intergenic	novelGene_874	69547959	69553918
PB.4145.3	8	+	2027	7	intergenic	novelGene_875	69547976	69553918
PB.4148.1	8	+	1924	1	intergenic	novelGene_876	69639376	69641299
PB.4149.1	8	-	1321	1	intergenic	novelGene_877	69639513	69640833
PB.4156.6	8	-	1779	1	intergenic	novelGene_878	70391665	70393443
PB.4165.6	8	+	1564	1	intergenic	novelGene_879	73334614	73336177
PB.4166.1	8	-	1296	1	intergenic	novelGene_880	73350573	73351868
PB.4167.1	8	-	1110	1	intergenic	novelGene_881	73370145	73371254
PB.4170.1	8	-	2678	1	intergenic	novelGene_882	74171691	74174368
PB.4184.1	8	-	2393	1	intergenic	novelGene_883	75049934	75052326
PB.4206.7	8	+	1888	1	intergenic	novelGene_884	77882545	77884432
PB.4211.1	8	+	1722	2	intergenic	novelGene_885	79197091	79199239
PB.4212.1	8	+	2142	1	intergenic	novelGene_886	79242260	79244401
PB.4214.1	8	-	1175	1	intergenic	novelGene_887	79871526	79872700
PB.4219.22	8	+	1397	1	intergenic	novelGene_888	81373245	81374641
PB.4219.23	8	+	2393	1	intergenic	novelGene_890	81396492	81398884
PB.4220.1	8	-	2339	1	intergenic	novelGene_889	81377301	81379639
PB.4236.3	8	+	1673	1	intergenic	novelGene_891	84245737	84247409
PB.4238.1	8	-	2399	1	antisense	novelGene_ENSCHIG00000008370_AS	84530990	84533388
PB.4244.1	8	-	1653	1	intergenic	novelGene_892	86366856	86368508
PB.4253.1	8	+	1929	1	intergenic	novelGene_893	89861974	89863902
PB.4256.1	8	+	1596	1	intergenic	novelGene_894	90132541	90134136

PB.4260.1	8	-	270	1	intergenic	novelGene_895	92665997	92666266
PB.4267.1	8	+	2349	1	intergenic	novelGene_896	95022221	95024569
PB.4268.1	8	+	1887	1	intergenic	novelGene_897	95153195	95155081
PB.4271.10	8	+	2193	1	intergenic	novelGene_898	96804722	96806914
PB.4277.1	8	+	1708	1	intergenic	novelGene_899	98622706	98624413
PB.4281.1	8	+	1341	1	intergenic	novelGene_900	99560096	99561436
PB.4285.1	8	-	1899	1	intergenic	novelGene_901	100293734	100295632
PB.4286.1	8	+	1418	1	intergenic	novelGene_902	100622182	100623599
PB.4291.1	8	-	2399	2	intergenic	novelGene_903	100941175	100947741
PB.4293.1	8	-	1290	1	intergenic	novelGene_904	101027051	101028340
PB.4294.1	8	+	1607	1	intergenic	novelGene_905	101045668	101047274
PB.4298.3	8	-	1591	1	intergenic	novelGene_906	101698213	101699803
PB.4306.3	8	-	2441	1	intergenic	novelGene_907	102357850	102360290
PB.4314.13	8	-	2916	1	intergenic	novelGene_908	104010693	104013608
PB.4318.1	8	-	2135	1	intergenic	novelGene_909	109736736	109738870
PB.4322.1	8	+	1284	1	intergenic	novelGene_910	109928448	109929731
PB.4322.2	8	+	2664	4	intergenic	novelGene_911	109928464	109945542
PB.4327.2	8	+	3613	2	intergenic	novelGene_912	110386544	110404158
PB.4336.1	8	-	2001	4	intergenic	novelGene_913	112212460	112227559
PB.4342.18	9	-	2405	2	intergenic	novelGene_914	1971843	1977169
PB.4342.19	9	-	2792	1	intergenic	novelGene_915	1974378	1977169
PB.4342.20	9	-	2192	2	intergenic	novelGene_916	1974378	1977169
PB.4346.2	9	+	2547	1	intergenic	novelGene_917	2515875	2518421
PB.4346.3	9	+	1628	1	intergenic	novelGene_918	2519274	2520901
PB.4347.1	9	-	1992	1	intergenic	novelGene_919	2536255	2538246
PB.4353.1	9	+	2180	1	antisense	novelGene_ENSCHIG00000017259_AS	6910965	6913144
PB.4360.1	9	-	1854	4	antisense	novelGene_ENSCHIG00000002547_AS	9468674	9545775
PB.4362.2	9	+	2308	1	intergenic	novelGene_920	10261131	10263438
PB.4369.4	9	-	1724	1	intergenic	novelGene_921	11296280	11298003
PB.4375.1	9	+	1516	1	intergenic	novelGene_922	15353262	15354777
PB.4376.1	9	+	2636	2	antisense	novelGene_ENSCHIG00000021420_AS	15686030	15689901
PB.4381.7	9	-	2407	1	intergenic	novelGene_923	16986517	16988923
PB.4386.1	9	-	2597	2	antisense	novelGene_ENSCHIG00000025357_AS	19631108	19641392
PB.4386.2	9	-	1780	2	antisense	novelGene_ENSCHIG00000025357_AS	19631921	19641388

PB.4386.3	9	-	1311	2	antisense	novelGene_ENSCHIG00000025357_AS	19632423	19641421
PB.4407.1	9	-	1916	1	intergenic	novelGene_924	26808729	26810644
PB.4410.2	9	-	2102	1	intergenic	novelGene_925	27724228	27726329
PB.4417.1	9	-	2720	1	intergenic	novelGene_926	28792774	28795493
PB.4417.2	9	-	2027	1	intergenic	novelGene_927	28793425	28795451
PB.4428.5	9	+	1501	1	intergenic	novelGene_928	30946949	30948449
PB.4429.1	9	+	1908	1	intergenic	novelGene_929	31037301	31039208
PB.4432.1	9	-	1051	1	intergenic	novelGene_930	31847039	31848089
PB.4437.4	9	+	4393	1	intergenic	novelGene_931	37621384	37625776
PB.4441.1	9	-	1542	1	intergenic	novelGene_932	39816280	39817821
PB.4446.1	9	+	1984	3	intergenic	novelGene_933	47246312	47470531
PB.4447.1	9	+	3133	1	intergenic	novelGene_934	47528713	47531845
PB.4453.1	9	-	2004	1	intergenic	novelGene_935	48107462	48109465
PB.4456.1	9	-	1742	1	intergenic	novelGene_936	49484288	49486029
PB.4458.1	9	-	1641	1	intergenic	novelGene_937	49538600	49540240
PB.4459.1	9	-	2996	1	intergenic	novelGene_938	49559185	49562180
PB.4468.1	9	-	2203	1	antisense	novelGene_ENSCHIG00000012016_AS	51894513	51896715
PB.4471.1	9	-	1458	1	intergenic	novelGene_939	52897723	52899180
PB.4473.4	9	-	1505	1	intergenic	novelGene_940	53489525	53491029
PB.4473.9	9	-	1489	1	intergenic	novelGene_941	53778110	53779598
PB.4475.1	9	+	2074	1	intergenic	novelGene_942	55130570	55132643
PB.4477.1	9	+	1224	1	antisense	novelGene_ENSCHIG00000010211_AS	56257876	56259099
PB.4482.1	9	+	1927	1	intergenic	novelGene_943	57118794	57120720
PB.4503.13	9	-	1047	1	intergenic	novelGene_944	63018241	63019287
PB.4505.1	9	-	1824	1	intergenic	novelGene_945	63493895	63495718
PB.4508.1	9	+	2743	1	intergenic	novelGene_946	63879646	63882388
PB.4509.1	9	-	1708	1	intergenic	novelGene_947	63964995	63966702
PB.4510.1	9	+	2163	1	intergenic	novelGene_948	63992890	63995052
PB.4513.1	9	-	2123	1	intergenic	novelGene_949	67317012	67319134
PB.4514.1	9	-	587	1	intergenic	novelGene_950	67410540	67411126
PB.4520.1	9	-	2589	1	antisense	novelGene_ENSCHIG00000013788_AS	68361164	68363752
PB.4521.5	9	-	2288	1	intergenic	novelGene_951	68411439	68413726
PB.4521.6	9	-	1927	1	antisense	novelGene_ENSCHIG00000001954_AS	68461310	68463236
PB.4524.1	9	+	2275	1	intergenic	novelGene_952	68627497	68629771

PB.4527.1	9	-	1937	1	intergenic	novelGene_953	69224931	69226867
PB.4530.1	9	-	1731	1	intergenic	novelGene_954	70918378	70920108
PB.4531.2	9	+	1787	1	intergenic	novelGene_955	71728306	71730092
PB.4532.1	9	+	2079	1	intergenic	novelGene_956	71962552	71964630
PB.4533.1	9	+	1183	1	intergenic	novelGene_957	72042842	72044024
PB.4534.1	9	-	1237	1	intergenic	novelGene_958	72254993	72256229
PB.4535.1	9	+	1920	1	intergenic	novelGene_959	72527298	72529217
PB.4536.2	9	+	2858	1	intergenic	novelGene_960	72740582	72743439
PB.4538.1	9	+	2357	1	intergenic	novelGene_961	73277151	73279507
PB.4547.1	9	-	2168	5	antisense	novelGene_ENSCHIG00000023938_AS	74151750	74159746
PB.4550.1	9	-	2630	2	intergenic	novelGene_962	74488792	74499012
PB.4553.3	9	+	1864	1	intergenic	novelGene_963	75147531	75149394
PB.4554.1	9	+	1504	1	intergenic	novelGene_964	75174860	75176363
PB.4555.2	9	+	1636	1	intergenic	novelGene_965	75264818	75266453
PB.4556.1	9	-	1851	1	intergenic	novelGene_966	75279181	75281031
PB.4570.2	9	+	1509	1	intergenic	novelGene_968	78764710	78766218
PB.4571.1	9	-	1374	1	intergenic	novelGene_967	78721374	78722747
PB.4573.1	9	+	3003	2	intergenic	novelGene_969	80724715	80775037
PB.4574.1	9	+	1673	1	intergenic	novelGene_970	80885692	80887364
PB.4575.1	9	+	1537	1	intergenic	novelGene_971	80895811	80897347
PB.4576.1	9	-	2531	1	intergenic	novelGene_972	80909475	80912005
PB.4585.11	9	-	2358	1	intergenic	novelGene_973	82465834	82468191
PB.4586.1	9	+	2911	1	antisense	novelGene_ENSCHIG00000018819_AS	82632868	82635778
PB.4597.1	9	+	1599	1	intergenic	novelGene_974	85475139	85476737
PB.4599.1	9	-	1505	1	intergenic	novelGene_975	86272402	86273906
PB.4600.1	9	+	1948	1	intergenic	novelGene_976	86276167	86278114
PB.4609.1	9	-	2628	1	antisense	novelGene_ENSCHIG00000022083_AS	89816731	89819358
PB.12122.7	LWLT01000021.1	-	2564	1	intergenic	novelGene_2471	3025251	3027814
PB.12124.1	LWLT01000021.1	+	1796	1	intergenic	novelGene_2472	3150439	3152234
PB.12134.2	LWLT01000021.1	+	1515	1	intergenic	novelGene_2473	8169679	8171193
PB.12136.1	LWLT01000	-	1972	1	intergenic	novelGene_2474	8800862	8802833

	021.1							
PB.12137.1	LWLT 01000 021.1	+	1644	4	intergenic	novelGene_2475	8857623	8864540
PB.12138.1	LWLT 01000 021.1	+	2282	1	intergenic	novelGene_2476	8865676	8867957
PB.12139.1	LWLT 01000 021.1	+	2351	1	intergenic	novelGene_2477	8868133	8870483
PB.12142.3	LWLT 01000 021.1	+	2079	1	intergenic	novelGene_2478	9869053	9871131
PB.12158.1	LWLT 01000 021.1	-	2416	1	intergenic	novelGene_2479	11893078	11895493
PB.12159.1	LWLT 01000 021.1	-	225	1	intergenic	novelGene_2480	11998506	11998730
PB.12163.1	LWLT 01000 021.1	+	1170	1	intergenic	novelGene_2481	13186309	13187478
PB.12167.1	LWLT 01000 021.1	+	1302	3	intergenic	novelGene_2482	16119846	16139881
PB.12168.1	LWLT 01000 021.1	-	2697	1	intergenic	novelGene_2483	16335155	16337851
PB.12176.5	LWLT 01000 021.1	+	4066	1	intergenic	novelGene_2484	19734044	19738109
PB.12183.1	LWLT 01000 021.1	-	2260	1	intergenic	novelGene_2485	20679846	20682105
PB.12188.1	LWLT 01000 021.1	+	1433	9	intergenic	novelGene_2486	21846940	21860955
PB.12189.1	LWLT 01000 021.1	-	2488	1	intergenic	novelGene_2487	22089468	22091955
PB.12189.2	LWLT 01000 021.1	-	2330	1	intergenic	novelGene_2488	22089609	22091938
PB.12191.2	LWLT	-	2116	1	intergenic	novelGene_2489	22996364	22998479

	01000021.1							
PB.12201.1	LWLT01000021.1	+	2713	2	intergenic	novelGene_2490	30089447	30094967
PB.12208.3	LWLT01000021.1	-	1547	1	intergenic	novelGene_2491	31533532	31535078
PB.12212.1	LWLT01000021.1	+	1673	1	intergenic	novelGene_2492	36862920	36864592
PB.12217.1	LWLT01000021.1	-	1165	1	intergenic	novelGene_2493	45877455	45878619
PB.12229.1	LWLT01000021.1	-	2070	1	intergenic	novelGene_2494	50632955	50635024
PB.12229.2	LWLT01000021.1	-	609	3	intergenic	novelGene_2495	50632955	50635024
PB.12229.4	LWLT01000021.1	-	1647	2	intergenic	novelGene_2496	50632956	50635027
PB.12229.5	LWLT01000021.1	-	1160	1	intergenic	novelGene_2497	50633867	50635026
PB.12234.1	LWLT01000021.1	+	1240	1	antisense	novelGene_ENSCHIG00000015038_AS	52722949	52724188
PB.12254.2	LWLT01000021.1	-	3186	1	intergenic	novelGene_2498	56589349	56592534
PB.12259.1	LWLT01000021.1	-	1243	2	antisense	novelGene_ENSCHIG00000012678_AS	59532275	59537722
PB.12291.2	LWLT01000027.1	-	1263	1	intergenic	novelGene_2499	13765594	13766856
PB.12299.1	LWLT01000027.1	-	2008	1	intergenic	novelGene_2500	20009214	20011221
PB.12300.1	LWLT01000027.1	-	2548	1	intergenic	novelGene_2501	20020873	20023420

PB.12301.1	LWLT 01000 027.1	-	2330	2	intergenic	novelGene_2502	20041763	20045661
PB.12312.1	LWLT 01000 027.1	-	369	3	intergenic	novelGene_2503	24555347	24558524
PB.12315.1	LWLT 01000 027.1	+	384	3	intergenic	novelGene_2504	24623643	24627008
PB.12318.7	LWLT 01000 027.1	+	1570	1	intergenic	novelGene_2505	26318610	26320179
PB.12324.1	LWLT 01000 027.1	-	4093	1	intergenic	novelGene_2506	33506757	33510849
PB.12325.1	LWLT 01000 027.1	+	2159	1	intergenic	novelGene_2507	33528405	33530563
PB.12330.1	LWLT 01000 027.1	-	1637	1	antisense	novelGene_ENSCHIG00000004049_AS	35093986	35095622
PB.12333.1	LWLT 01000 027.1	+	3179	1	intergenic	novelGene_2508	35729905	35733083
PB.12337.1	LWLT 01000 027.1	+	901	2	intergenic	novelGene_2509	37074923	37076612
PB.12344.1	LWLT 01000 027.1	-	1971	2	intergenic	novelGene_2510	37776980	37804444
PB.12353.1	LWLT 01000 027.1	+	2053	1	intergenic	novelGene_2511	40849895	40851947
PB.12362.1	LWLT 01000 033.1	-	2416	12	antisense	novelGene_ENSCHIG00000022050_AS	9547	60602
PB.12365.1	LWLT 01000 033.1	-	3437	1	intergenic	novelGene_2512	587144	590580
PB.12380.1	LWLT 01000 035.1	+	2961	3	intergenic	novelGene_2513	377016	381123
PB.12406.1	LWLT 01000	-	1941	3	intergenic	novelGene_2514	5289	12442

	039.1							
PB.12409.1	LWLT 01000 041.1	+	1460	1	intergenic	novelGene_2515	208624	210083
PB.12425.1	LWLT 01000 051.1	-	2577	1	intergenic	novelGene_2516	355552	358128
PB.12439.2	LWLT 01000 066.1	+	2093	1	intergenic	novelGene_2517	198031	200123
PB.12455.1	LWLT 01000 075.1	-	2600	1	intergenic	novelGene_2518	179150	181749
PB.12455.2	LWLT 01000 075.1	-	2071	1	intergenic	novelGene_2519	180076	182146
PB.12463.1	LWLT 01000 090.1	-	1682	1	intergenic	novelGene_2520	143988	145669
PB.12469.1	LWLT 01000 099.1	+	1580	1	intergenic	novelGene_2521	15761	17340
PB.12495.1	LWLT 01000 123.1	-	2275	1	intergenic	novelGene_2522	76934	79208
PB.12509.1	LWLT 01000 132.1	-	2539	2	intergenic	novelGene_2527	3575	7290
PB.12509.2	LWLT 01000 132.1	-	2402	2	intergenic	novelGene_2528	3709	7287
PB.12509.3	LWLT 01000 132.1	-	1718	2	intergenic	novelGene_2529	3709	7424
PB.12529.4	LWLT 01000 155.1	-	2208	1	intergenic	novelGene_2530	32779	34986
PB.12530.1	LWLT 01000 161.1	+	1118	5	intergenic	novelGene_2531	23699	41568
PB.12534.16	LWLT 01000 164.1	-	2745	1	intergenic	novelGene_2532	26089	28833
PB.12554.1	LWLT	+	807	1	intergenic	novelGene_2533	4615	5421

	01000 225.1							
PB.12554.2	LWLT 01000 225.1	+	698	1	intergenic	novelGene_2534	4943	5640
PB.12555.1	LWLT 01000 240.1	+	1859	1	intergenic	novelGene_2535	7810	9668
PB.12561.1	LWLT 01000 259.1	+	1637	1	antisense	novelGene_ENSCHIG00000000336_AS_novelGene_ENSCHIG00000000299_AS	22082	23718
PB.12577.1	LWLT 01000 442.1	+	4816	1	intergenic	novelGene_2537	16581	21396
PB.12583.1	LWLT 01000 480.1	-	1577	1	intergenic	novelGene_2538	13888	15464
PB.12591.1	LWLT 01000 494.1	-	2377	7	intergenic	novelGene_2548	9624	13675
PB.12591.2	LWLT 01000 494.1	-	1506	7	intergenic	novelGene_2549	10481	13661
PB.12592.1	LWLT 01000 497.1	+	305	1	intergenic	novelGene_2550	21124	21428
PB.12596.1	LWLT 01000 516.1	+	860	1	intergenic	novelGene_2553	12033	12892
PB.12596.2	LWLT 01000 516.1	+	878	1	intergenic	novelGene_2554	12181	13058
PB.12596.3	LWLT 01000 516.1	+	987	1	intergenic	novelGene_2555	12233	13219
PB.12602.1	LWLT 01000 526.1	-	2764	3	intergenic	novelGene_2561	5565	19715
PB.12602.2	LWLT 01000 526.1	-	2850	4	intergenic	novelGene_2562	5566	19723
PB.12615.1	LWLT	+	1026	1	intergenic	novelGene_2575	8448	9473

	01000 567.1							
PB.12503.1	LWLT 01000 684.1	+	2172	5	intergenic	novelGene_2523	44739	50447
PB.12503.2	LWLT 01000 684.1	+	2258	5	intergenic	novelGene_2524	44756	50454
PB.12503.3	LWLT 01000 684.1	+	2028	4	intergenic	novelGene_2525	44758	50456
PB.12505.1	LWLT 01000 684.1	+	1031	1	intergenic	novelGene_2526	71483	72513
PB.12576.1	LWLT 01000 973.1	+	2954	1	intergenic	novelGene_2536	17489	20442
PB.12584.1	LWLT 01001 387.1	-	2161	1	intergenic	novelGene_2539	8552	10712
PB.12585.1	LWLT 01001 401.1	+	1074	1	intergenic	novelGene_2540	15723	16796
PB.12586.1	LWLT 01001 513.1	-	1561	4	intergenic	novelGene_2541	1999	14876
PB.12586.2	LWLT 01001 513.1	-	1210	3	intergenic	novelGene_2542	2265	14870
PB.12587.1	LWLT 01001 533.1	-	1634	1	intergenic	novelGene_2543	1664	3297
PB.12590.1	LWLT 01001 643.1	+	2797	5	intergenic	novelGene_2544	1031	16720
PB.12590.2	LWLT 01001 643.1	+	3059	5	intergenic	novelGene_2545	1061	17012
PB.12590.4	LWLT 01001 643.1	+	2273	4	intergenic	novelGene_2546	5288	16720
PB.12590.5	LWLT 01001 643.1	+	2451	2	intergenic	novelGene_2547	8823	16720

PB.12594.1	LWLT01001786.1	-	1924	1	intergenic	novelGene_2551	13830	15753
PB.12595.1	LWLT01001803.1	+	1345	4	intergenic	novelGene_2552	7843	20452
PB.12598.1	LWLT01001916.1	-	1779	4	intergenic	novelGene_2556	2202	15246
PB.12598.2	LWLT01001916.1	-	1652	4	intergenic	novelGene_2557	2330	15247
PB.12599.1	LWLT01002021.1	+	2067	16	intergenic	novelGene_2558	12178	16986
PB.12600.1	LWLT01002024.1	-	1347	4	intergenic	novelGene_2559	2130	14742
PB.12601.1	LWLT01002055.1	-	1602	4	intergenic	novelGene_2560	6720	19642
PB.12603.1	LWLT01002062.1	+	1165	1	intergenic	novelGene_2563	9450	10614
PB.12604.1	LWLT01002066.1	-	1427	5	intergenic	novelGene_2564	5854	18464
PB.12606.1	LWLT01002103.1	+	1181	4	intergenic	novelGene_2565	4772	17274
PB.12606.2	LWLT01002103.1	+	2067	1	intergenic	novelGene_2566	4785	6851
PB.12607.1	LWLT01002115.1	+	1585	1	intergenic	novelGene_2567	8199	9783
PB.12608.1	LWLT01002319.1	+	1284	4	intergenic	novelGene_2568	5799	18407
PB.12609.1	LWLT01002373.1	-	1264	3	intergenic	novelGene_2569	7477	14101
PB.12611.1	LWLT01002	-	3169	4	intergenic	novelGene_2570	1658	16079

	709.1							
PB.12611.2	LWLT 01002 709.1	-	2903	4	intergenic	novelGene_2571	1924	16079
PB.12611.3	LWLT 01002 709.1	-	2725	4	intergenic	novelGene_2572	2047	16079
PB.12612.1	LWLT 01002 833.1	+	727	1	intergenic	novelGene_2573	10439	11165
PB.12613.1	LWLT 01002 833.1	-	165	1	intergenic	novelGene_2574	18470	18634
PB.12618.1	LWLT 01003 260.1	+	1219	4	intergenic	novelGene_2576	801	13284
PB.12618.2	LWLT 01003 260.1	+	1671	4	intergenic	novelGene_2577	807	13797
PB.12618.3	LWLT 01003 260.1	+	1595	3	intergenic	novelGene_2578	807	13800
PB.12619.1	LWLT 01003 675.1	-	1604	4	intergenic	novelGene_2579	846	13750
PB.12619.2	LWLT 01003 675.1	-	1470	3	intergenic	novelGene_2580	846	13750
PB.12622.1	LWLT 01005 313.1	+	2923	4	intergenic	novelGene_2581	992	15200
PB.12622.2	LWLT 01005 313.1	+	1699	5	intergenic	novelGene_2582	994	15200
PB.12624.1	LWLT 01005 359.1	-	1186	1	intergenic	novelGene_2583	1828	3013
PB.12625.1	LWLT 01006 476.1	-	1752	4	intergenic	novelGene_2584	1287	14305
PB.12626.1	LWLT 01007 538.1	+	2168	1	intergenic	novelGene_2585	4679	6846
PB.12628.1	LWLT	-	1866	1	intergenic	novelGene_2586	3720	5585

	01011 582.1							
PB.12629.1	LWLT 01011 611.1	-	2159	1	intergenic	novelGene_2587	4638	6796
PB.12630.1	LWLT 01013 283.1	-	1074	1	intergenic	novelGene_2588	4187	5260
PB.12631.1	LWLT 01015 557.1	-	2302	5	intergenic	novelGene_2589	1774	4398
PB.12631.2	LWLT 01015 557.1	-	2290	5	intergenic	novelGene_2590	1774	4398
PB.12631.3	LWLT 01015 557.1	-	2255	6	intergenic	novelGene_2591	1774	4398
PB.12633.1	LWLT 01019 622.1	+	1540	1	intergenic	novelGene_2592	126	1665
PB.12112.1	MT	-	387	1	antisense	novelGene_ENSCHIG00000000004_AS	1471	1857
PB.12116.1	MT	-	1829	1	antisense	novelGene_ENSCHIG00000000028_AS_novelGene_ENSCHIG00000000027_AS_novelGene_ENSCHIG00000000025_AS	9657	11485
PB.12118.1	MT	+	1278	1	intergenic	novelGene_2469	15366	16643
PB.12118.2	MT	+	213	1	intergenic	novelGene_2470	16431	16643

Table S5: Isoform classification statistical table.

Classification	Number
FSM （Full Splice Match）	8412
ISM （Incomplete Splice Match）	2933
NIC（Novel In Catalog）	2936
NNC（Novel Not in Catalog）	15,233
Genic Genomic	1497
Antisense	276
Intergenic	2589
Genic Intron	3

Table S6: Summary of ORF forecast results.

ORF	Number
Total number of isoforms	34,253
Number of isoforms predicted with ORF	30,947
Number of isoforms with complete ORF structure detected	22,200
Number of isoforms predicted with ORF but lacking stop codon at 3' end	1680
Number of isoforms predicted with ORF but lacking start codon at 5' end	6774
Number of isoforms with internal sequences only	293

Table S7: Lists the notes of each database.

Type of database	Number of annotated isoforms successfully aligned to the database	Percentage of annotated isoforms successfully aligned to the database out of the total isoforms (%)
NR	30,460	98.43
GO	29,188	94.32
KEGG	22,265	71.95
Pfam	26,842	86.74
eggNOG	30,190	97.55
Swissprot	29,729	96.06
Number of isoforms annotated in all of the above databases	20,066	64.84

Table S8: Notes summary results.

Classification	Number
Isoform	34,253
Known lncRNA	32
Novel lncRNA	256

Table S9: Location information of newly identified Known lncRNA

Known lncRNA	Start	End
ENSCHIG00000000739	40064408	40105597
ENSCHIG00000000812	56382169	56390184
ENSCHIG00000000895	26269015	26282118
ENSCHIG00000001289	29083934	29319846
ENSCHIG00000001304	41894357	42000754
ENSCHIG00000001369	5391586	5398532
ENSCHIG00000001661	98955453	98971414
ENSCHIG00000001905	49493171	49586659
ENSCHIG00000002007	56103438	56121082
ENSCHIG00000002339	78902289	78912793
ENSCHIG00000003917	29676772	29703713
ENSCHIG00000004286	25036630	25081904
ENSCHIG00000004467	47000000	47002790
ENSCHIG00000004478	51608899	51641779
ENSCHIG00000004657	12707457	12711698
ENSCHIG00000005310	13241427	13245898
ENSCHIG00000005368	25458014	25464491

ENSCHIG00000005552	57707303	57722542
ENSCHIG00000005603	65744848	65749499
ENSCHIG00000005670	20721770	20751291
ENSCHIG00000005803	20400070	20407055
ENSCHIG00000005980	83446530	83521199
ENSCHIG00000006309	31276362	31279845
ENSCHIG00000006942	88935308	88985445
ENSCHIG00000007001	3122641	3194322
ENSCHIG00000007159	53516269	53532733
ENSCHIG00000007521	106685149	106836364
ENSCHIG00000007579	1958348	1973230
ENSCHIG00000007646	9775295	9935633
ENSCHIG00000007670	9775295	9935633
ENSCHIG00000007864	2055958	2177545
ENSCHIG00000007933	52349189	52500550

Table S10: Location information of newly identified novel lncRNAs

**Isoform_ID**	**Start**	**End**
PB.86.1	43906467	43907975
PB.191.3	70890483	70892494
PB.223.1	78153140	78154784
PB.262.8	83166373	83168839
PB.327.1	110106681	110109073
PB.364.1	124465224	124479035
PB.364.2	124465245	124479042
PB.390.1	130284685	130286307
PB.415.2	136835106	136839012
PB.482.1	145964394	145966200
PB.491.1	146939242	146941231
PB.544.2	1119979	1122287
PB.547.1	2296750	2298999
PB.550.1	2447629	2450965
PB.725.1	16324324	16326841
PB.871.1	50396577	50398836
PB.971.1	100087702	100090094
PB.1020.1	114120134	114124150
PB.1092.1	135390647	135392622
PB.1098.2	349097	351382
PB.1112.1	1110165	1113160
PB.1123.2	3182218	3186561
PB.1206.3	14202648	14204961
PB.1333.8	29338542	29340223
PB.1558.1	94232500	94234419
PB.1616.1	100045049	100046569
PB.1661.1	102361878	102362997

PB.1661.2	102361878	102362997
PB.1662.2	102365711	102367451
PB.1671.1	102762522	102764729
PB.1839.1	119004458	119007097
PB.1849.1	1199595	1203912
PB.1919.1	17372543	17376507
PB.1919.2	17373928	17376507
PB.2044.1	52216524	52219160
PB.2208.1	112938738	112940612
PB.2299.13	26676904	26680019
PB.2423.6	45857492	45860100
PB.2450.1	55063052	55064900
PB.2532.14	64124938	64127000
PB.2559.3	68944956	68949253
PB.2559.4	68946884	68949233
PB.2573.1	72575817	72578193
PB.2646.1	89957322	89959107
PB.2680.9	97563377	97565236
PB.2749.2	104680843	104684097
PB.2749.3	104680843	104683021
PB.2752.2	105479447	105481381
PB.2765.1	106633152	106635312
PB.2816.1	109514420	109516682
PB.2823.2	110183112	110185779
PB.2837.6	111481179	111483246
PB.2877.1	115063063	115064843
PB.3211.1	116648582	116650178
PB.3349.1	49224064	49229999
PB.3352.21	49542375	49544815
PB.3355.1	49607852	49625107
PB.3355.2	49607852	49625104
PB.3462.1	66286690	66288255
PB.3552.1	75975507	75989147
PB.3552.4	75975555	75989147
PB.3597.3	89223031	89226174
PB.3623.1	90253797	90256648
PB.3684.1	93267185	93268548
PB.3729.1	95041731	95043831
PB.3873.1	103849323	103851466
PB.3924.3	107166940	107170834
PB.3924.23	107193969	107198079
PB.3924.24	107194281	107198079
PB.4005.1	27559151	27560894
PB.4177.1	74659008	74661229

PB.4240.1	85529011	85533840
PB.4306.3	102357850	102360290
PB.4314.13	104010693	104013608
PB.4342.19	1974378	1977169
PB.4447.1	47528713	47531845
PB.4614.1	90998854	91000794
PB.4629.1	1038504	1040347
PB.4641.7	5986030	5988116
PB.4711.2	21665751	21667991
PB.4761.10	31694418	31696133
PB.4836.1	50165468	50180370
PB.4846.9	52189076	52190930
PB.4855.1	53833572	53835343
PB.5117.1	91494090	91544715
PB.5160.1	718475	858774
PB.5209.1	6550005	6552460
PB.5313.1	26310240	26311465
PB.5314.1	26318254	26320347
PB.5318.1	28319087	28320966
PB.5323.1	29099672	29102463
PB.5431.10	62296303	62299440
PB.5473.5	69351088	69352436
PB.5478.1	70710306	70712559
PB.5623.2	97859312	97861556
PB.5670.1	99588181	99590324
PB.5675.2	99694370	99696482
PB.5703.3	102420334	102423367
PB.5706.7	102596180	102598513
PB.5719.3	103546845	103550681
PB.5719.5	103546846	103550681
PB.5721.1	103655001	103660383
PB.5721.2	103655006	103660388
PB.5733.1	104172741	104176064
PB.5733.2	104172742	104176065
PB.5745.1	105208513	105211411
PB.5754.1	105913592	105919361
PB.5911.1	64740660	64742732
PB.5975.5	75406781	75409316
PB.5999.2	10918722	11001122
PB.6105.1	41211737	41214706
PB.6157.3	50812769	50816797
PB.6186.1	53225686	53227749
PB.6204.2	54012148	54015069
PB.6274.1	62730134	62732113

PB.6336.1	69402829	69405033
PB.6341.2	71770919	71772108
PB.6346.9	72468928	72470161
PB.6354.1	73014954	73022843
PB.6522.1	35784792	35786948
PB.6579.1	54058285	54059824
PB.6597.1	59008804	59010529
PB.6620.2	65572844	65575010
PB.6646.3	73804022	73806870
PB.6652.1	78983623	78986587
PB.6679.1	81178702	81182142
PB.6742.1	2447818	2456912
PB.6833.1	26967652	26969389
PB.6950.1	46884680	46911258
PB.7065.1	67070028	67072468
PB.7109.1	456609	462239
PB.7110.1	476350	478264
PB.7124.1	2332499	2334159
PB.7148.9	4197232	4201678
PB.7284.1	42698681	42701094
PB.7288.1	43172578	43174510
PB.7325.1	49455756	49457691
PB.7461.6	72255766	72257383
PB.7464.2	72530096	72533410
PB.7488.2	78618806	78622077
PB.7494.1	79280488	79286651
PB.7499.1	69885	72316
PB.7513.1	1252060	1254723
PB.7513.2	1252130	1254723
PB.7545.1	2956421	2958763
PB.7566.6	6788578	6790240
PB.7701.2	24978262	24981683
PB.7729.1	31237209	31239094
PB.7757.1	53517992	53519711
PB.7782.1	64450946	64533803
PB.7821.1	3173589	3176085
PB.7825.1	3386789	3399223
PB.7848.1	7741458	7745785
PB.7848.2	7741621	7743818
PB.7848.3	7741622	7745569
PB.7859.1	9337459	9339578
PB.7860.1	9388090	9389898
PB.8083.17	40406056	40410012
PB.8095.1	43083208	43086895

PB.8182.1	49789732	49800855
PB.8223.2	51648341	51649756
PB.8224.2	51679365	51685258
PB.8259.4	53509214	53511174
PB.8260.1	53551255	53553449
PB.8311.1	55149662	55151833
PB.8377.5	57275977	57277626
PB.8565.1	10737876	10739764
PB.8640.2	19928735	19931299
PB.8696.1	23171301	23174186
PB.8813.10	27789704	27792383
PB.8831.1	30437828	30441336
PB.8836.10	32451186	32453332
PB.8877.4	34778453	34780851
PB.9050.1	42248795	42251485
PB.9111.2	44580250	44584299
PB.9111.3	44581422	44584541
PB.9143.1	47881796	47883175
PB.9188.1	50392197	50395186
PB.9228.1	52774444	52779858
PB.9231.1	53049204	53052419
PB.9309.2	57707320	57709156
PB.9309.3	57707321	57864985
PB.9322.1	61117148	61118521
PB.9330.1	502193	504668
PB.9389.1	12426736	12428861
PB.9448.1	30469383	30472365
PB.9497.1	41271362	41273875
PB.9508.1	57170617	57173049
PB.9562.1	7339856	7342143
PB.9567.1	13306745	13309082
PB.9573.4	15828336	15831062
PB.9660.1	27286416	27288350
PB.9661.1	27296388	27299459
PB.9670.1	29973474	29976037
PB.9881.6	6822780	6824995
PB.9924.1	14341125	14344096
PB.10030.11	43207031	43208736
PB.10073.1	48819014	48822266
PB.10112.11	50734482	50738152
PB.10165.1	53274965	53282503
PB.10171.1	53603018	53606158
PB.10185.3	56004475	56005706
PB.10210.3	58965041	58966911

PB.10224.3	59657373	59659223
PB.10306.4	17051227	17055262
PB.10309.1	17495043	17528192
PB.10324.1	19391791	19394381
PB.10416.2	23935456	23939029
PB.10459.11	32114200	32115634
PB.10579.1	2579534	2582591
PB.10580.1	2665812	2667698
PB.10584.7	4025539	4026728
PB.10608.15	22377456	22380137
PB.10611.1	25059751	25061774
PB.10652.1	37516518	37518612
PB.10742.2	61911519	61914011
PB.10757.1	211851	214372
PB.10856.1	3864513	3866428
PB.11040.1	28141908	28144618
PB.11044.1	29802724	29803887
PB.11067.3	33626266	33628144
PB.11071.1	33944902	33946993
PB.11071.2	33944927	33947954
PB.11071.3	33944936	33948074
PB.11102.1	36779740	36781679
PB.11183.2	41769248	41771593
PB.11241.1	12398273	12401132
PB.11318.1	28870194	28872354
PB.11377.1	34405808	34409236
PB.11377.2	34406388	34409407
PB.11430.1	43417567	43419145
PB.11438.1	3107330	3109930
PB.11465.1	8553348	8555420
PB.11473.1	9496722	9499187
PB.11495.7	12659696	12661776
PB.11504.4	17142862	17144070
PB.11510.5	19293153	19299483
PB.11648.2	16227742	16233709
PB.11669.9	18625665	18627597
PB.11682.1	19937658	19940150
PB.11734.2	36587966	36687270
PB.11781.12	6811608	6816073
PB.11793.2	9164028	9167283
PB.11820.1	17960090	17962150
PB.11865.6	31270692	31274464
PB.11954.1	43534095	43536495
PB.12087.1	49318165	49322641

PB.12102.1	50438597	50441324
PB.12102.2	50438918	50441324
PB.12168.1	16335155	16337851
PB.12418.5	542850	554499
PB.12425.1	355552	358128
PB.12455.2	180076	182146
PB.12543.3	3469	7205
PB.12585.1	15723	16796
PB.12626.1	4679	6846

Table S11: Variable shear event statistics.

**Sample**	**Intron Retention**	**Exon Skipping**	**lternative 5' Splice Site**	**Alternative 3' Splice Site**	**Total**
Number	2861	3954	2973	2495	12,283

Table S12: Statistical table of APA sites.

**Sample**	**1**	**3**	**4**	**5**	**>5**
Number	152	63	52	32	132
